# Supplementary material for: An electric molecular motor
Source: Nature. 2023 Jan 11;613(7943):280–6. doi: 10.1038/s41586-022-05421-6 (PMC9834048; doi:10.1038/s41586-022-05421-6)
Supplement: Supplementary file 1 — This file contains Supplementary Figs. 1–78, Schemes 1–9, Tables 1–11, supplementary text and notes, extensive experimental data, quantum mechanical calculation results and detailed discussions. [file 41586_2022_5421_MOESM1_ESM.pdf]

nature portfolio

https://doi.org/10.1038/s41586-022-05421-6

Supplementary information

An electric molecular motor

In the format provided by the authors and unedited

Supplementary Information

An electric molecular motor

Long Zhang*, Yunyan Qiu, Wei-Guang Liu, Hongliang Chen, Dengke Shen, Bo Song, Kang Cai, Huang Wu, Yang Jiao, Yuanning Feng, James S. W. Seale, Cristian Pezzato, Jia Tian, Yu Tan, Xiao-Yang Chen, Qing-Hui Guo, Charlotte L. Stern, Douglas Philp, R. Dean Astumian*, William A. Goddard III*, J. Fraser Stoddart*

*Correspondence to: long.zhang@northwestern.edu; astumian@maine.edu; wag@caltech.edu; stoddart@northwestern.edu

Table of Contents

1. Materials and General Methods S2

2. Synthetic Protocols S4

3. NMR Spectroscopy S14

4. Mass Spectrometry S46

5. The [2]Catenane Test S48

6. Quantum Mechanical Calculations S49

6.1 [2]Catenane S50

6.2 [3]Catenane S54

7. X-Ray Crystallography S68

8. Cyclic Voltammetry........ S71

9. Vis/NIR Absorption Spectroscopy S74

10. Electrically Driven Operation of [3]CMM S75

11. Chemically Driven Operation of [3]CMM S78

12. Measurements of the Directionality S79

13. Metastable State and Kinetic Studies........ S96

14. References S111

1. Materials and General Methods

Chemicals were purchased as reagent grade and employed without further purification. Commercial grades of anhydrous solvents including acetonitrile (MeCN), *N*, *N*-dimethylformamide (DMF) and diethyl ether (Et_2O) were used as received. The compounds, cyclobis(paraquat-*p*-phenylene) tetrakis(hexafluorophosphate)[1] (**CBPQT**•4PF_6), in addition to the precursors **S1**•2PF_6[2], 4-(bromomethyl)-2-(1-methylethyl)-1-(2-propyn-1-yloxy)-benzene[3], bis[4-(bromomethyl)phenyl]methane[4] and [D_8]-*p*-xylylene dibromide[5] were prepared according to literature procedures. Column chromatography, including both normal phase (RediSep Rf Gold® Normal-Phase Silica) and reversed-phase (RediSep Rf Gold® Reversed-Phase C18), were carried out using CombiFlash® Automation Systems (Teledyne ISCO). For Vis/Near Infrared (NIR) spectroscopic studies, all sample preparations were performed in an N_2-filled atmosphere. Samples were loaded into quartz 1-cm tubes and sealed and used immediately after preparation. Nuclear magnetic resonance (NMR) spectra were recorded on a Bruker Neo 600 MHz spectrometer equipped with QCI-F cryoprobe (^{1}H sensitivity = 5000), Bruker Avance III 500 and 600 spectrometers, with working frequencies of 500 and 600 MHz for ^{1}H, and 125 MHz and 150 MHz for ^{13}C nuclei, respectively. Chemical shifts are reported in ppm relative to the signals corresponding to the residual non-deuterated solvents (CD_3CN: δ_H = 1.94 ppm, and δ_C = 118.26 ppm, D_2O: δ_H = 4.79 ppm, CD_3COCD_3: δ_H = 2.05 ppm, and δ_C = 29.84 ppm). Electrospray ionization mass spectrometry (ESI-MS) and traveling wave ion mobility mass spectrometry (TWIM-MS) were conducted on a Waters Synapt G2 mass spectrometer, using a 0.5 mg/mL solution of sample in acetonitrile injected with direct infusion. The TWIM-MS was performed under the following conditions: ESI capillary voltage, 3 kV; sample cone voltage, 30 V; extraction cone voltage, 3.5 V; source temperature 100 ºC; desolvation temperature, 100 ºC; cone gas flow, 10 L/h; desolvation gas flow, 700 L/h (N_2); source gas control, 0 mL/min; trap gas control, 2 mL/min; helium cell gas control, 150 mL/min; ion mobility (IM) cell gas control, 30 mL/min;

sample flow rate, 5 μL/min; IM traveling wave height, 25 V; and IM traveling wave velocity, 1000 m/s. Quadrupole was set in rf-only mode to transmit all ions produced by ESI into the travelling wave region and TOF analyzer for the acquisition of ion mobility and mass spectrometric data. The calibration procedure of Scrivens et al.,[6] was used to convert the drift time scale of the TWIM-MS experiments to a collision cross-section (CCS) scale. The calibration curve was constructed by plotting the corrected CCSs of the molecular ions of myoglobin against the corrected drift times of the corresponding molecular ions measured in TWIM-MS experiments at the same traveling wave velocity, traveling wave height and ion mobility gas flow settings viz., 1000 m/s, 25 V, and 30 mL/min, respectively. The database for CCSs of myoglobin was obtained from Clemmer group's webpage[7]. The theoretical CCS for the [3]catenane **[3]CMM** was calculated with Ion Mobility Spectrometry Suite (IMoS) software using trajectory method. High-resolution mass spectra (HRMS) were recorded on an Agilent 6210 Time-of-Flight (TOF) LC-MS, using an electrospray ionization (ESI) source, coupled with an Agilent 1100 HPLC stack. The samples were injected via direct infusion at a flow rate of 0.6 mL/min. Measurements at X-band (9.5 GHz) were performed with a Bruker Elexsys E580, equipped with a variable Q dielectric resonator (ER-4118X-MD5-W1). Cyclic voltammetry (CV) was carried out at room temperature in N_2-purged acetonitrile (MeCN) solutions with a Gamry Multipurpose instrument (Reference 600) interfaced to a PC. A three-electrode system was used to record the data, in which the working electrode was a glassy carbon (0.071 cm^2), the counter electrode was a Pt wire, and the reference electrode was a Ag/AgCl electrode. The surface of working electrode was polished routinely with 0.05 μm alumina-water slurry on a felt surface immediately before use. Tetrabutylammonium hexafluorophosphate ($TBAPF_6$) was used as supporting electrolyte with the concentration of 0.1 M.

2. Synthetic Protocols

Supplementary Scheme 1 | Synthesis of S2•3PF6 from S1•2PF6

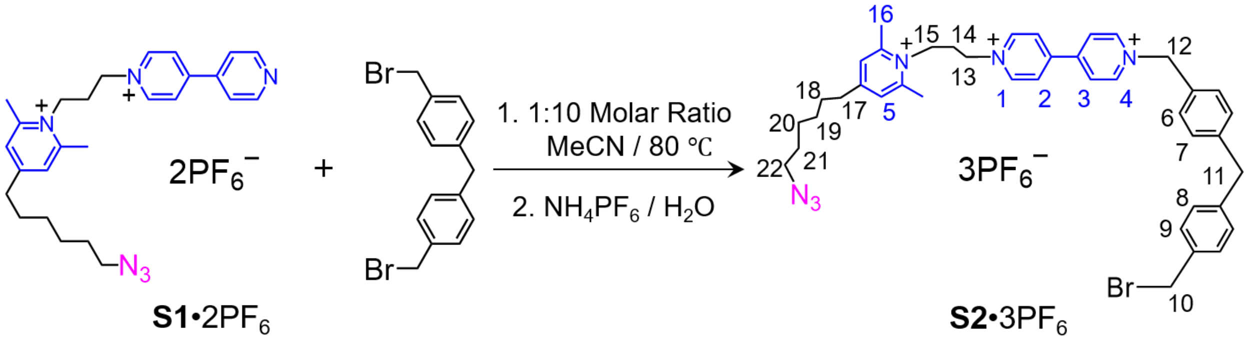

S2•3PF6: A solution of **S1**•$2PF_6$ (216 mg, 0.3 mmol) in MeCN (40 mL) was added dropwise by syringe at 2 mL/h to a solution of bis[4-(bromomethyl)phenyl]methane (1.06 g, 3 mmol) in MeCN (40 mL) at 80 °C. The reaction mixture was stirred for a further 72 h under reflux, and then cooled to room temperature. An excess of NH_4PF_6 was added to this solution, and the solvent was removed under reduced pressure. The residue was washed with CH_2Cl_2 and H_2O to yield **S2**•$3PF_6$ as a pale white solid (276 mg, 81%).

^{1}H NMR (500 MHz, CD_3CN) δ 8.95 (d, J = 6.5 Hz, 2H, H_1), 8.93 (d, J = 7.0 Hz, 2H, H_4), 8.39 (d, J = 7.0 Hz, 2H, H_3), 8.35 (d, J = 6.5 Hz, 2H, H_2), 7.57 (s, 2H, H_5), 7.43 (d, J = 8.0 Hz, 2H, H_6), 7.37 (d, J = 8.0 Hz, 2H, H_7), 7.35 (d, J = 8.5 Hz, 2H, H_8), 7.22 (d, J = 8.0 Hz, 2H, H_9), 5.78 (s, 2H, H_{12}), 4.78 (t, J = 8.0 Hz, 2H, H_{13}), 4.56 (s, 2H, H_{10}), 4.47(t, J = 8.5 Hz, 2H, H_{15}), 4.00 (s, 2H, H_{11}), 3.29 (t, J = 7.0 Hz, 2H, H_{22}), 2.79 – 2.76(m, 8H, $H_{16,17}$), 2.50 – 2.43 (m, 2H, H_{14}), 1.68 (quint, J = 7.5 Hz, 2H, H_{18}), 1.57 (quint, J = 7.0 Hz, 2H, H_{21}), 1.43 – 1.34 (m, 4H, $H_{19,20}$). ^{13}C NMR (125 MHz, CD_3CN) δ 163.7, 155.7, 151.4, 151.1, 146.8, 146.5, 144.7, 142.4, 137.4, 131.4, 130.8, 130.6, 130.4, 130.1, 129.0, 128.4, 65.5, 59.1, 52.0, 49.5, 41.6, 35.5, 34.6, 29.9, 29.7, 29.2, 29.1, 26.9, 21.4. **HRMS-ESI** (*m/z*): calcd. for $[C_{41}H_{48}BrF_{18}N_6P_3 - 2PF_6 - H]^+$ 849.2667, found 849.2725; calcd. for $[C_{41}H_{48}BrF_{18}N_6P_3 - 3PF_6 - 2H]^+$ 701.2962, found 701.2988.

Supplementary Scheme 2 | Synthesis of S3•PF_6

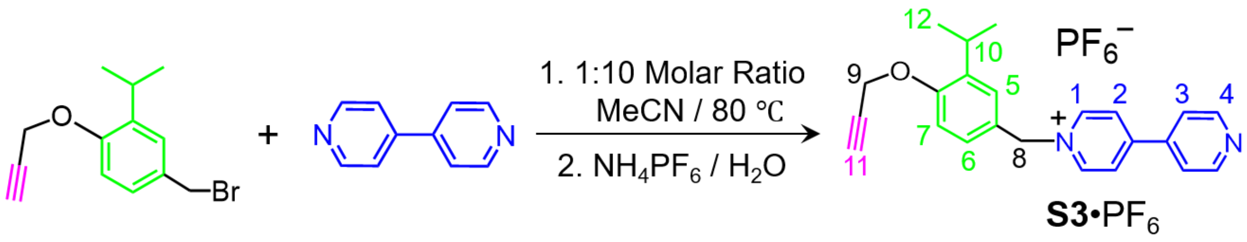

S3•PF₆: 4-(Bromomethyl)-2-(1-methylethyl)-1-(2-propyn-1-yloxy)-benzene (267 mg, 1 mmol) was added to a refluxing solution of 4,4′-bipyridine (1.56 g, 10 mmol) in MeCN (20 mL) at 80 °C. The reaction mixture was stirred for a further 24 h under reflux, and then cooled to room temperature. An excess of NH_4PF_6 was added to this solution, and the solvent was removed under reduced pressure. The residue was washed with CH_2Cl_2 and H_2O to yield **S3**•PF_6 as a white solid (391 mg, 80%).

^{1}**H NMR** (500 MHz, CD_3CN) δ 8.84 (d, J = 6.2 Hz, 2H, H_1), 8.81 (d, J = 6.9 Hz, 2H, H_4), 8.28 (d, J = 6.9 Hz, 2H, H_3), 7.77 (d, J = 6.2 Hz, 2H, H_2), 7.41 (d, J = 2.4 Hz, 1H, H_5), 7.32 (dd, J = 8.4, 2.4 Hz, 1H, H_6), 7.08 (d, J = 8.4 Hz, 1H, H_7), 5.65 (s, 2H, H_8), 4.80 (d, J = 2.4 Hz, 2H, H_9), 3.30 (hept, J = 6.9 Hz, 1H, H_{10}), 2.81 (t, J = 2.4 Hz, 1H, H_{11}), 1.21 (d, J = 6.9 Hz, 6H, H_{12}). ^{13}**C NMR** (125 MHz, CD_3CN): δ 156.8, 155.4, 152.1, 145.6, 142.1, 139.6, 129,0, 128.8, 127.1, 126.5, 122.8, 113.7, 79.6, 76.9, 65.0, 56.8, 27.7, 22.7. **HRMS-ESI** (m/z): calcd. for $[C_{23}H_{23}F_6N_2OP - PF_6]^+$ 343.1805, found 343.1803.

Supplementary Scheme 3 | Synthesis of DB•$5PF_6$ **from S2•**$3PF_6$ and **S3•**PF_6

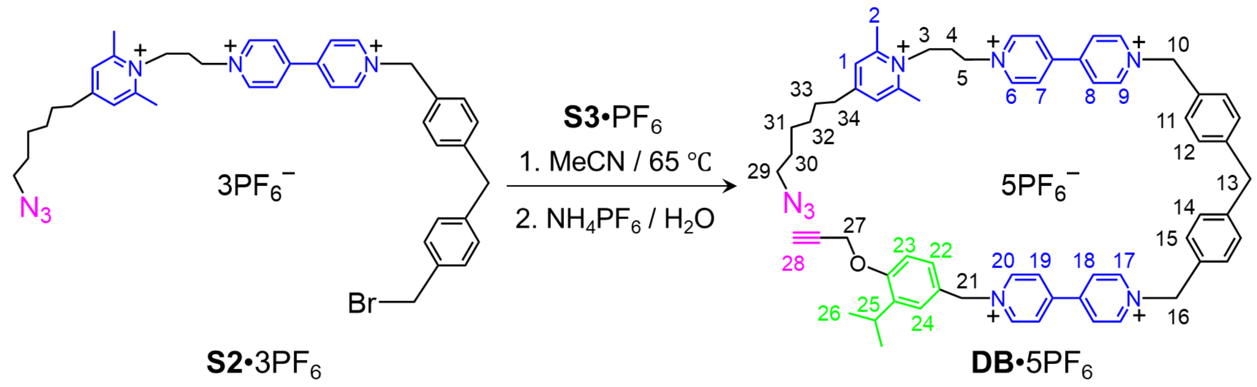

DB•5PF$_6$: **S2•**$3PF_6$ (210 mg, 0.18 mmol) was added into a solution of **S3•**PF_6 (135 mg, 0.28 mmol) in MeCN (10 mL), and the mixture was heated at 65 °C for 72 h. The reaction mixture was cooled to room temperature, and an excess of tetrabutylammonium chloride was added to the solution. The resulting yellow precipitate was filtered off and washed with Me_2CO, Et_2O, and finally purified by column chromatography (SiO_2) using 2% NH_4PF_6 (m/v) Me_2CO solution as the eluent. The fractions containing the desired product were combined, and the solvent was removed under reduced pressure. The residue was washed with H_2O to yield **DB•**$5PF_6$ as a light-yellow solid (195 mg, 63%).

^{1}**H NMR** (500 MHz, CD_3CN) δ 8.96 – 8.91 (m, 8H, $H_{6,9,17,20}$), 8.41 (d, J = 6.5 Hz, 2H, H_8), 8.37 (d, J = 6.5 Hz, 2H, H_7), 8.34 (d, J = 6.5 Hz, 2H, H_{19}), 8.32 (d, J = 6.5 Hz, 2H, H_{18}), 7.58 (s, 2H, H_1), 7.44 – 7.41 (m, 5H, $H_{11,15,24}$), 7.37-7.33 (m, 5H, $H_{12,14,22}$), 7.09 (d, J = 8.5 Hz, 1H, H_{23}), 5.77 (s, 2H, H_{10}), 5.75 (s, 2H, H_{16}), 5.73 (s, 2H, H_{21}), 4.81 (d, J = 2.5 Hz, 2H, H_{27}), 4.80 (t, J = 8.0 Hz, 2H, H_5), 4.49(t, J = 8.5 Hz, 2H, H_3), 4.04 (s, 2H, H_{13}), 3.30 (hept, J = 7.0 Hz, 1H, H_{25}), 3.29 (t, J = 7.0 Hz, 2H, H_{29}), 2.82 (t, J = 2.5 Hz, H, H_{28}), 2.78 (s, 6H, H_2), 2.77(t, 2H, J = 8.0 Hz, H_{34}), 2.52 – 2.46 (m, 2H, H_4), 1.68 (quint, J = 7.5 Hz, 2H, H_{33}), 1.58 (quint, J = 7.0 Hz, 2H, H_{30}), 1.43 – 1.34 (m, 4H, $H_{31,32}$), 1.21 (d, J = 7.0 Hz, 6H, H_{26}). ^{13}**C NMR** (125 MHz, CD_3CN) δ 163.7, 157.0, 155.7, 151.4, 151.3, 151.1, 151.1, 146.8, 146.5, 146.4, 146.2, 144.1, 139.7, 131.5, 131.5, 130.9, 130.6, 130.6, 129.2, 129.0, 128.9, 128.4, 128.3, 126.1, 113.8, 79.5, 76.9, 65.6, 65.4, 65.4, 59.1, 56.8, 52.0, 49.5, 41.5, 35.5, 29.9, 29.7, 29.2, 29.1, 27.7, 26.9, 22.7, 21.3. **HRMS-ESI** (*m/z*): calcd. for $[C_{64}H_{71}F_{30}N_8OP_5 - PF_6]^+$ 1547.4313, found 1547.4298.

Supplementary Scheme 4 | Synthesis of [2]R•9PF6 from DB•5PF6

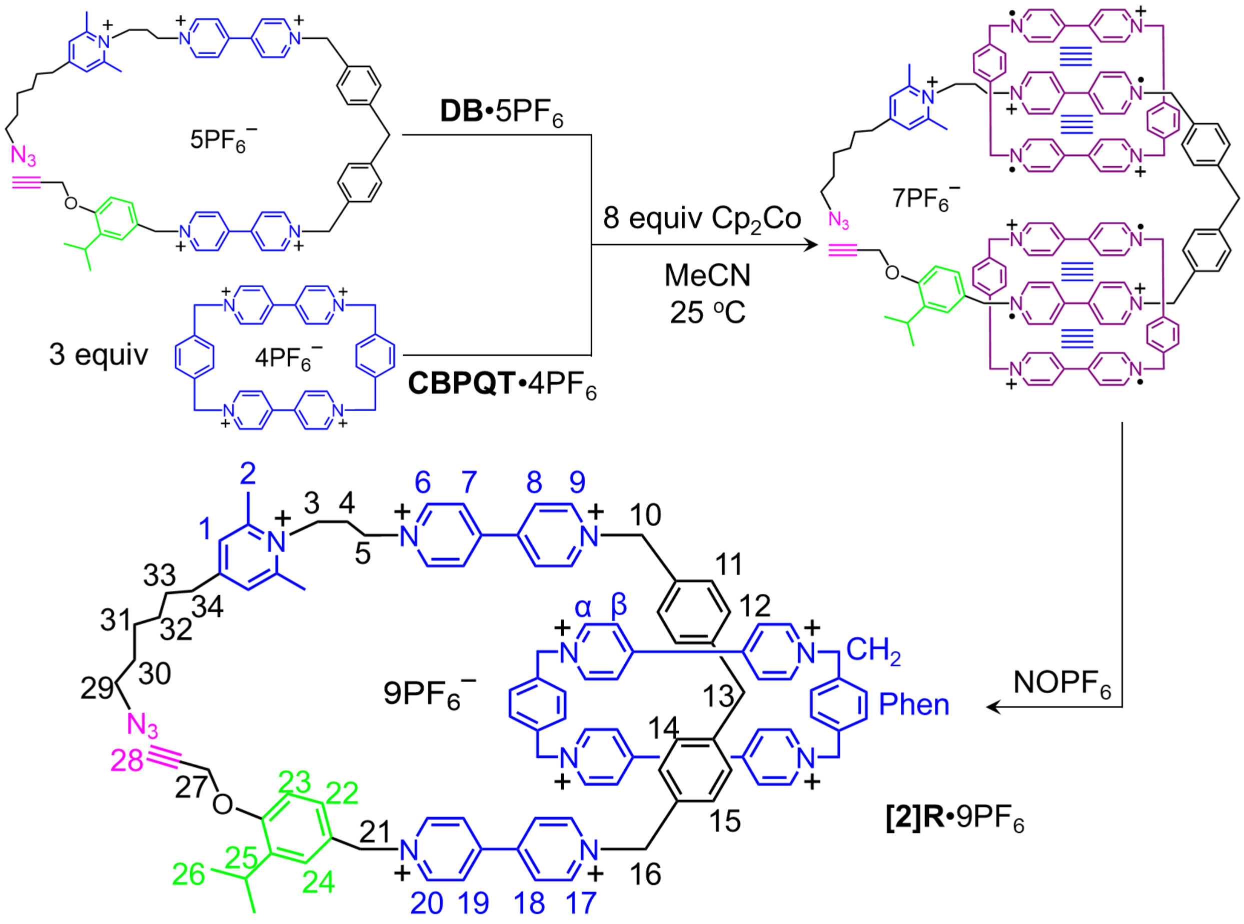

[2]R•9PF6: A solution of cobaltocene (15 mg, 0.08 mmol) in MeCN (2 mL) was added to a solution of **DB**•5PF6 (17 mg, 0.01 mmol) and **CBPQT**•4PF6 (33 mg, 0.03 mmol) dissolved in MeCN (15 mL) in a N_2-filled glovebox. The reaction mixture was stirred at room temperature for 2 h. NOPF6 was added in small aliquots to the resulting dark purple solution until the purple color disappear. The solution was taken out of the glovebox and the solvent was removed by rotary evaporation. The residue was purified using reverse-phase flash chromatography (C18: 0.1% v/v TFA in H_2O and 0.1% v/v TFA in MeCN), the fractions containing the desired product were

combined, and, after an excess of NH_4PF_6 had been added to the solution, a white precipitate was collected by centrifugation and washed with H_2O several times before being dried in vacuo to yield **[2]R**•9PF_6 as an off-white solid (10 mg, 36%).

^{1}H NMR (500 MHz, CD_3CN) δ 9.10 – 9.06 (m, 8H, $H_{6/9/17/20}$), 8.94 (d, *J* = 7.0 Hz, 4H, H_α), 8.71 (d, *J* = 6.5 Hz, 2H), 8.68 (d, *J* = 7.0 Hz, 2H), 8.61 (d, *J* = 7.0 Hz, 2H), 8.53 (d, *J* = 7.0 Hz, 2H), 7.93 (s, 8H, H_{phen}), 7.62 (s, 2H, H_1), 7.58 (d, *J* = 7.0 Hz, 8H, H_β), 7.51 (d, *J* = 2.0 Hz, 1H, H_{24}), 7.41 (dd, *J* = 8.5, 2.0 Hz, 1H, H_{22}), 7.14 (d, *J* = 8.5 Hz, 1H, H_{23}), 6.59 – 6.56 (m, 4H, $H_{11/15}$), 5.88 (s, 2H, H_{10}), 5.86 (s, 2H, H_{16}), 5.84 (s, 8H, H_{CH2}), 5.81 (s, 2H, H_{21}), 4.97 – 4.95 (m, 4H, $H_{12/14}$), 4.88 (t, *J* = 8.0 Hz, 2H, H_5), 4.85 (d, *J* = 2.0 Hz, 1H, H_{27}), 4.55 (t, *J* = 8.5 Hz, 2H, H_3), 3.37 – 3.30 (m, 3H, $H_{25/29}$), 2.85 (t, *J* = 2.5 Hz, 2H, H_{28}), 2.84 (s, 6H, H_2), 2.81(t, *J* = 7.5 Hz, 2H, H_{34}), 2.60 – 2.53 (m, 4H, H_4), 2.26 (s, 2H, H_{13}), 1.72 (p, *J* = 7.5 Hz, 2H, H_{33}), 1.61 (p, *J* = 7.5 Hz, 2H, H_{30}), 1.46 – 1.37 (m, 4H, $H_{31/32}$), 1.26 (d, *J* = 7.0 Hz, 6H, H_{26}). **^{13}C NMR** (125 MHz, CD_3CN) δ 163.7, 157.0, 155.8, 152.1, 151.9, 151.2, 150.8, 147.7, 146.9, 146.5, 146.5, 146.4, 146.0, 140.6, 140.6, 139.7, 138.2, 132.5, 131.9, 130.4, 130.4, 129.4, 129.3, 129.1, 129.0, 129.0, 128.5, 128.5, 128.4, 127.3, 126.1, 113.8, 79.5, 76.9, 65.8, 64.4, 59.2, 56.8, 52.0, 49.5, 41.3, 35.5, 29.9, 29.7, 29.2, 29.1, 27.8, 26.9, 22.7, 21.3. **HRMS-ESI** (*m/z*): calcd. for $[C_{100}H_{103}F_{54}N_{12}OP_9 - PF_6]^+$ 2648.5538, found 2648.5469; calcd. for $[C_{100}H_{103}F_{54}N_{12}OP_9 - 2PF_6]^{2+}$ 1251.7945, found 1251.7938.

Supplementary Scheme 5 | Synthesis of [2]C•9PF_6 from [2]R•9PF_6

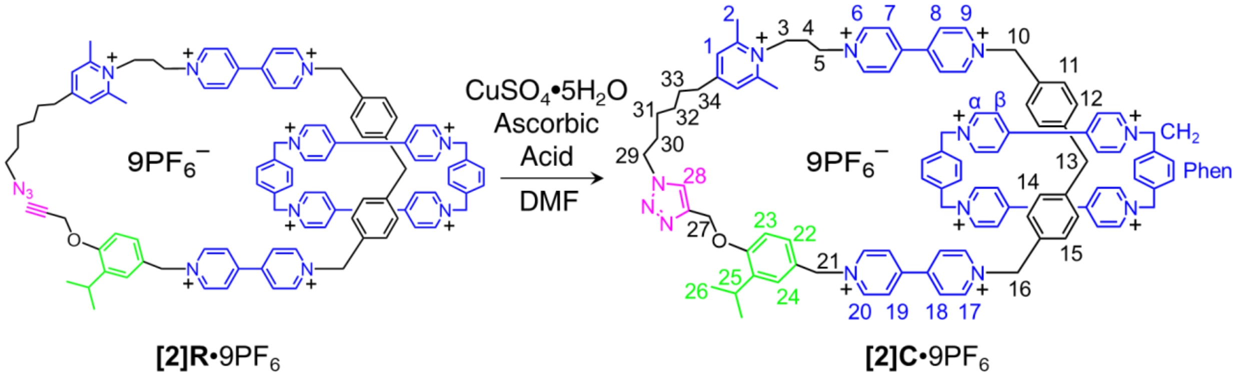

[2]C•9PF₆: **[2]R**•9PF_6 (8 mg, 3 μmol), $CuSO_4$•$5H_2O$ (1 mg), and ascorbic acid (1 mg) were stirred in DMF (1 mL) at room temperature under an atmosphere of N_2 for 1 day. The crude product, obtained after removal of the solvent, was purified using reverse-phase flash chromatography (C18: 0.1% v/v TFA in H_2O and 0.1% v/v TFA in MeCN), the fractions containing the desired product were combined, and, after an excess of NH_4PF_6 had been added to the solution, a white precipitate was collected by centrifugation and washed with H_2O several times before being dried in vacuo to yield **[2]C**•9PF_6 as an off-white solid (2 mg, 25%).

^{1}H NMR (500 MHz, CD_3CN) δ 9.18 (d, J = 6.0 Hz, 4H, $H_{9/17}$), 9.01 (d, J = 6.8 Hz, 4H, $H_{6/20}$), 8.89 (d, J = 6.9 Hz, 8H, H_α), 8.71 (d, J = 5.9 Hz, 4H, $H_{8/19}$), 8.57 (d, J = 6.9 Hz, 2H, H_7), 8.51 (d, J = 6.9 Hz, 2H, H_{19}), 7.82 (s, 1H, H_{28}), 7.78 (s, 8H, H_{phen}), 7.53 (d, J = 6.8 Hz, 8H, H_β), 7.52 (s, 2H, H_1), 7.44 (d, J = 2.2 Hz, 1H, H_{24}), 7.31 (dd, J = 8.4, 2.3 Hz, 1H, H_{22}), 7.14 (d, J = 8.5 Hz, 1H, H_{23}), 6.40 (d, J = 8.0 Hz, 2H, H_{11}), 6.27 (d, J = 8.0 Hz, 2H, H_{15}), 5.88 (s, 2H, H_{10}), 5.86 (s, 2H, H_{16}), 5.77 (s, 8H, H_{CH2}), 5.76 (s, 2H, H_{21}), 5.10 (s, 2H, H_{27}), 5.08 (d, J = 8.0 Hz, 2H, H_{12}), 4.89 (d, J = 8.0 Hz, 2H, H_{14}), 4.83 (t, J = 8.0 Hz, 2H, H_5), 4.48 (t, J = 8.5 Hz, 2H, H_3), 4.33 (t, J = 6.9 Hz, 2H, H_{29}), 3.20 (p, J = 7.0 Hz, 1H, H_{25}), 2.75 (s, 6H, H_2), 2.74 – 2.69 (m, 2H, H_{34}), 2.50 – 2.43 (m,

4H, $H_{4/13}$), 1.87 – 1.82 (m, 2H, H_{30}), 1.61 (p, J = 7.5 Hz, 2H, H_{33}), 1.34 (q, J = 7.5 Hz, 2H, H_{32}), 1.27 (m, 2H, H_{31}), 1.14 (d, J = 6.9 Hz, 6H, H_{26}). **^{13}C NMR** (125 MHz, CD_3CN) δ 163.6, 158.0, 155.7, 152.1, 152.1, 152.0, 151.0, 150.5, 147.6, 146.9, 146.9, 146.8, 146.3, 145.9, 144.0, 140.2, 139.4, 138.2, 133.2, 133.1, 131.8, 131.3, 129.6, 129.4, 129.4, 129.4, 129.3, 129.2, 129.1, 129.0, 128.8, 128.6, 128.5, 127.2, 126.0, 124.7, 113.7, 79.1, 65.6, 64.3, 64.2, 63.0, 50.8, 49.4, 41.1, 35.4, 30.4, 29.8, 29.8, 28.8, 27.9, 26.6, 22.5, 21.3.

Supplementary Scheme 6 | Synthesis of [3]CMM•$13PF_6$ **from DB•**$5PF_6$

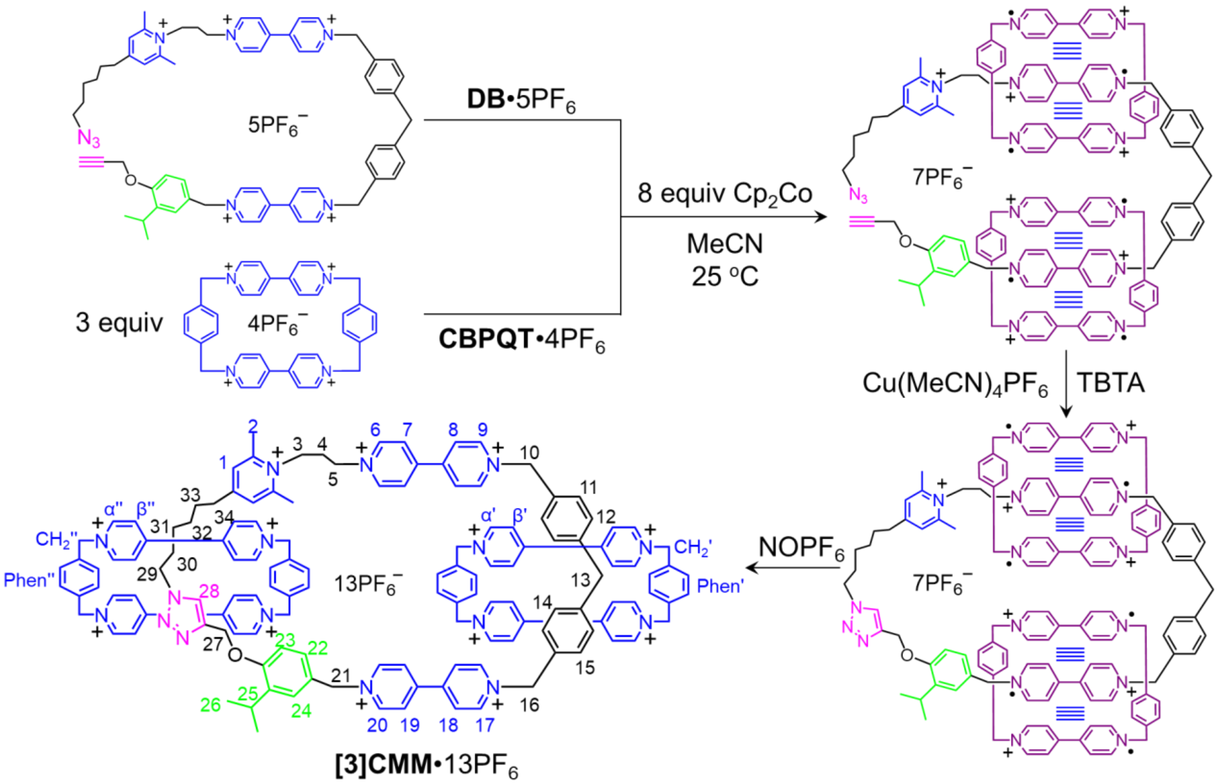

[3]CMM•13CF₃COO: A solution of cobaltocene (15 mg, 0.08 mmol) in MeCN (2 mL) was added to a solution of **DB•**$5PF_6$ (17 mg, 0.01 mmol) and **CBPQT•**$4PF_6$ (33 mg, 0.03 mmol) dissolved in MeCN (15 mL) in a N_2-filled glovebox. The reaction mixture was stirred at room temperature for 2 h before adding $Cu(MeCN)_4PF_6$ (4 mg, 0.01 mmol) and tris[(1-benzyl-1H-1,2,3-triazol-4-

yl)methyl]amine (TBTA) (5 mg, 0.01 mmol) into this solution. After reacting for 4 days, $NOPF_6$ was added in small aliquots to the resulting dark purple solution until the purple color disappeared. The solution was taken out of the glovebox and the solvent was removed by rotary evaporation. The residue was purified using reverse-phase flash chromatography (C18: 0.1% v/v TFA in H_2O and 0.1% v/v TFA in MeCN), the fractions containing the desired product were combined, and the solvent was removed under reduced pressure to yield **[3]CMM**•13CF_3COO as an off-white solid. **^{1}H NMR** (600 MHz, D_2O) δ 9.51 (d, J = 7.2 Hz, 2H, H_9), 9.42 (d, J = 7.2 Hz, 2H, H_{20}), 9.39 (d, J = 7.2 Hz, 2H, H_{17}), 9.31 (d, J = 7.2 Hz, 2H, H_6), 9.20 (d, J = 7.2 Hz, 8H, $H_{\alpha'}$), 9.16 (d, J = 7.2 Hz, 8H, $H_{\alpha''}$), 8.96 (d, J = 7.2 Hz, 2H, H_8), 8.89 (d, J = 7.2 Hz, 2H, H_{18}), 8.81 (d, J = 7.2 Hz, 2H, H_{19}), 8.74 (d, J = 6.5 Hz, 2H, H_7), 8.04 (d, J = 7.2 Hz, 8H, $H_{\beta''}$), 7.82 (d, J = 7.2 Hz, 8H, $H_{\beta'}$), 7.75 (s, 11H, $H_{1,24,Phen'}$), 7.73 (s, 8H, $H_{Phen''}$), 7.55 (dd, J = 8.4, 2.0 Hz, 1H, H_{22}), 6.65 (d, J = 7.8 Hz, 2H, H_{11}), 6.59 (d, J = 8.4 Hz, 1H, H_{23}), 6.28 (d, J = 7.8 Hz, 2H, H_{15}), 6.09 (s, 2H, H_{10}), 6.03 (s, 2H, H_{21}), 6.00 (s, 2H, H_{16}), 5.87 – 5.81 (m, 16H, $H_{CH2', CH2''}$), 5.17 (d, J = 8.4 Hz, 2H, H_{12}), 5.05(t, J = 8.4 Hz, 2H, H_5), 4.76 (2H, H_{14}), 4.73 (2H, H_3), 4.46 (s, 1H, H_{28}), 3.37(t, J = 7.8 Hz, 2H, H_{34}), 3.07 (s, 2H, H_{27}), 3.01 (quint, J = 7.2 Hz, 2H, H_{25}), 2.96(t, J = 7.2 Hz, 2H, H_{29}), 2.91 (s, 6H, H_2), 2.73 – 2.67 (m, 2H, H_4), 2.32 (s, 2H, H_{13}), 1.85 (quint, J = 7.8 Hz, 2H, H_{30}), 1.38 (quint, J = 7.8 Hz, 2H, H_{31}), 1.33 (d, J = 7.2 Hz, 6H, H_{26}), 1.26 (quint, J = 7.8 Hz, 2H, H_{32}), 1.15 (quint, J = 7.8 Hz, 2H, H_{33}). **^{13}C NMR** (125 MHz, D_2O) δ 162.6 (q, $J_{C\text{-}F}$ = 35 Hz, CF_3COO), 162.4, 155.7, 154.6, 151.1, 150.9, 149.8, 149.4, 147.4, 146.9, 146.2, 146.1, 145.9, 145.6, 145.0, 145.0, 140.4, 139.4, 138.7, 138.6, 137.2, 136.4, 132.6, 132.0, 130.5, 130.4, 128.6, 128.4, 128.2, 128.1, 128.0, 128.0,

127.9, 127.9, 127.5, 127.4, 126.3, 126.2, 120.4, 116.3 (q, $J_{C\text{-}F}$ = 292 Hz, CF_3COO), 64.8, 64.5, 63.5, 63.2, 61.8, 49.8, 48.4, 39.9, 34.5, 29.5, 28.9, 28.2, 27.8, 26.4, 26.3, 22.5, 20.3.

[3]CMM•13PF$_6$: After anion exchange from CF_3COO^- to PF_6^- by adding an excess of NH_4PF_6 into an aqueous solution of **[3]CMM**•13CF_3COO, a white precipitate was collected by centrifugation and washed with H_2O several times before being dried in vacuo to yield **[3]CMM**•13PF_6 as an off-white solid (16 mg, 40%).

^{1}H NMR (500 MHz, CD_3CN) δ 9.12 (d, J = 5.5 Hz, 2H, H_9), 9.09 (d, J = 5.5 Hz, 2H, H_{20}), 9.04 – 9.03 (m, 4H, $H_{6,17}$), 8.81 – 8.78 (m, 16H, $H_{\alpha',\alpha''}$), 8.70 (d, J = 5.5 Hz, 2H, H_8), 8.68 (d, J = 6.0 Hz, 2H, H_{18}), 8.58 (d, J = 6.0 Hz, 2H, H_{19}), 8.56 (d, J = 5.5 Hz, 2H, H_7), 7.78 (d, J = 5.5 Hz, 8H, $H_{\beta''}$), 7.77 (s, 8H, $H_{phen'}$), 7.68 (s, 2H, H_1), 7.64 (s, 9H, $H_{24,phen''}$), 7.46 (d, J = 5.5 Hz, 8H, $H_{\beta'}$), 7.41 (d, J = 8.5 Hz, 1H, H_{22}), 6.59 (d, J = 8.5 Hz, 1H, H_{23}), 6.51 (d, J = 7.5 Hz, 2H, H_{11}), 6.15 (d, J = 7.5 Hz, 2H, H_{15}), 5.89 (s, 4H, $H_{10,21}$), 5.81 (s, 2H, H_{16}), 5.75 – 5.66 (m, 16H, $H_{CH2', CH2''}$), 5.10 (d, J = 7.5 Hz, 2H, H_{12}), 4.86 (t, J = 7.5 Hz, 2H, H_5), 4.70 (m, 3H, $H_{14,28}$), 4.54 (t, J = 7.5 Hz, 2H, H_3), 3.37 (t, J = 8.0 Hz, 2H, H_{34}), 3.01 (quint, J = 7.0 Hz, 1H, H_{25}), 2.95 (s, 2H, H_{27}), 2.91 (t, J = 7.5 Hz, 2H, H_{29}), 2.82 (s, 6H, H_2), 2.57 – 2.51 (m, 2H, H_4), 2.32 (s, 2H, H_{13}), 1.78 (quint, J = 7.0 Hz, 2H, H_{30}), 1.43 (quint, J = 7.0 Hz, 2H, H_{31}), 1.36 (d, J = 7.0 Hz, 6H, H_{26}), 1.32 – 1.27 (m, 2H, H_{32}), 1.21 – 1.15 (m, 2H, H_{33}). **^{13}C NMR** (125 MHz, CD_3CN) δ 163.5, 157.6, 155.8, 152.3, 152.2, 151.0, 150.8, 148.2, 147.7, 146.8, 146.3, 146.0, 145.9, 145.8, 141.0, 140.2, 140.1, 140.0, 138.2, 137.2, 133.5, 132.9, 131.8, 131.4, 129.7, 129.7, 129.4, 129.3, 129.0, 128.9, 128.6, 128.5, 127.4, 127.2,

122.4, 117.2, 65.6, 64.2, 63.4, 59.2, 56.9, 50.6, 49.5, 41.2, 35.7, 30.8, 30.3, 29.6, 28.8, 27.6, 27.3, 23.3, 21.3.

^{1}H NMR (500 MHz, CD_3COCD_3) δ 9.56 (d, *J* = 6.5 Hz, 2H, H_9), 9.52 – 9.47 (m, 4H, $H_{20,17}$), 9.48 (d, *J* = 6.5 Hz, 2H, H_6), 9.28 (d, *J* = 6.5 Hz, 16H, $H_{\alpha',\alpha''}$), 8.99 (d, *J* = 6.5 Hz, 4H, $H_{8,18}$), 8.88 (d, *J* = 6.5 Hz, 2H, H_{19}), 8.85 (d, *J* = 6.5 Hz, 2H, H_7), 8.26 (d, *J* = 7.0 Hz, 8H, $H_{\beta''}$), 8.08 (d, *J* = 6.5 Hz, 8H, $H_{\beta'}$), 7.99 (s, 8H, $H_{phen'}$), 7.95 (s, 8H, $H_{phen''}$), 7.88 (s, 2H, H_1), 7.86 (d, *J* = 2.5 Hz, 1H, H_{24}), 7.66 (dd, *J* = 8.5, 2.0 Hz, 1H, H_{22}), 6.80 (d, *J* = 8.0 Hz, 2H, H_{11}), 6.63 (d, *J* = 8.5 Hz, 1H, H_{23}), 6.25 (s, 2H, H_{10}), 6.24 (s, 2H, H_{21}), 6.14 – 6.13 (m, 4H, $H_{15,16}$), 6.06 – 5.95 (m, 16H, $H_{CH2', CH2''}$), 5.64 (d, *J* = 8.0 Hz, 2H, H_{12}), 5.40 – 5.37 (m, 3H, $H_{5,28}$), 5.00 – 4.96 (m, 4H, $H_{3,14}$), 3.77 – 3.74 (m, 2H, H_{34}), 3.24 – 3.18 (m, 1H, H_{25}), 3.05 (s, 2H, H_{27}), 3.02 (s, 6H, H_2), 3.01 – 2.96 (m, 2H, H_4), 2.94 (t, *J* = 7.0 Hz, 2H, H_{29}), 2.84 (s, 2H, H_{13}), 1.81 (quint, *J* = 7.5 Hz, 2H, H_{30}), 1.50 – 1.43 (m, 4H, $H_{31,32}$), 1.40 (d, *J* = 6.5 Hz, 6H, H_{26}), 1.38 – 1.33 (m, 2H, H_{33}). **^{13}C NMR** (125 MHz, CD_3COCD_3) δ 163.3, 157.6, 156.0, 152.6, 152.6, 151.1, 151.0, 148.5, 148.0, 147.3, 147.2, 146.6, 146.2, 146.1, 141.1, 140.8, 140.3, 140.1, 138.5, 137.6, 134.2, 133.3, 131.8, 131.6, 130.1, 129.5, 129.5, 129.5, 129.3, 129.1, 128.9, 128.8, 128.7, 128.5, 127.9, 127.5, 127.4, 122.7, 117.2, 65.7, 65.7, 64.5, 64.1, 63.5, 59.6, 50.7, 49.8, 41.3, 35.5, 30.6, 30.4, 29.2, 27.5, 27.3, 23.5, 21.1.

HRMS-ESI (*m/z*): calcd. for $[C_{136}H_{135}F_{78}N_{16}OP_{13} - 2PF_6]^{2+}$ 1801.8544, found 1801.8542; calcd. for $[C_{136}H_{135}F_{78}N_{16}OP_{13} - 3PF_6]^{3+}$ 1152.9147, found 1152.9143.

3. NMR Spectroscopy

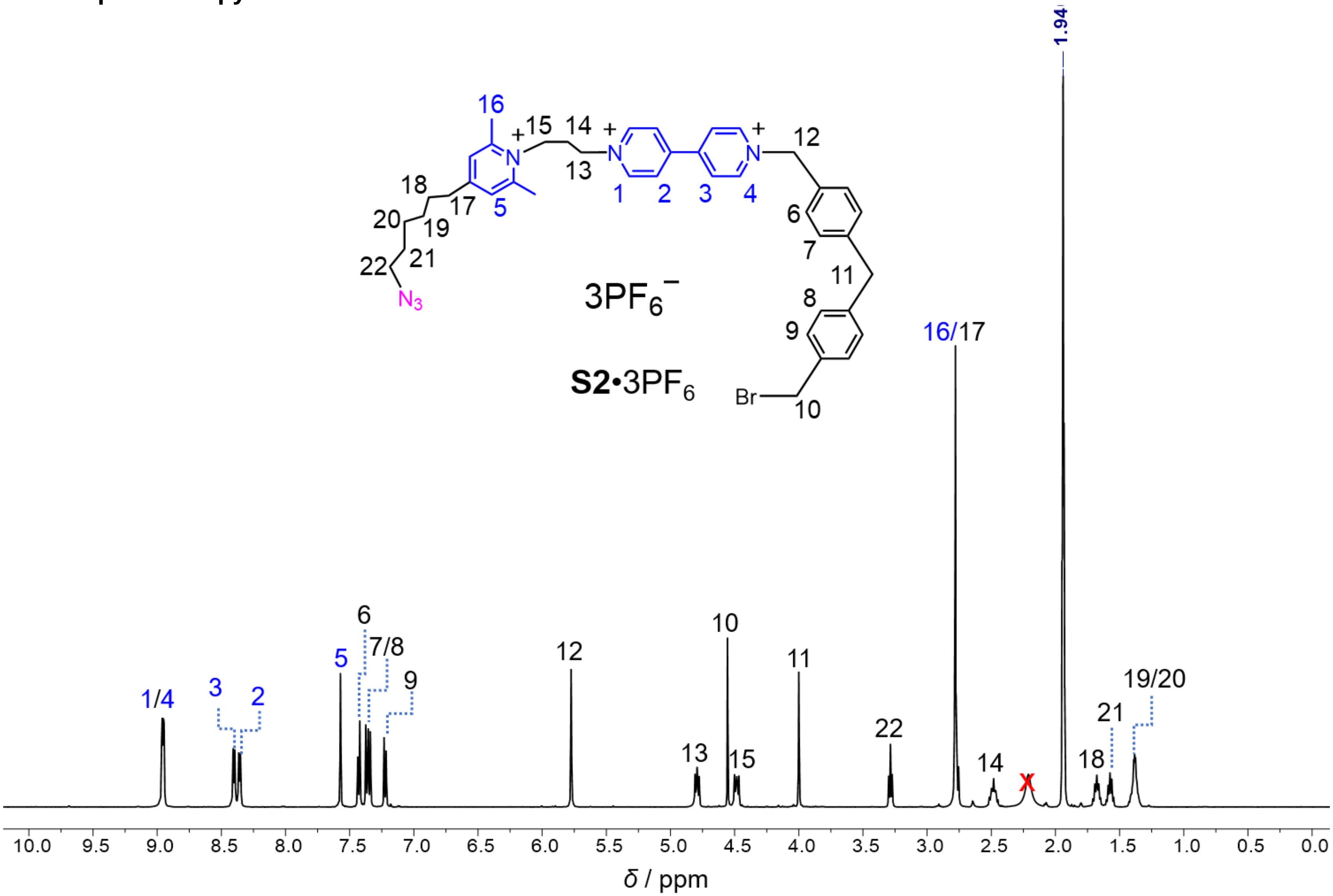

Supplementary Fig. 1 | ^{1}H NMR Spectrum (500 MHz, CD_3CN, 298 K) of **S2**•$3PF_6$

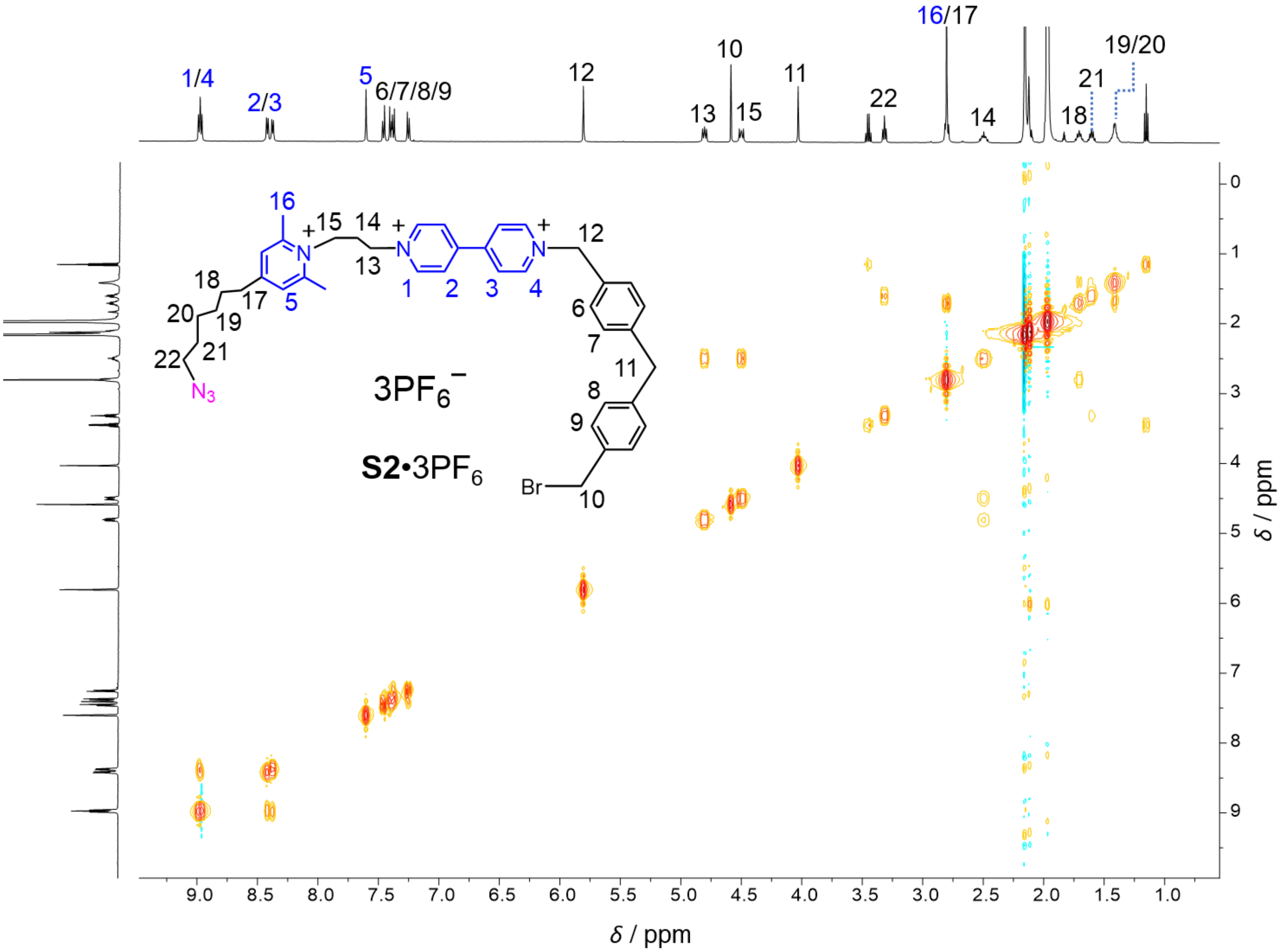

Supplementary Fig. 2 | ^1H-^1H COSY Spectrum (500 MHz, CD_3CN, 298 K) of **S2**•3PF_6

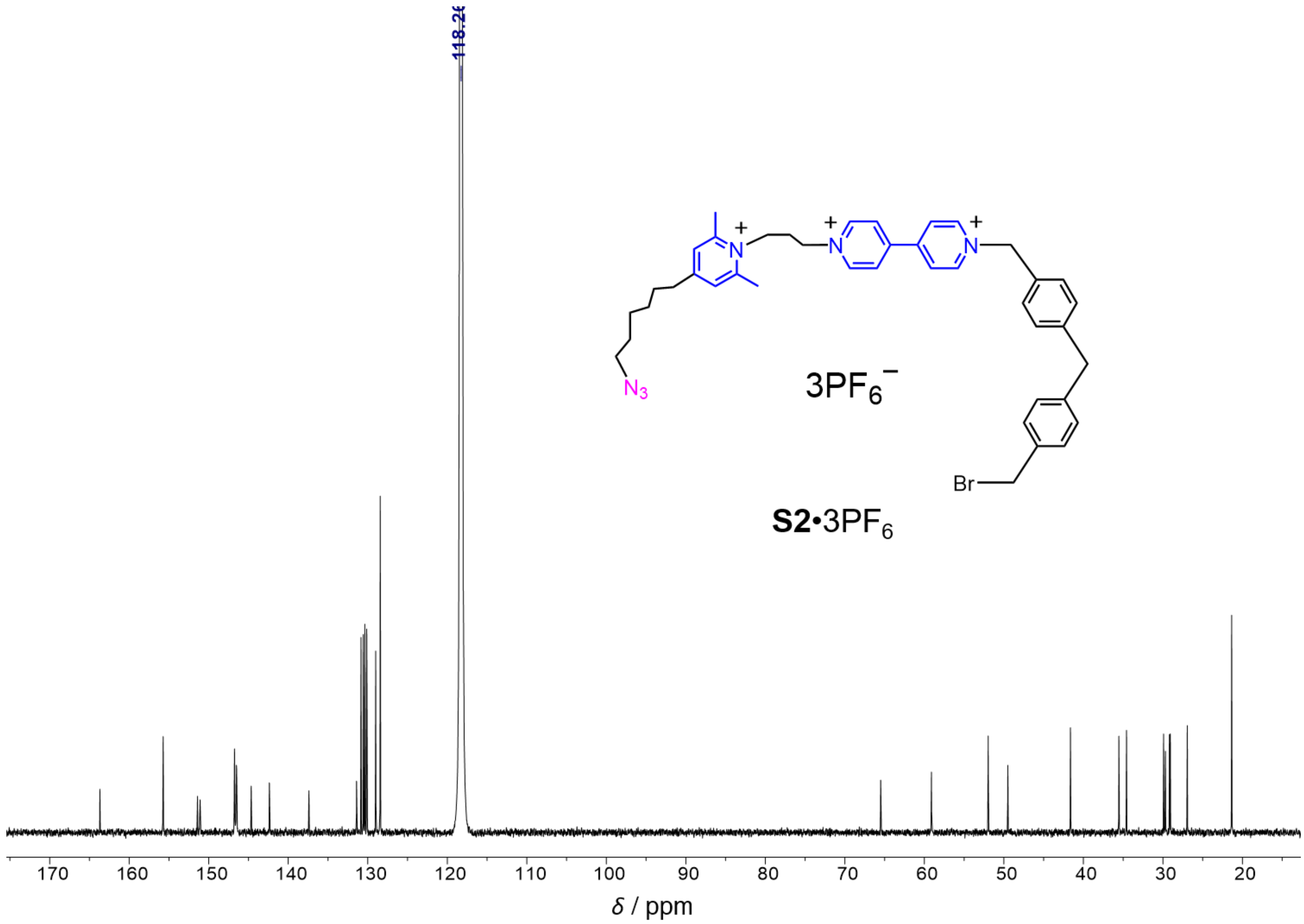

Supplementary Fig. 3 | ^{13}C NMR Spectrum (125 MHz, CD_3CN, 298 K) of **S2**•$3PF_6$

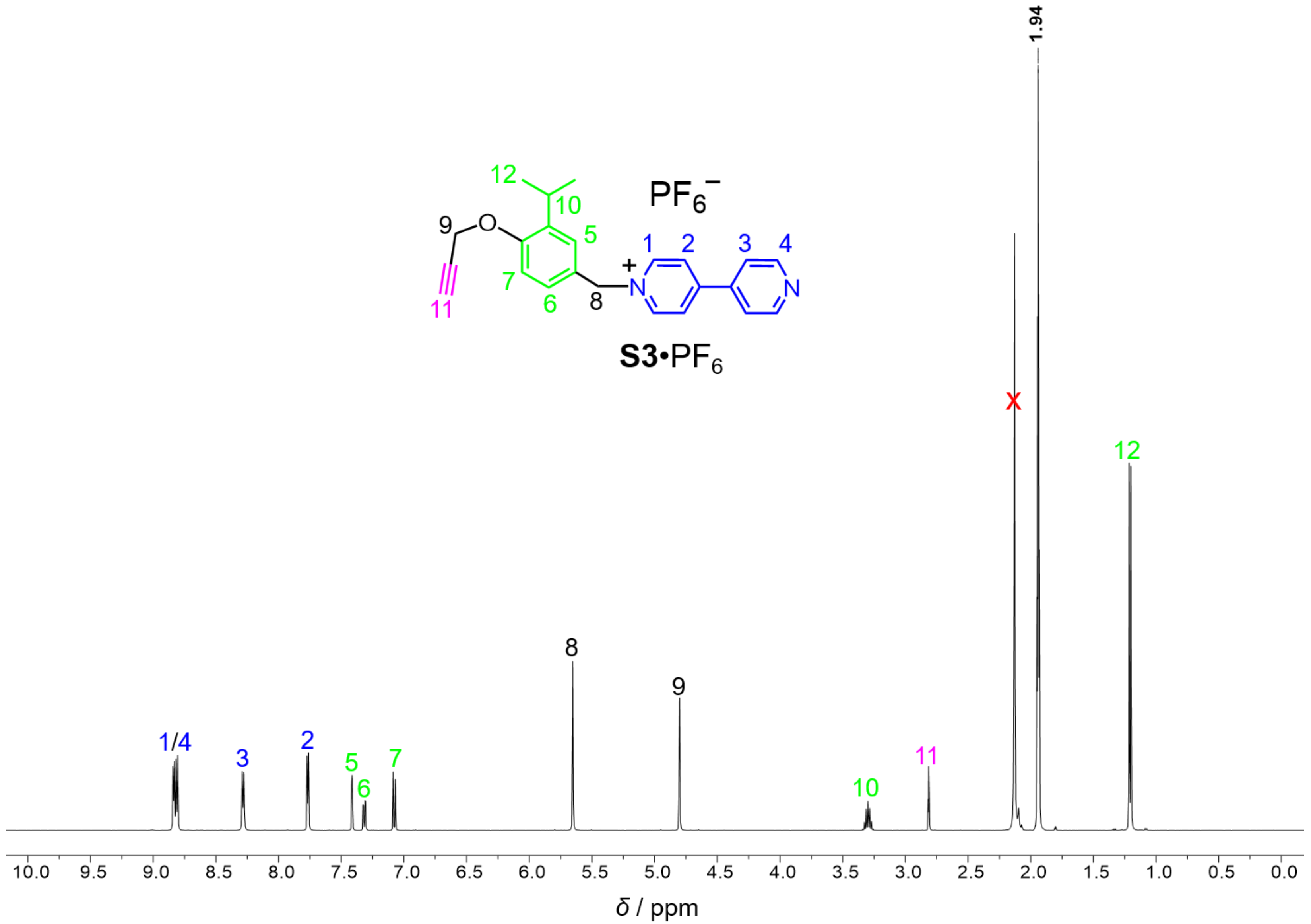

Supplementary Fig. 4 | ^{1}H NMR Spectrum (500 MHz, CD_3CN, 298 K) of **S3**•PF_6

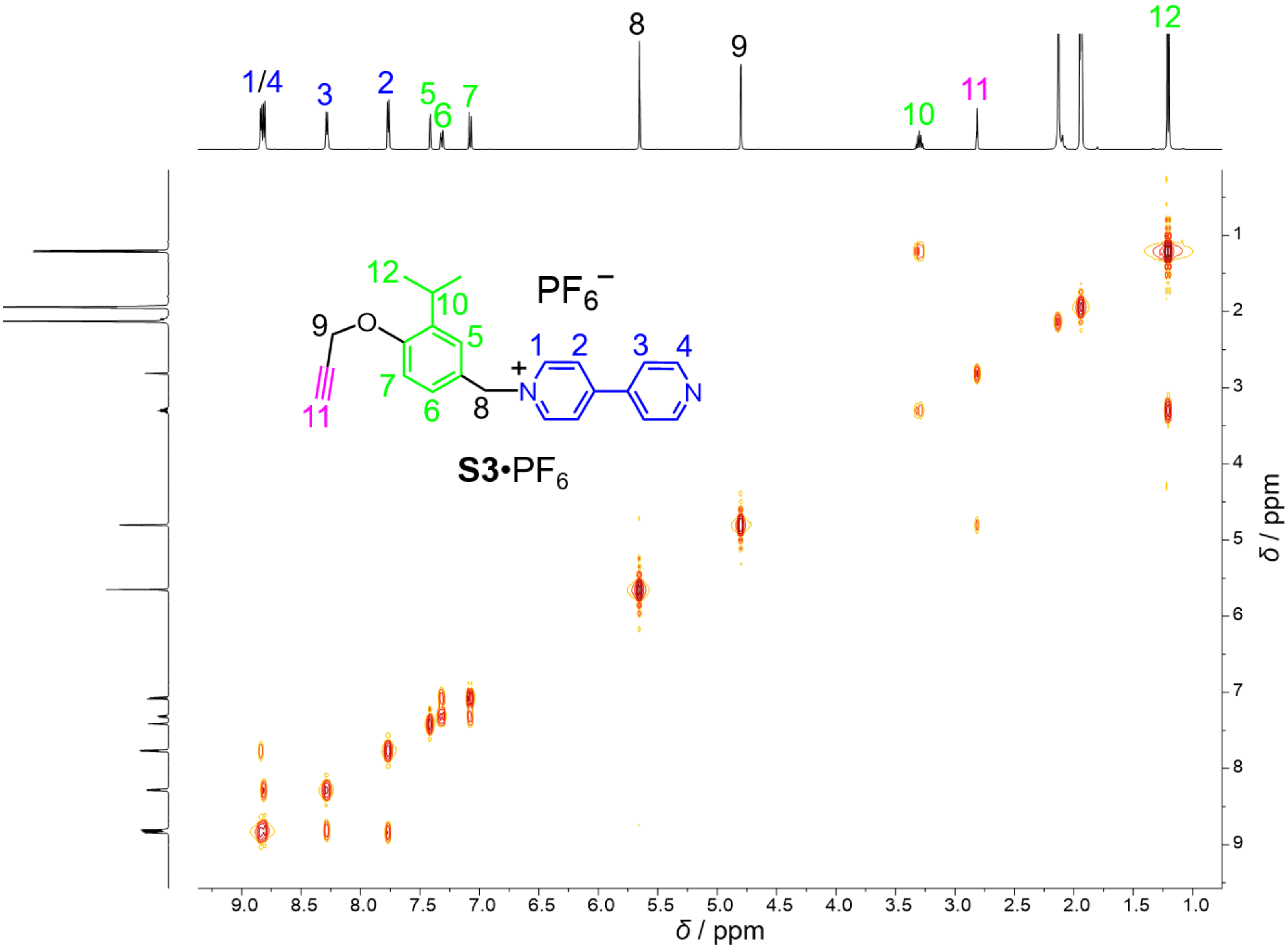

Supplementary Fig. 5 | ^{1}H-^{1}H COSY Spectrum (500 MHz, CD_3CN, 298 K) of **S3**•PF_6

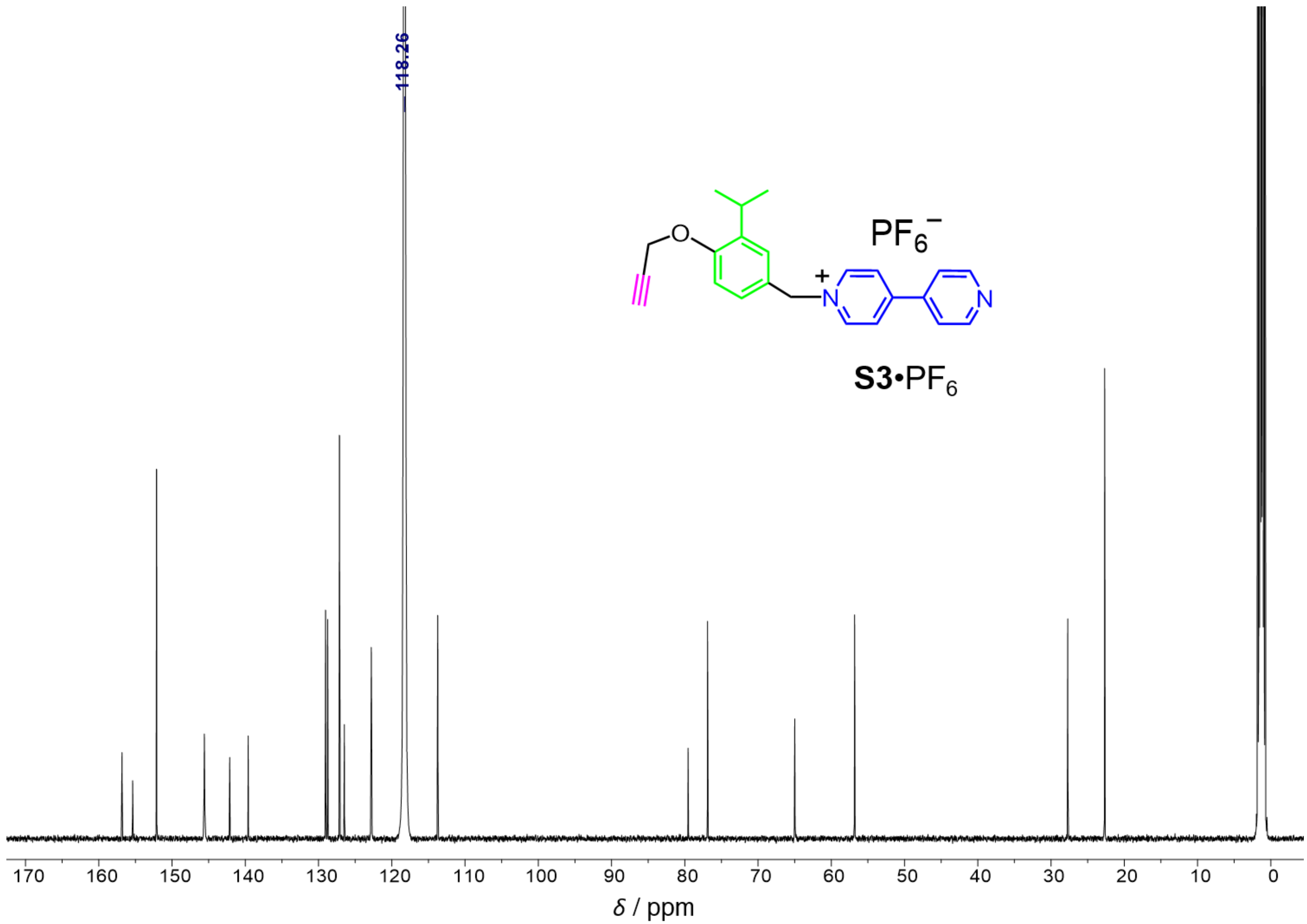

Supplementary Fig. 6 | ^{13}C NMR Spectrum (125 MHz, CD_3CN, 298 K) of **S3**•PF_6

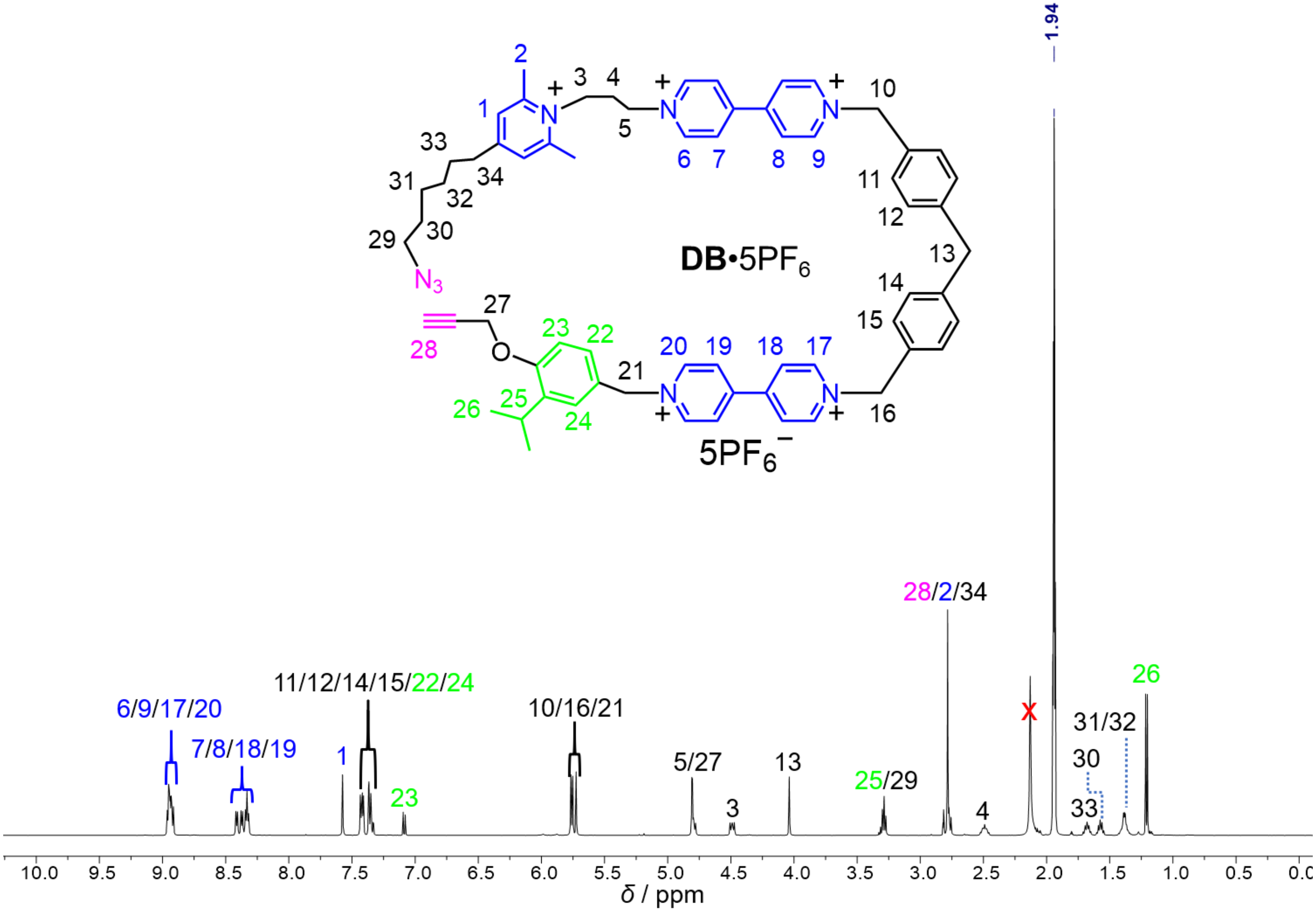

Supplementary Fig. 7 | ^{1}H NMR Spectrum (500 MHz, CD_3CN, 298 K) of **DB**•$5PF_6$

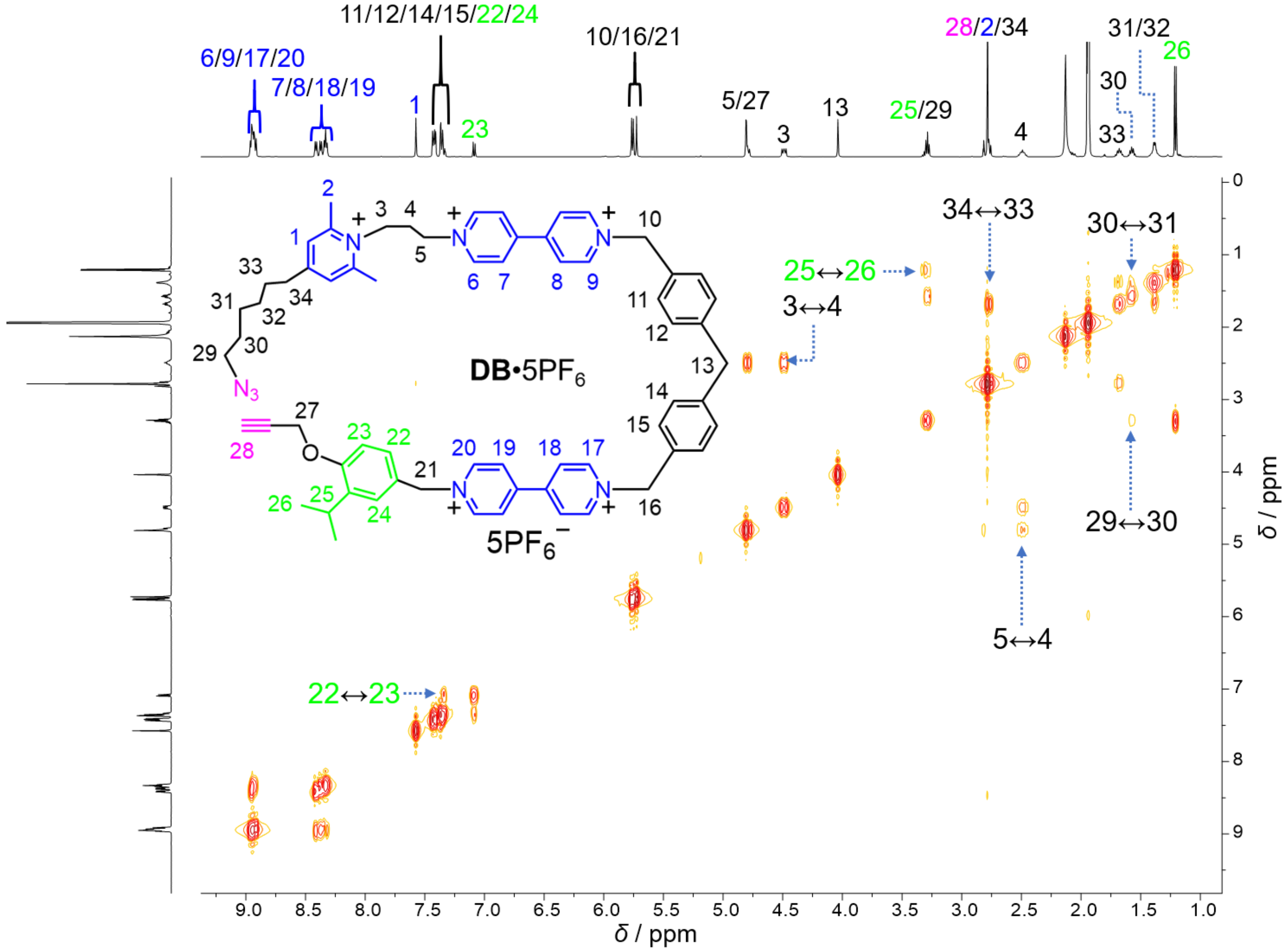

Supplementary Fig. 8 | ¹H-¹H COSY Spectrum (500 MHz, CD_3CN, 298 K) of **DB**•$5PF_6$

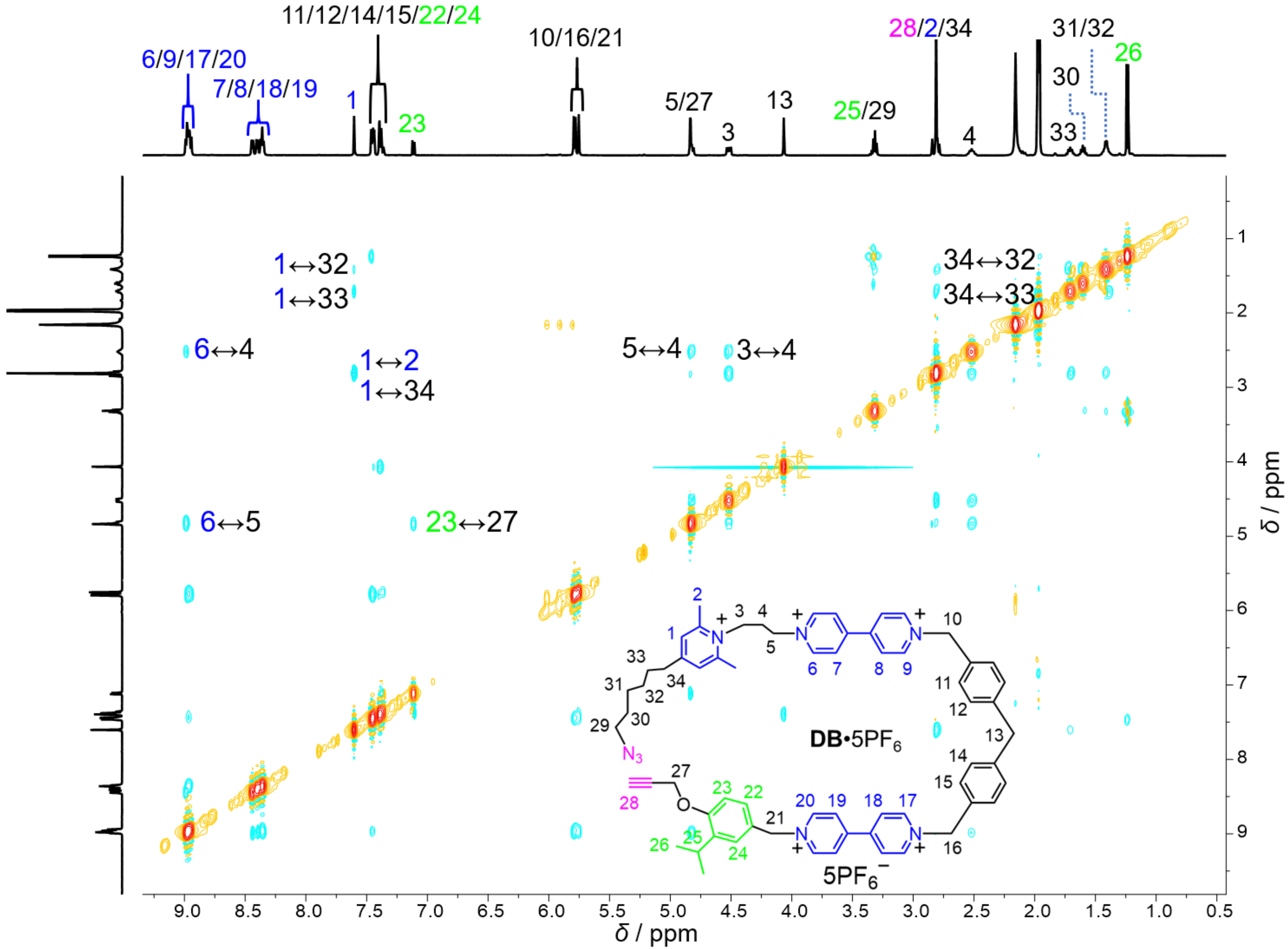

Supplementary Fig. 9 | ^{1}H-^{1}H NOESY Spectrum (500 MHz, CD_3CN, 298 K) of **DB•**$5PF_6$

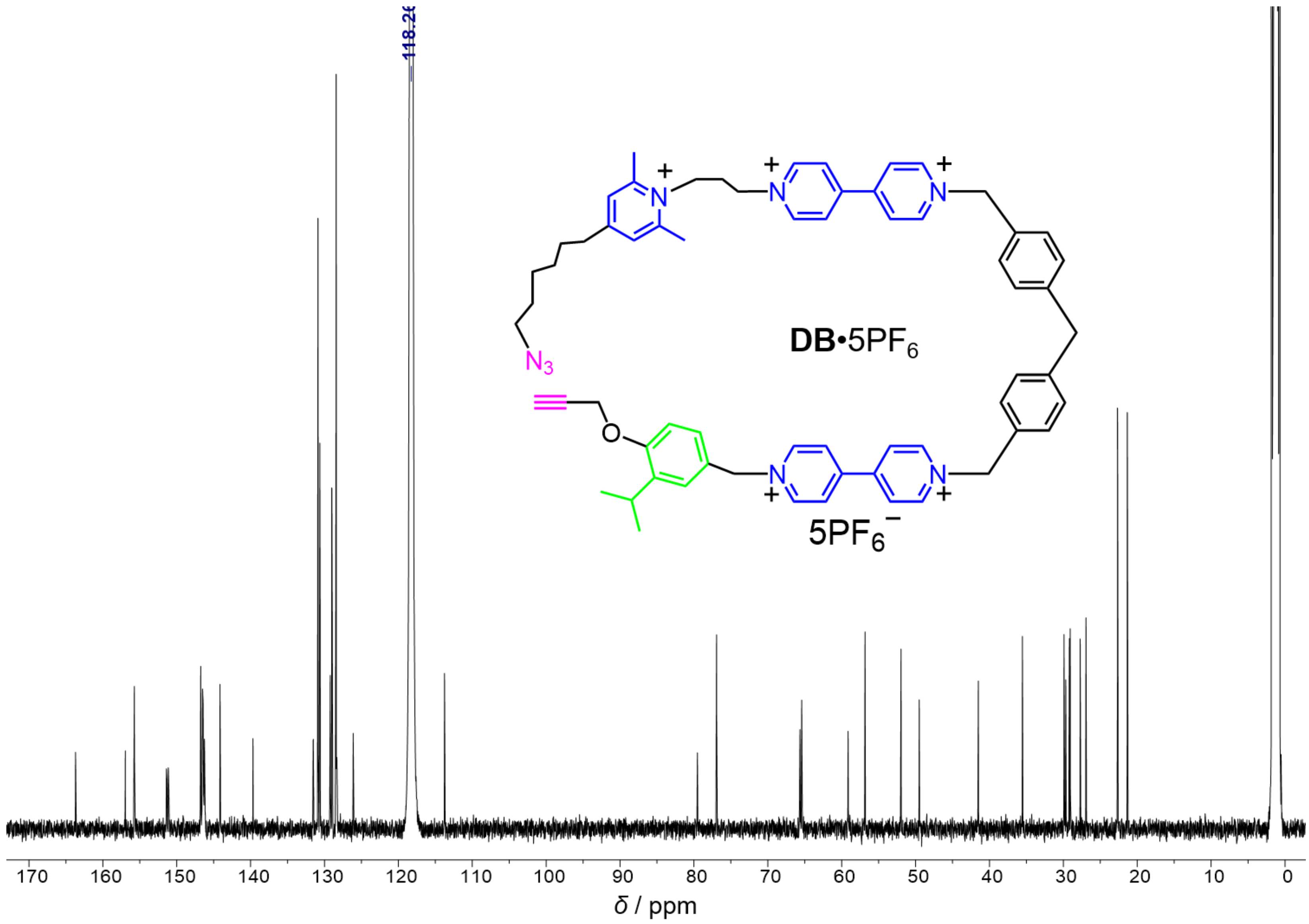

Supplementary Fig. 10 | ^{13}C NMR Spectrum (125 MHz, CD_3CN, 298 K) of **DB**•$5PF_6$

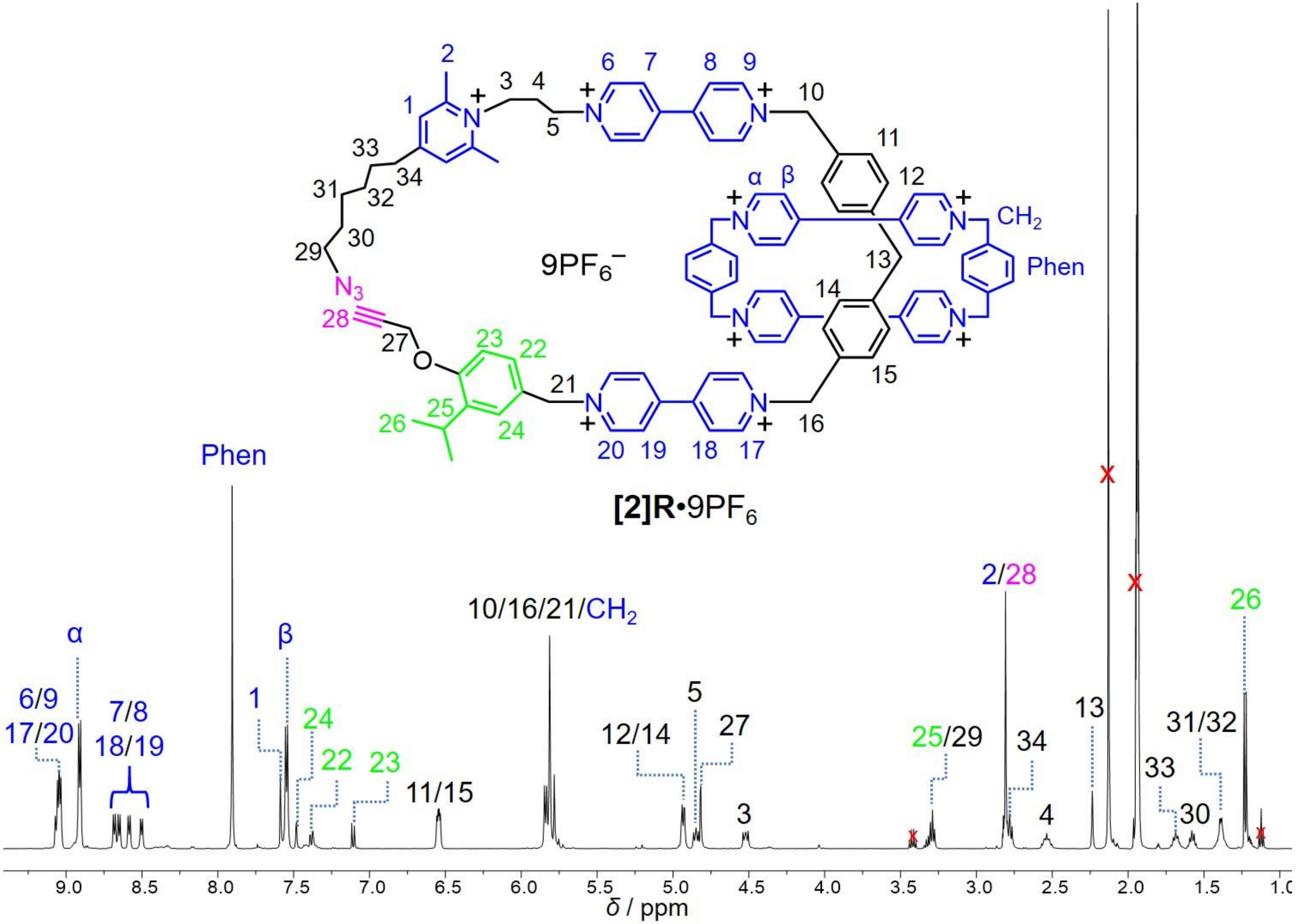

Supplementary Fig. 11 | ^{1}H NMR Spectrum (500 MHz, CD_3CN, 298 K) of **[2]R**•$9PF_6$

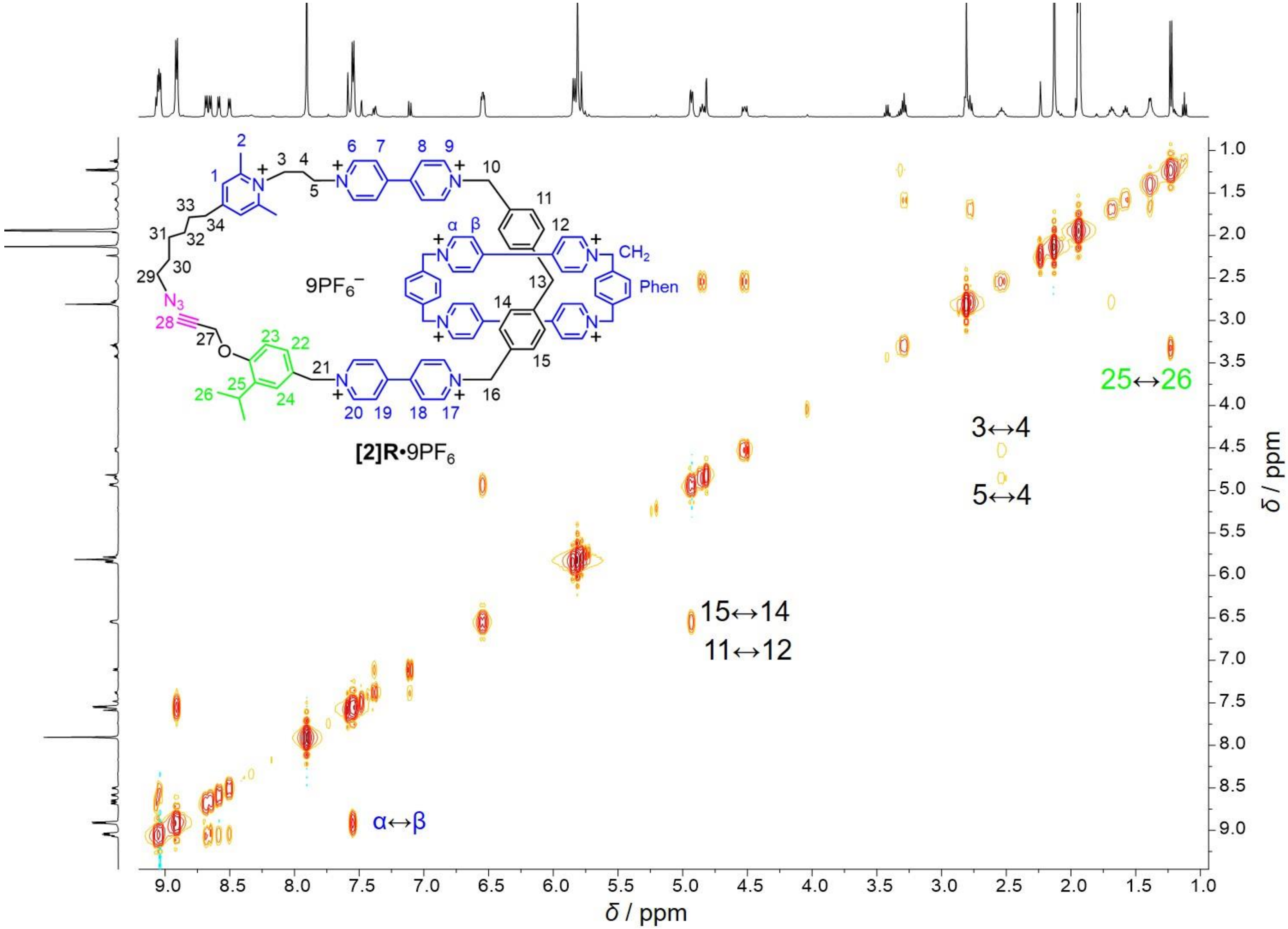

Supplementary Fig. 12 | ^{1}H-^{1}H COSY Spectrum (500 MHz, CD_3CN, 298 K) of **[2]R**•$9PF_6$

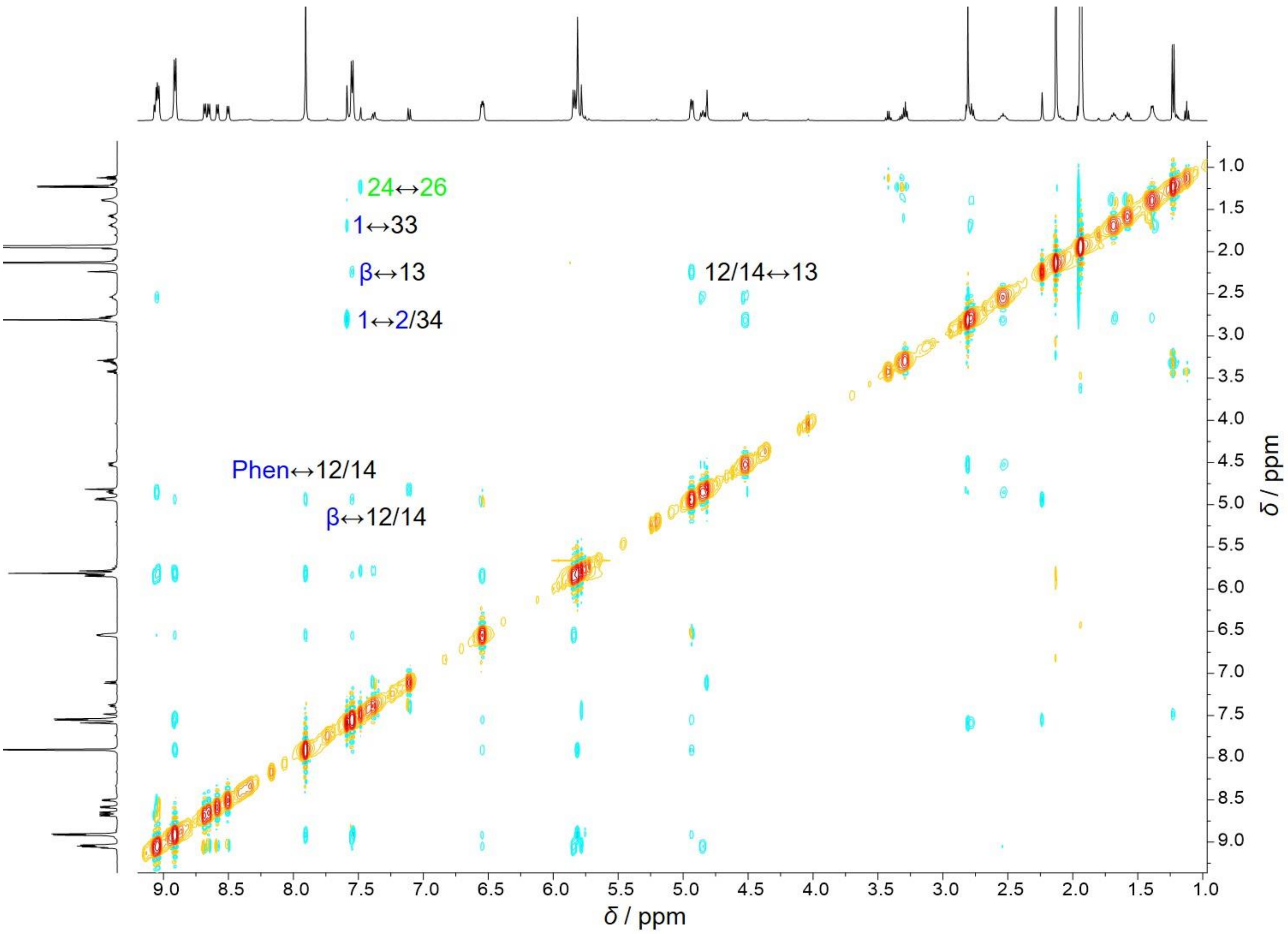

Supplementary Fig. 13 | ^{1}H-^{1}H NOESY Spectrum (500 MHz, CD_3CN, 298 K) of **[2]R**•$9PF_6$

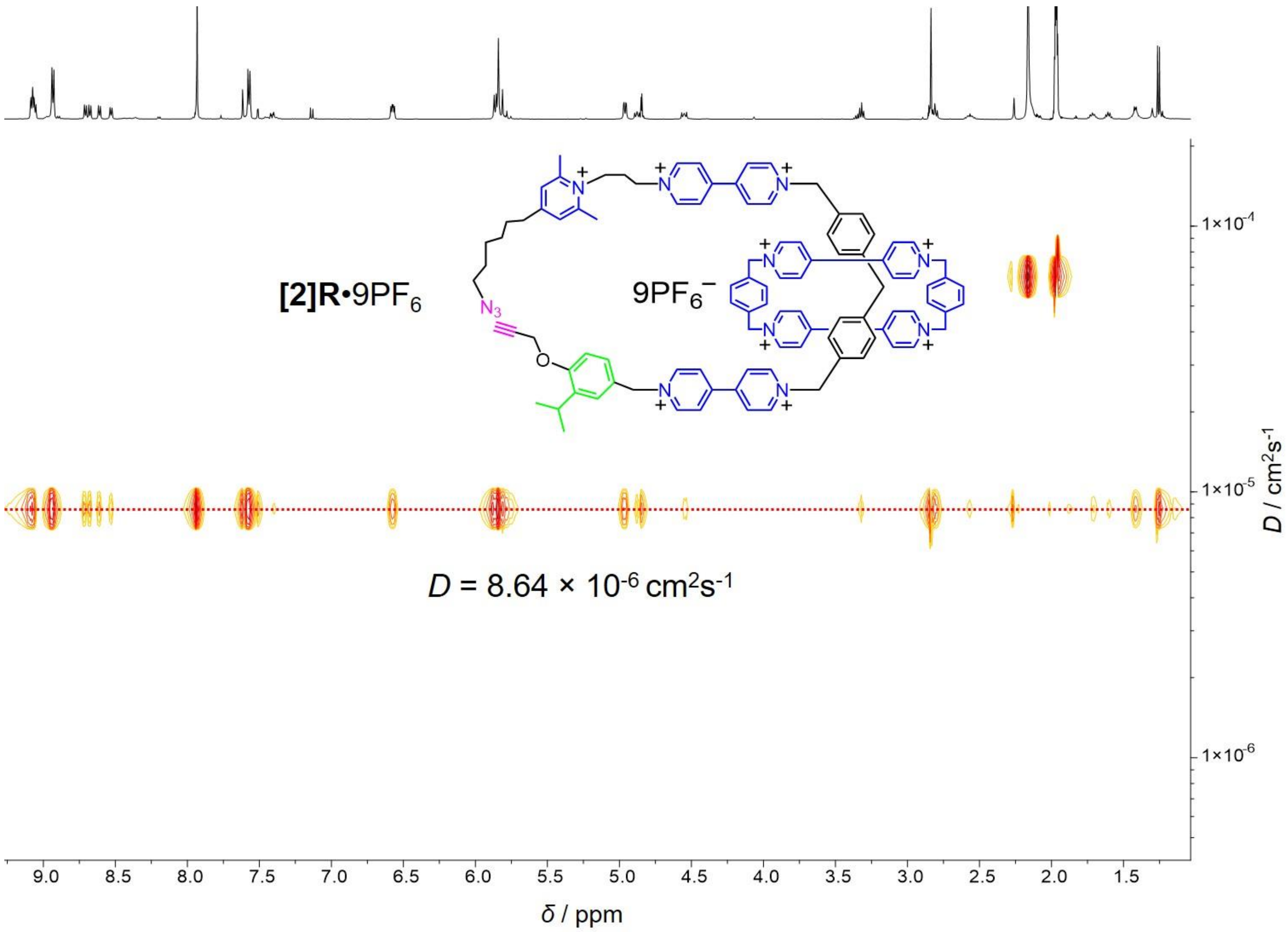

Supplementary Fig. 14 | ^{1}H DOSY Spectrum (600 MHz, CD_3CN, 298 K) of **[2]R**•$9PF_6$

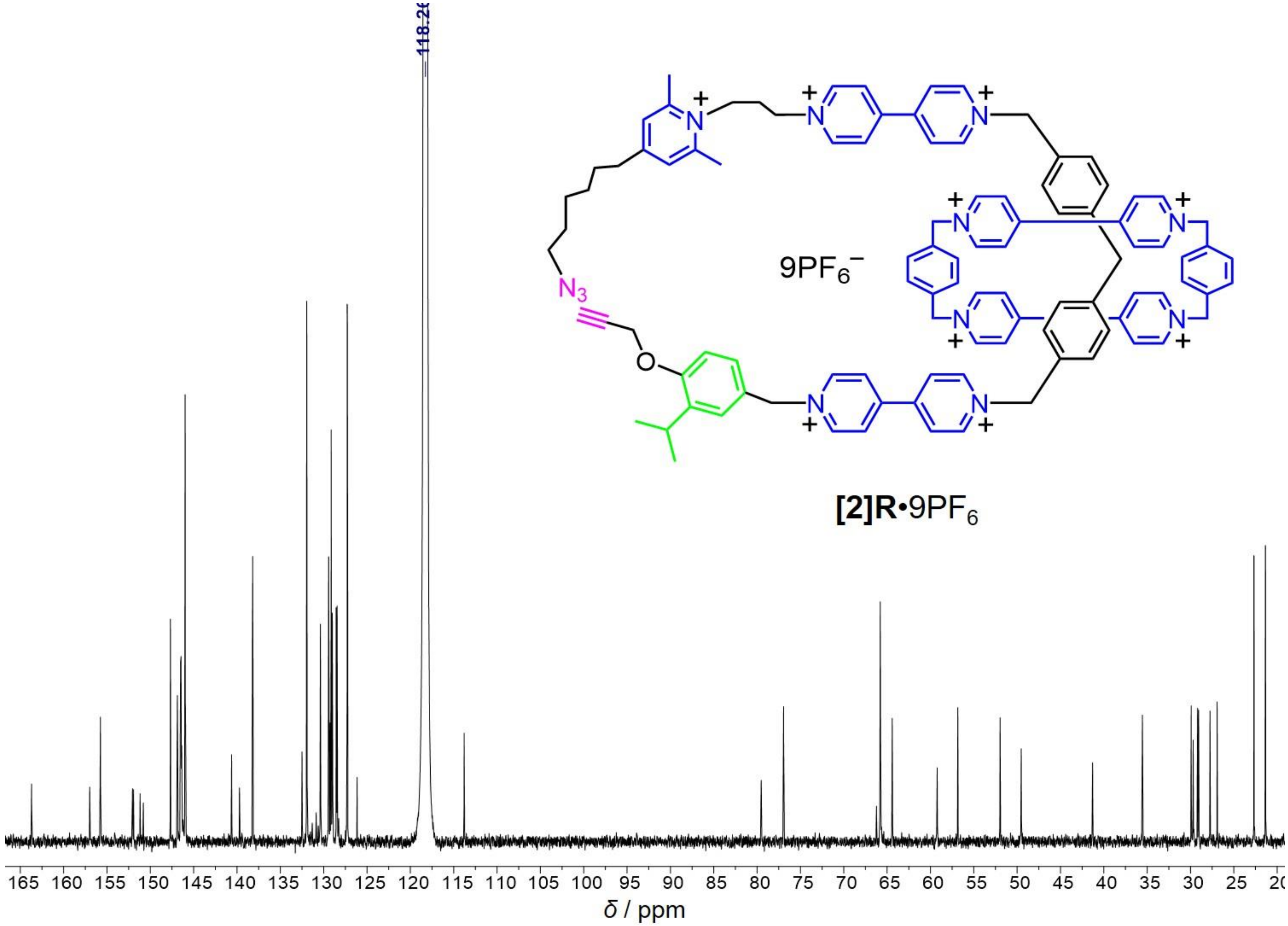

Supplementary Fig. 15 | ^{13}C NMR Spectrum (125 MHz, CD_3CN, 298 K) of **[2]R**•$9PF_6$

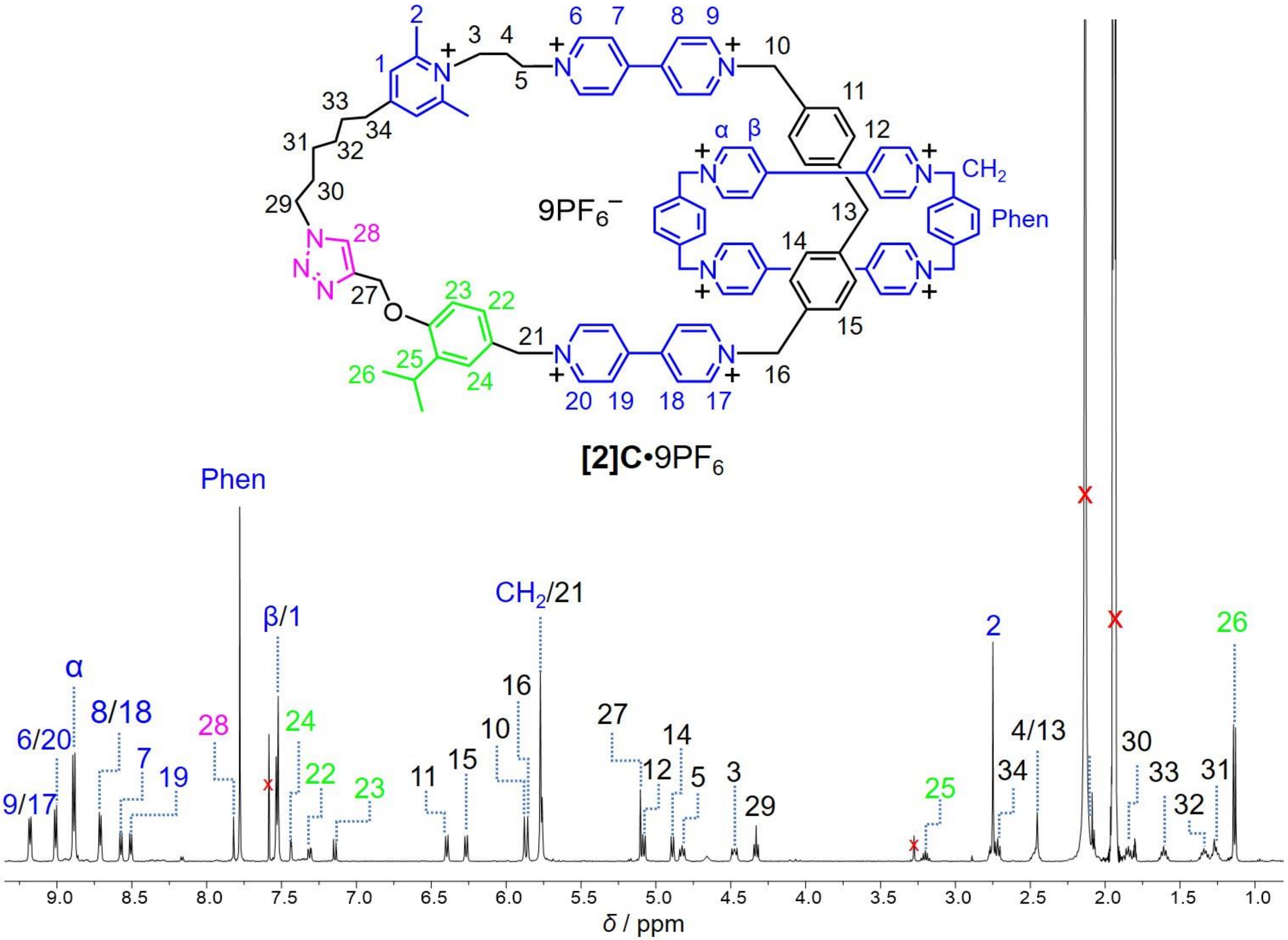

Supplementary Fig. 16 | ^{1}H NMR Spectrum (500 MHz, CD_3CN, 298 K) of **[2]C**•$9PF_6$

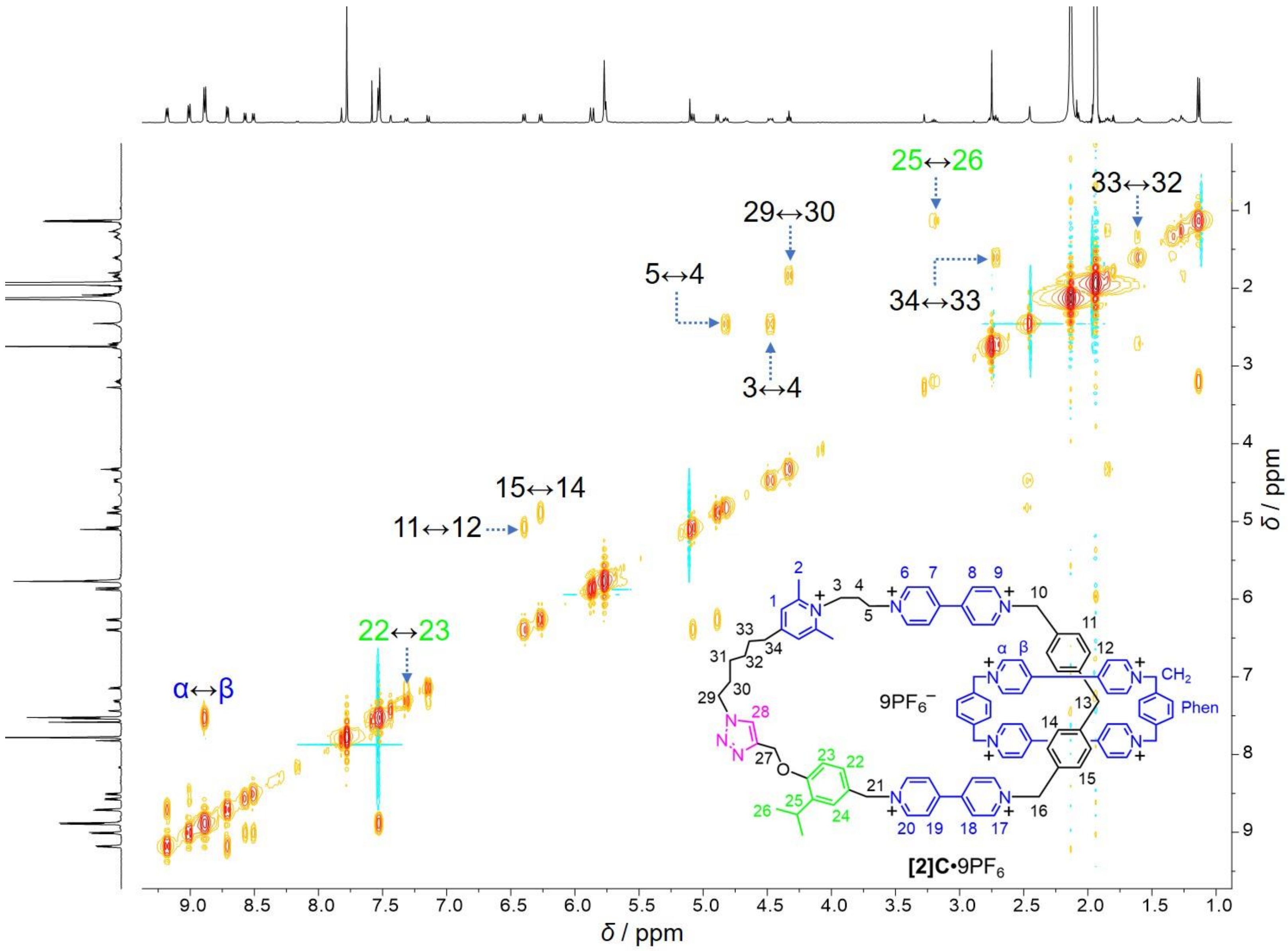

Supplementary Fig. 17 | ^{1}H-^{1}H COSY Spectrum (500 MHz, CD_3CN, 298 K) of **[2]C**•$9PF_6$

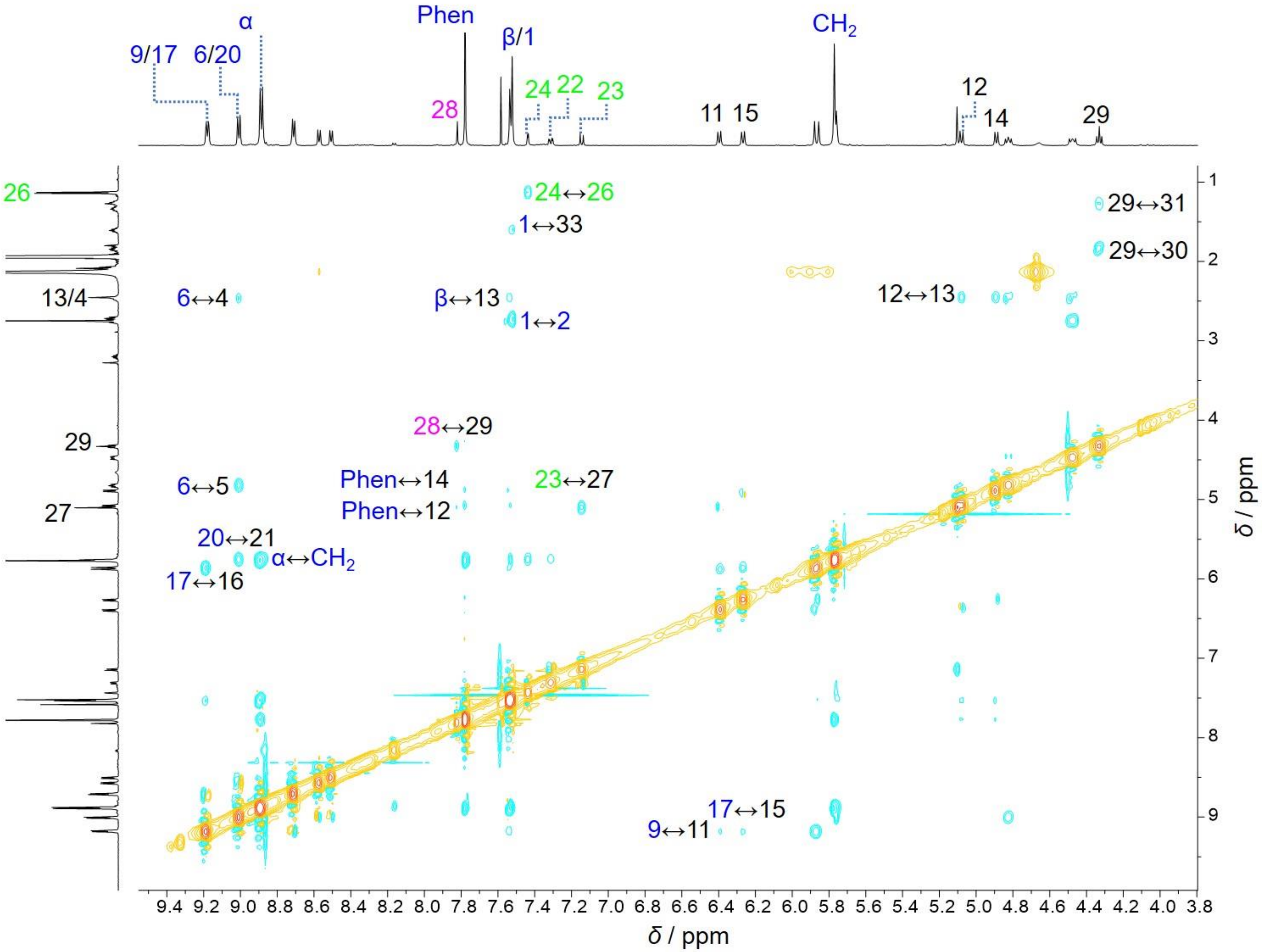

Supplementary Fig. 18 | ^{1}H-^{1}H NOESY Spectrum (500 MHz, CD_3CN, 298 K) of **[2]C**•9PF_6

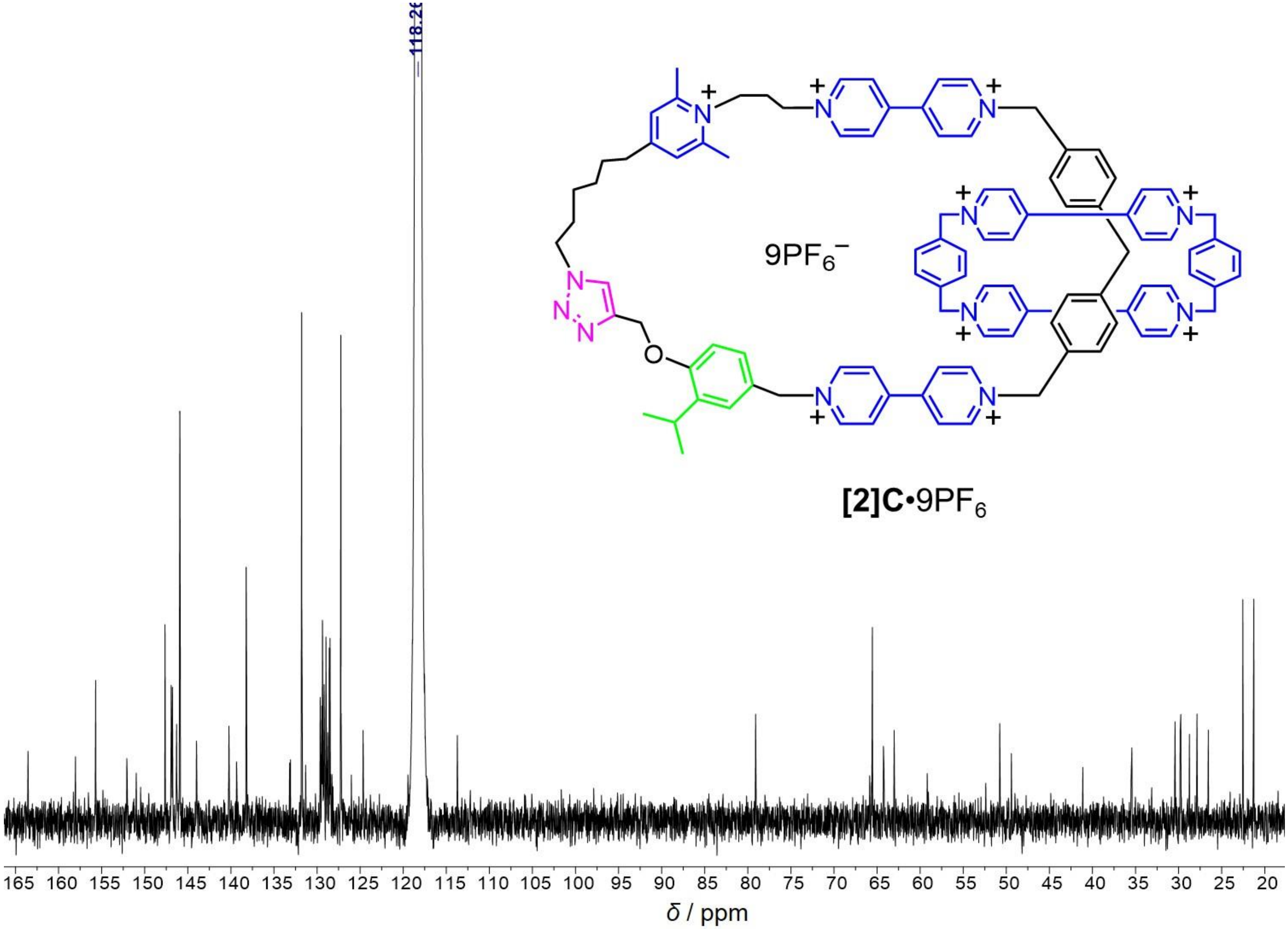

Supplementary Fig. 19 | ^{13}C NMR Spectrum (125 MHz, CD_3CN, 298 K) of **[2]C**•$9PF_6$

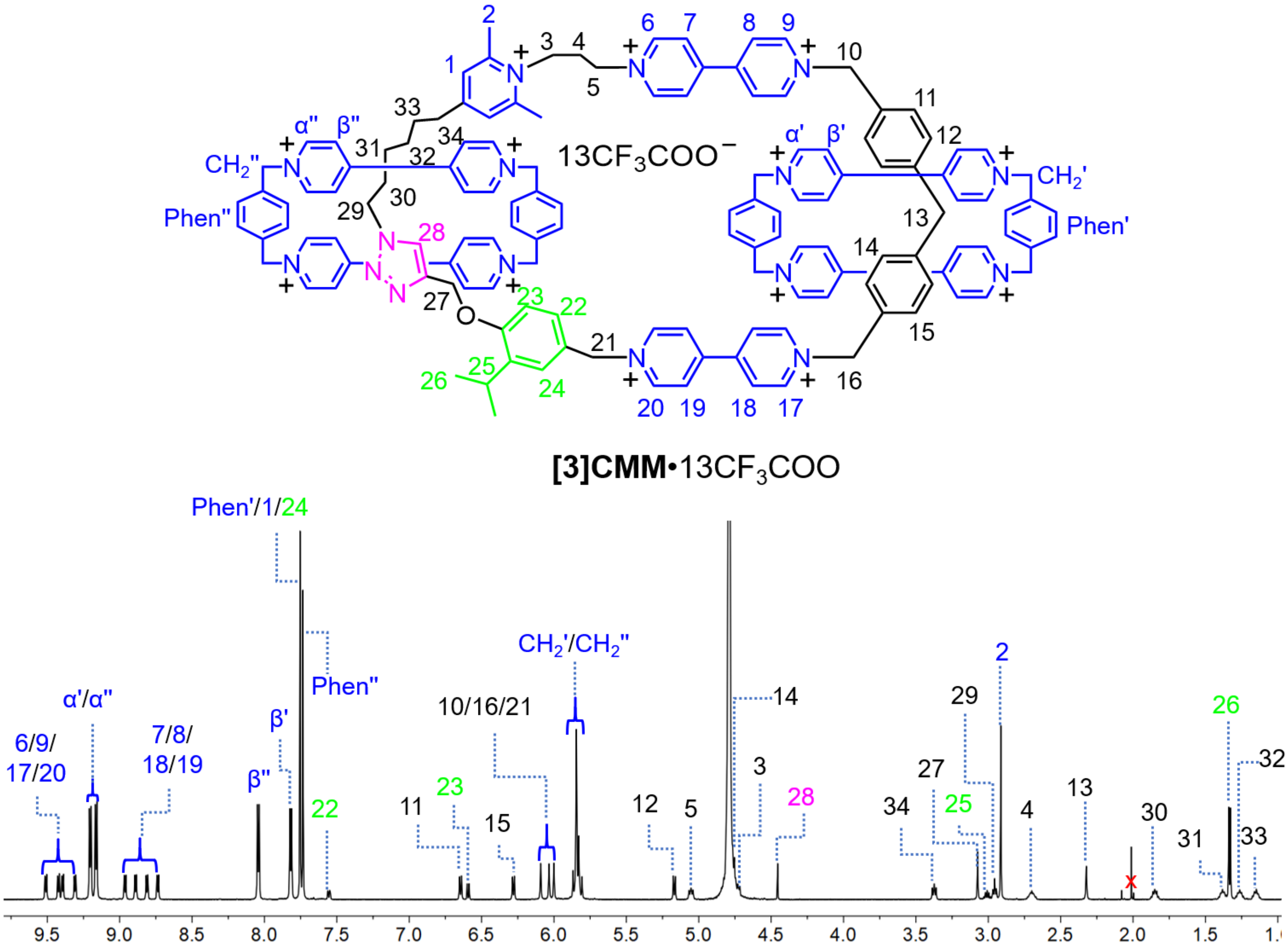

Supplementary Fig. 20 | ^{1}H NMR Spectrum (600 MHz, D_2O, 298 K) of **[3]CMM**•$13CF_3COO$

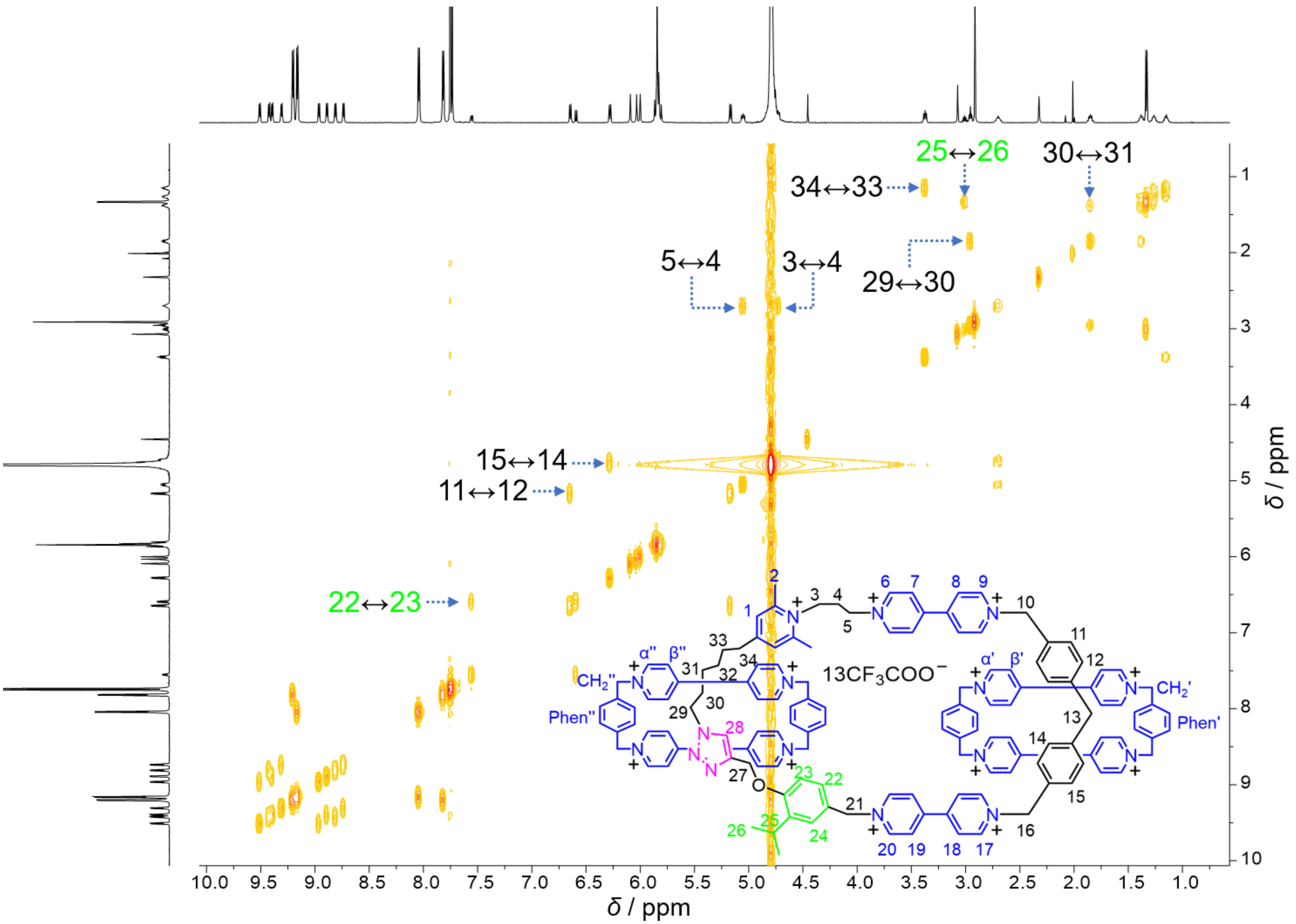

Supplementary Fig. 21 | ^{1}H-^{1}H COSY Spectrum (500 MHz, D_2O, 298 K) of **[3]CMM**•13CF_3COO

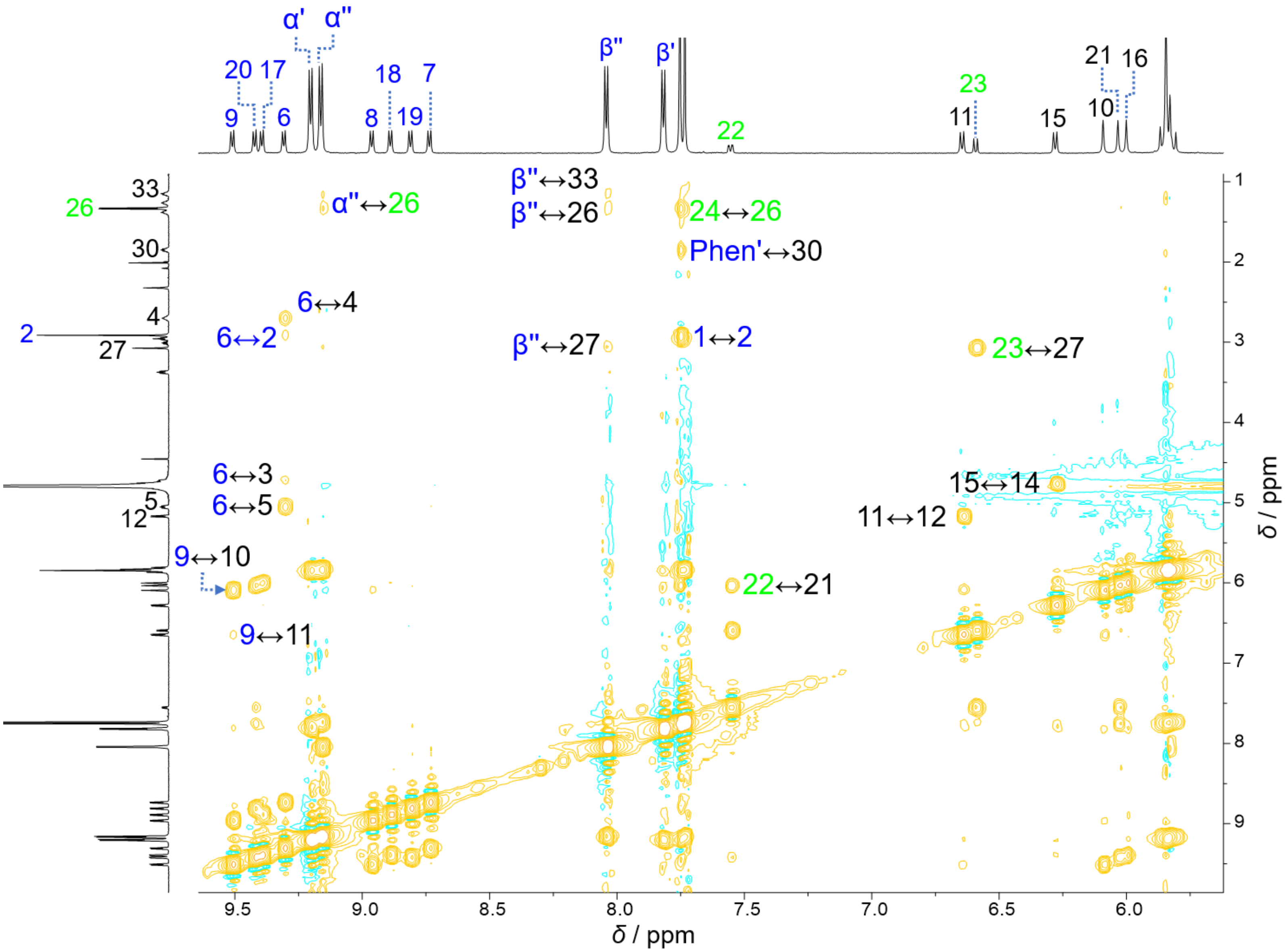

Supplementary Fig. 22 | Partial ^{1}H-^{1}H NOESY spectrum (500 MHz, D_2O, 298 K) of **[3]CMM**•$13CF_3COO$

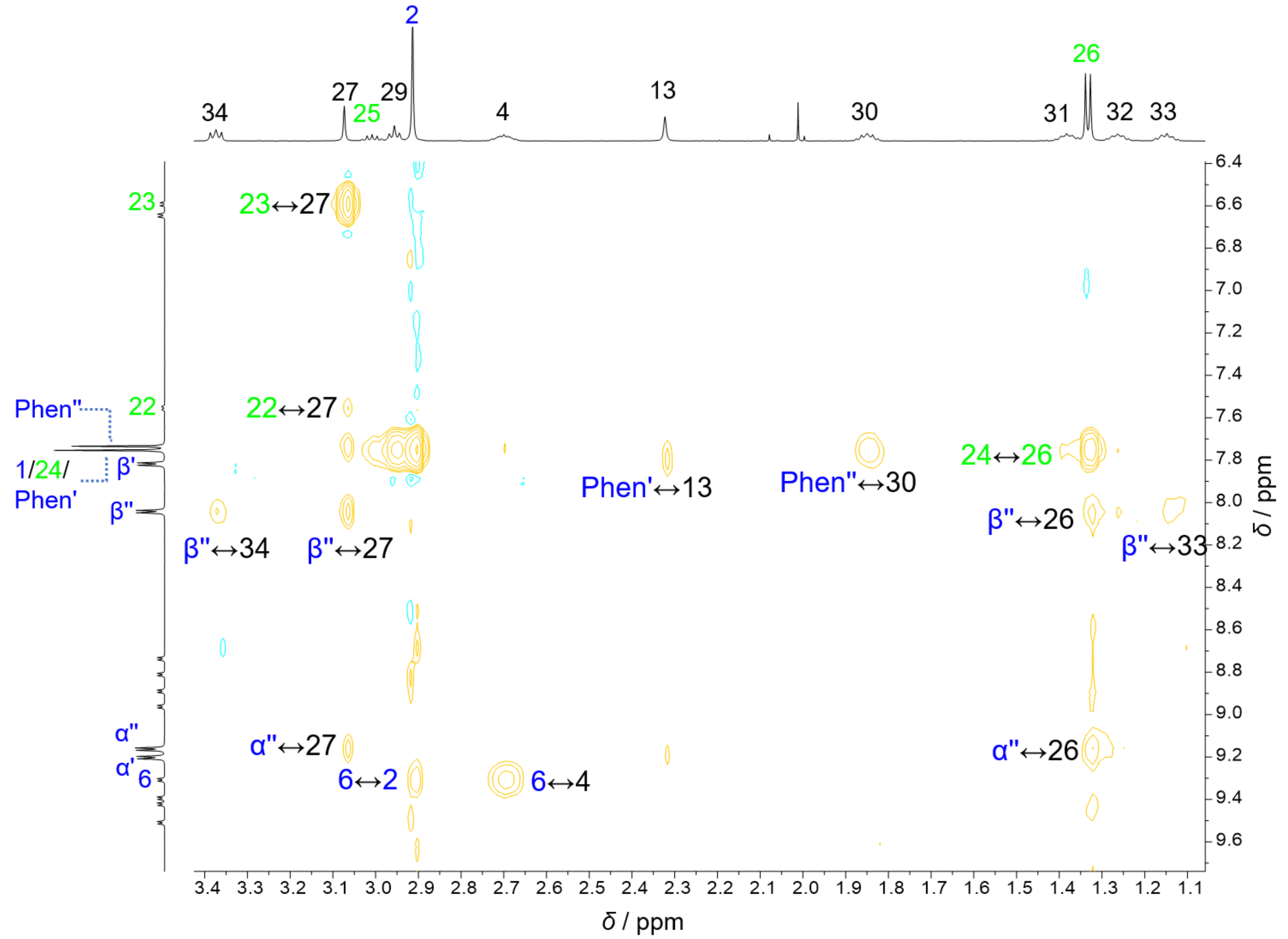

Supplementary Fig. 23 | Partial ^{1}H-^{1}H NOESY spectrum (500 MHz, D_2O, 298 K) of **[3]CMM•**13CF_3COO

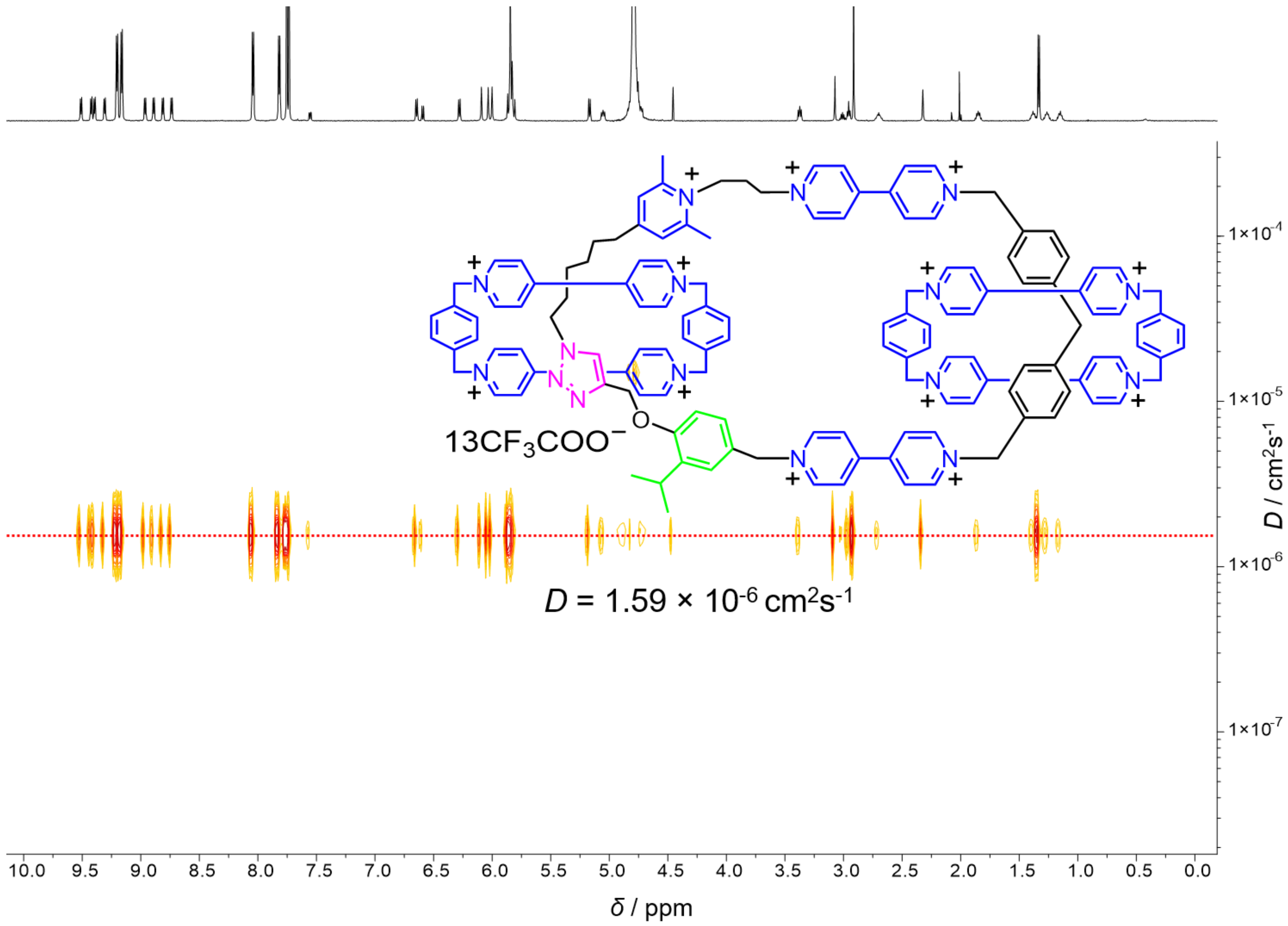

Supplementary Fig. 24 | ^{1}H DOSY Spectrum (600 MHz, D_2O, 298 K) of **[3]CMM**•13CF_3COO

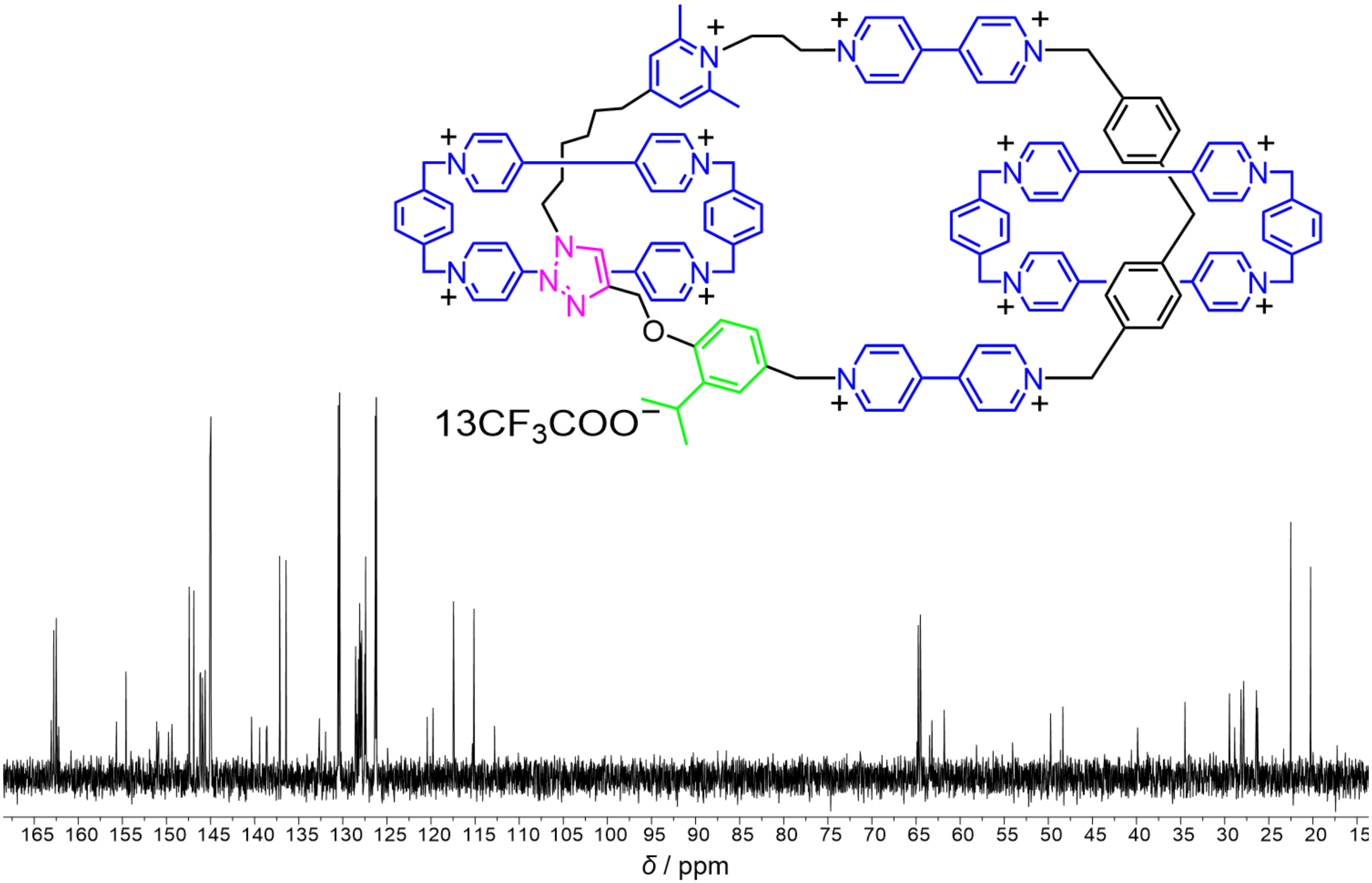

Supplementary Fig. 25 | ^{13}C NMR Spectrum (125 MHz, D_2O, 298 K) of **[3]CMM**•13CF_3COO

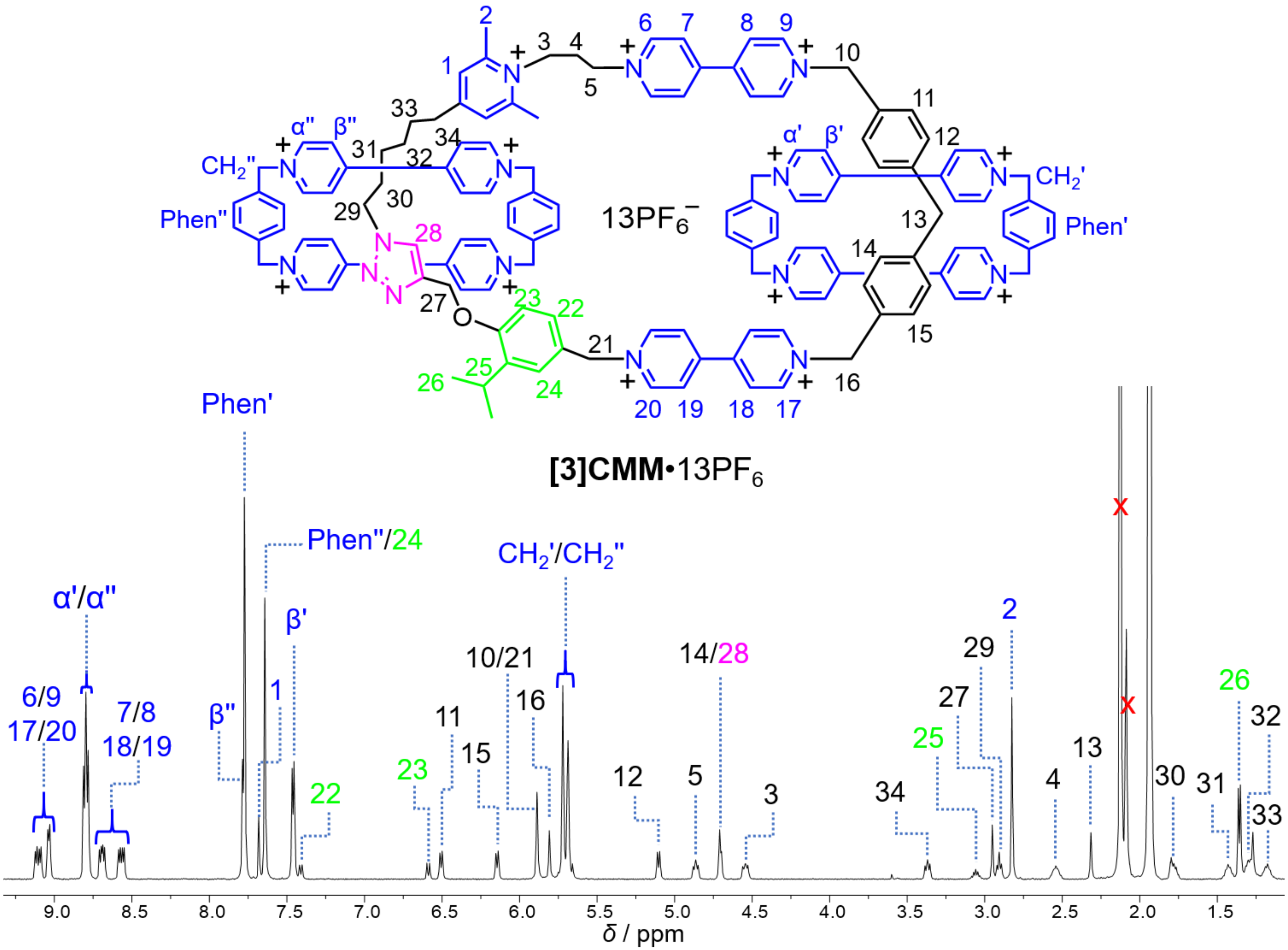

Supplementary Fig. 26 | ^{1}H NMR Spectrum (500 MHz, CD_3CN, 298 K) of **[3]CMM**•$13PF_6$

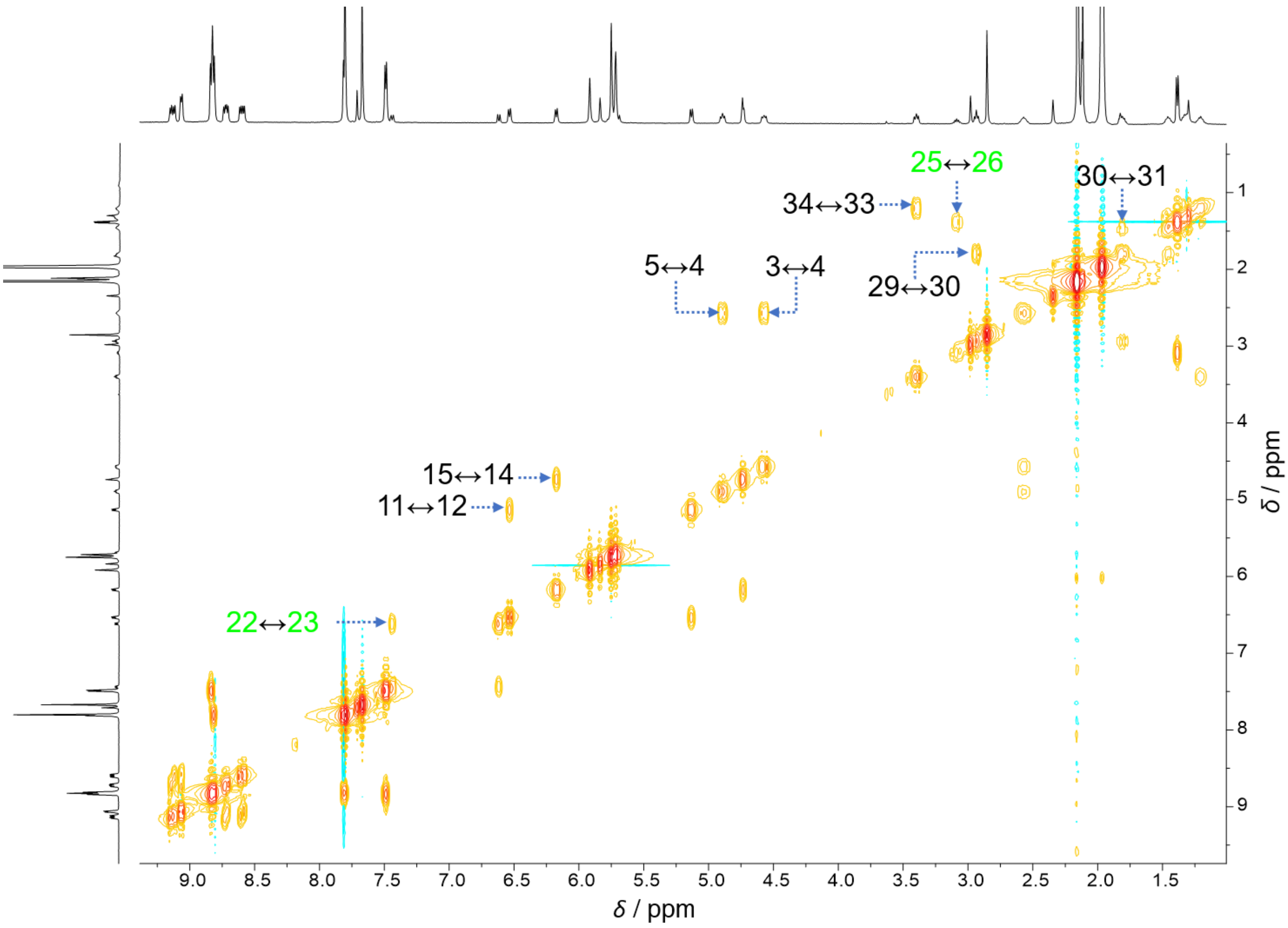

Supplementary Fig. 27 | ^{1}H-^{1}H COSY Spectrum (500 MHz, CD_3CN, 298 K) of **[3]CMM**•$13PF_6$

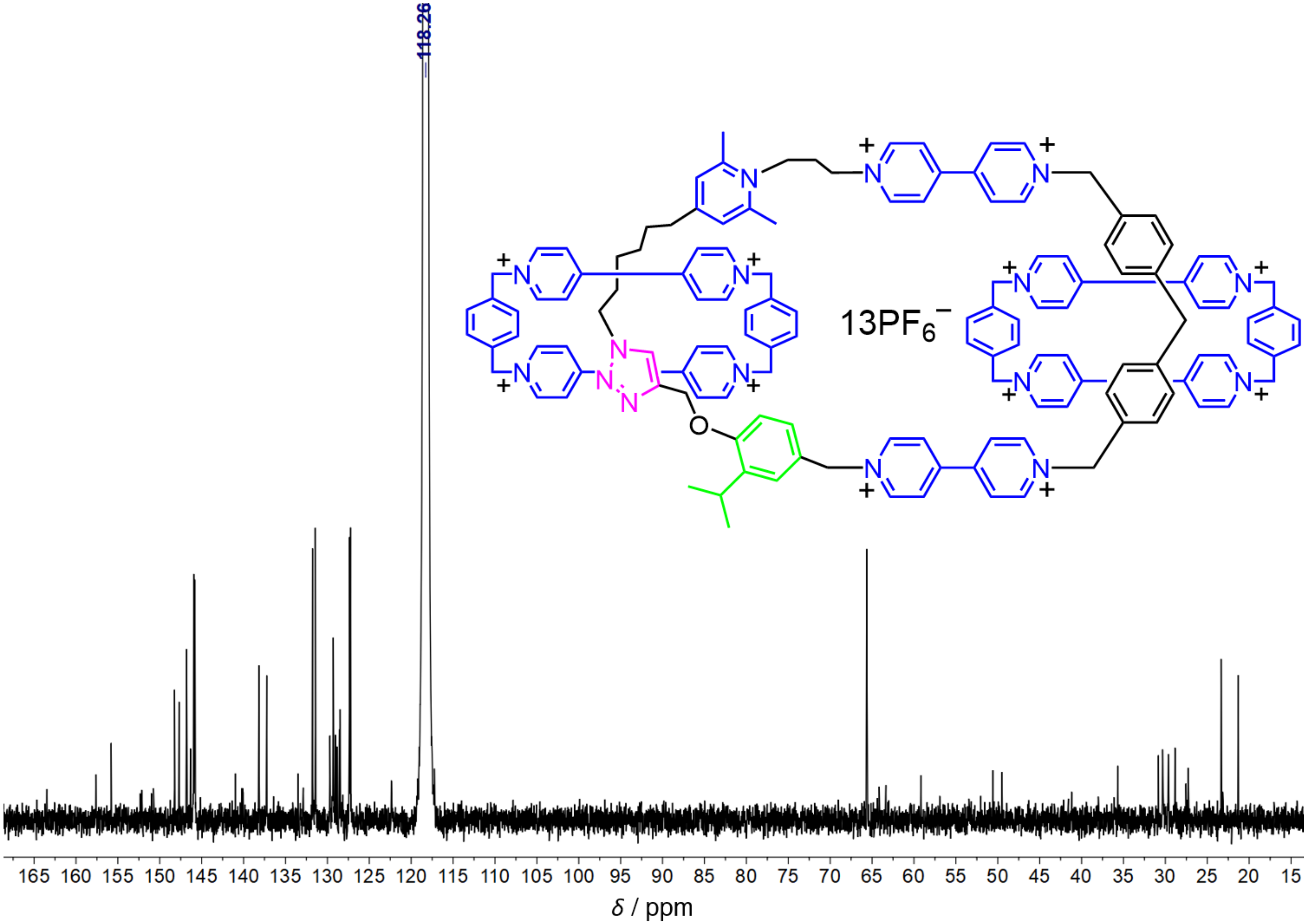

Supplementary Fig. 28 | ^{13}C NMR Spectrum (125 MHz, CD_3CN, 298 K) of **[3]CMM**•13PF_6

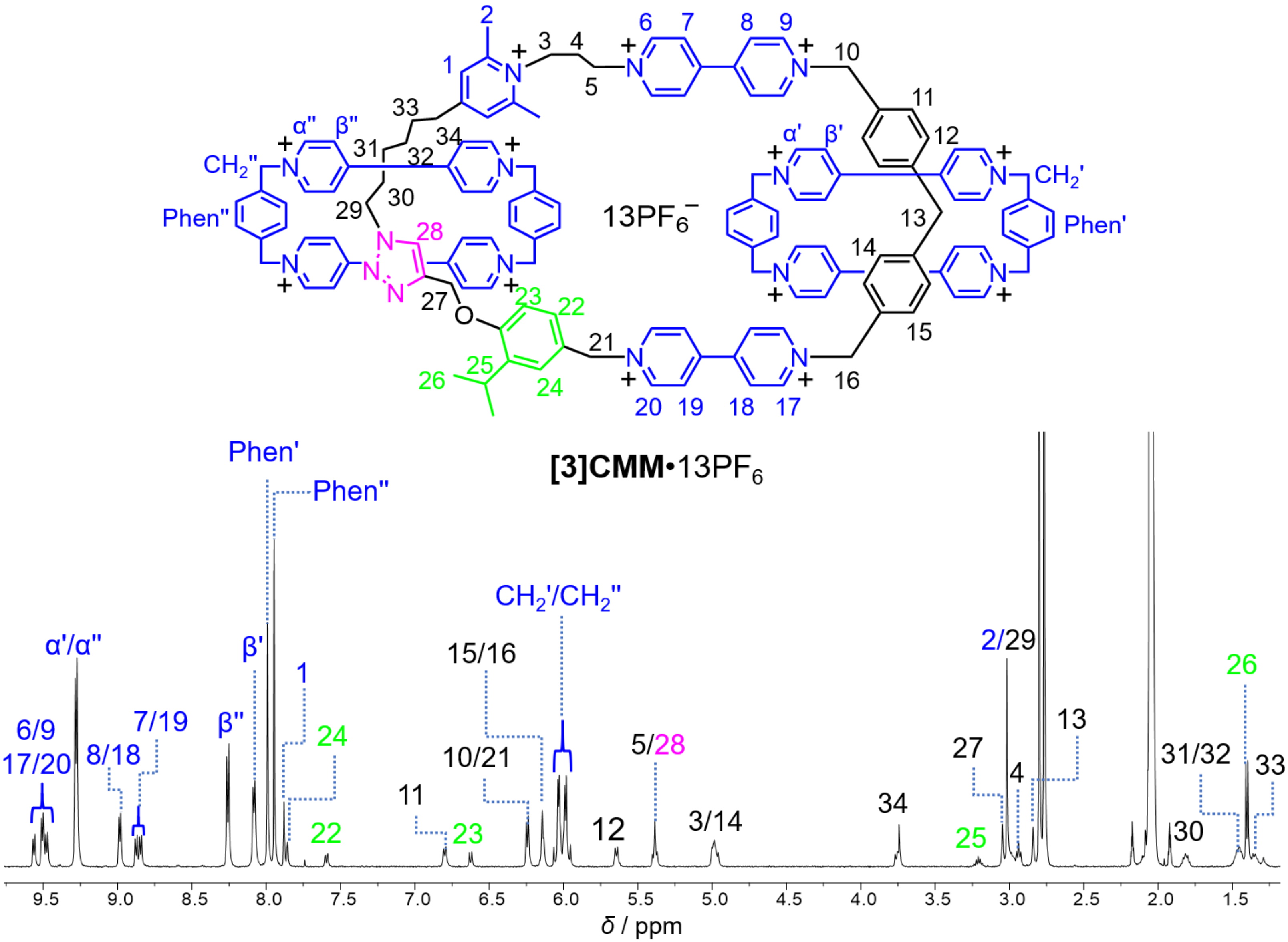

Supplementary Fig. 29 | ^{1}H NMR Spectrum (500 MHz, CD_3COCD_3, 298 K) of **[3]CMM**•$13PF_6$

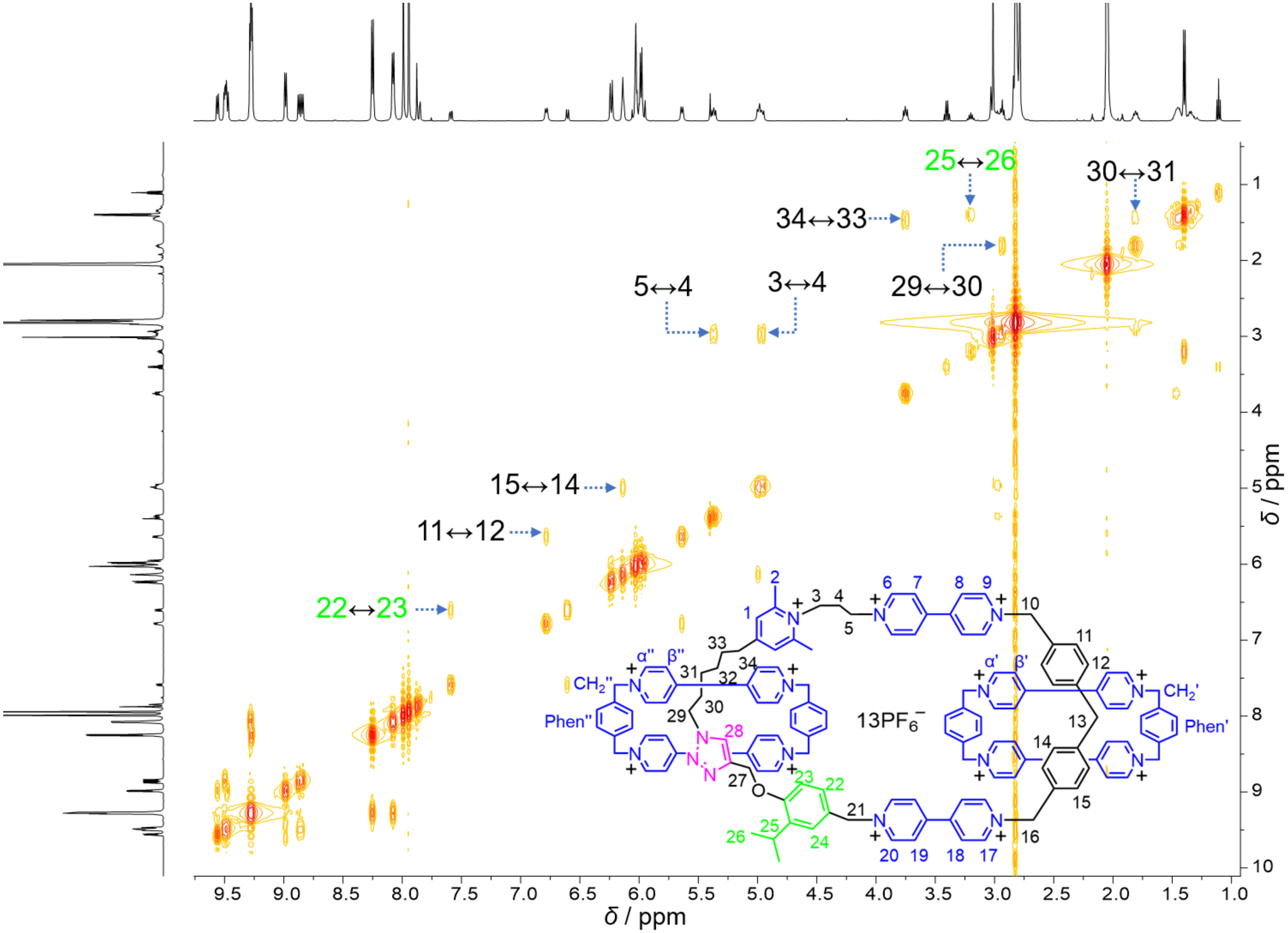

Supplementary Fig. 30 | ^{1}H-^{1}H COSY NMR Spectrum (500 MHz, CD_3COCD_3, 298 K) of **[3]CMM**•13PF$_6$

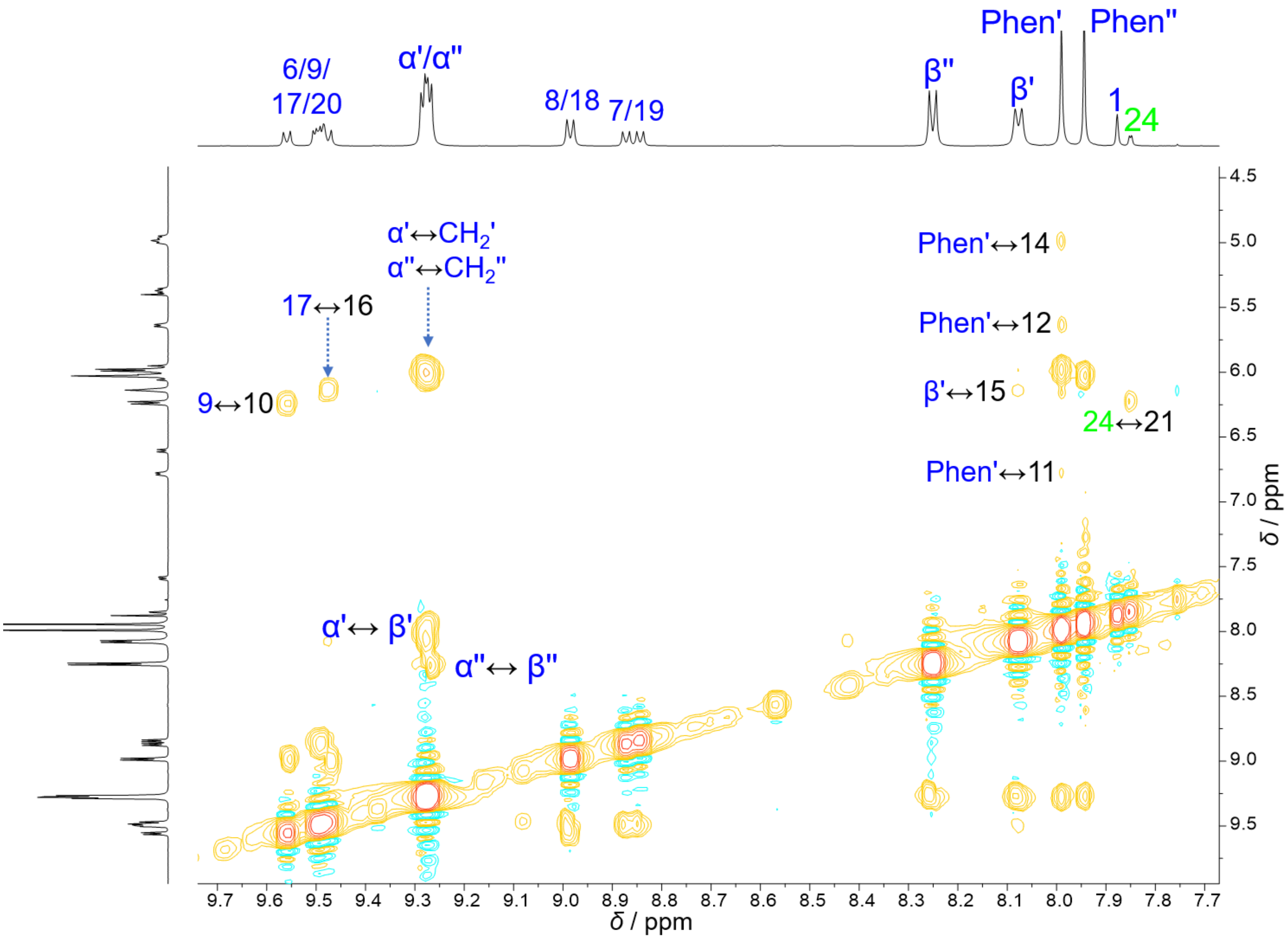

Supplementary Fig. 31 | Partial ^{1}H-^{1}H NOESY NMR Spectrum (500 MHz, CD_3COCD_3, 298 K) of **[3]CMM**•13PF_6

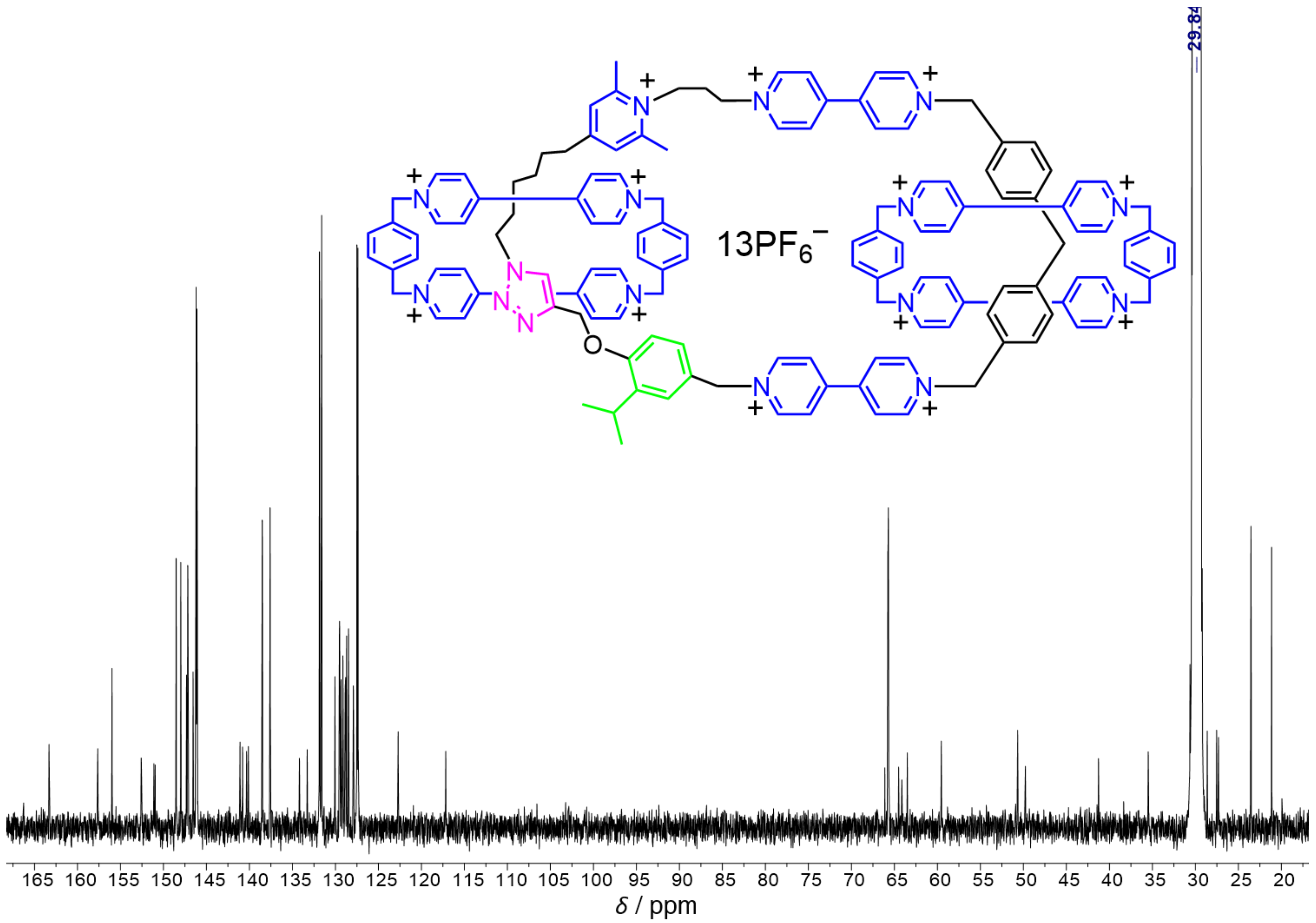

Supplementary Fig. 32 | ^{13}C NMR Spectrum (125 MHz, CD_3COCD_3, 298 K) of **[3]CMM**•13PF_6

4. Mass Spectrometry

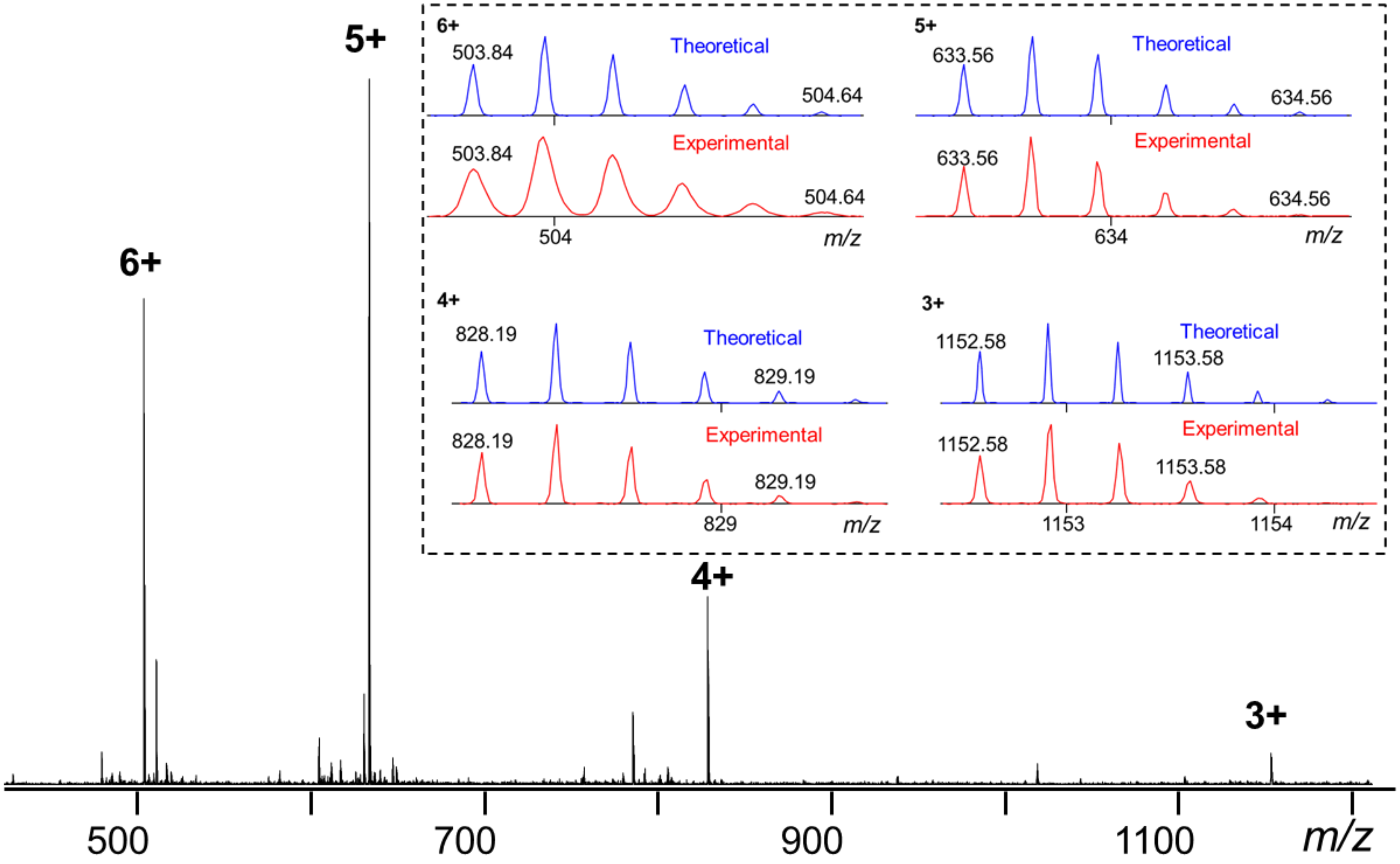

Supplementary Fig. 33 | Electrospray ionization-mass spectrum (ESI-MS) of **[3]CMM**•13PF_6. The insets in the dashed boxes show the theoretical (Top/Blue) and experimental (Bottom/Red) isotope pattern for $[M - 7PF_6]^{6+}$, $[M - 8PF_6]^{5+}$, $[M - 9PF_6]^{4+}$, and $[M - 10PF_6]^{3+}$, respectively, with their corresponding charge states. The insets in the dashed boxes show the experimental (Bottom / Red) isotope patterns for the four charged species, which match well with the theoretical simulations (Top / Blue). Experimental details for ESI-MS have been described in **Section 1. Materials and General Methods**.

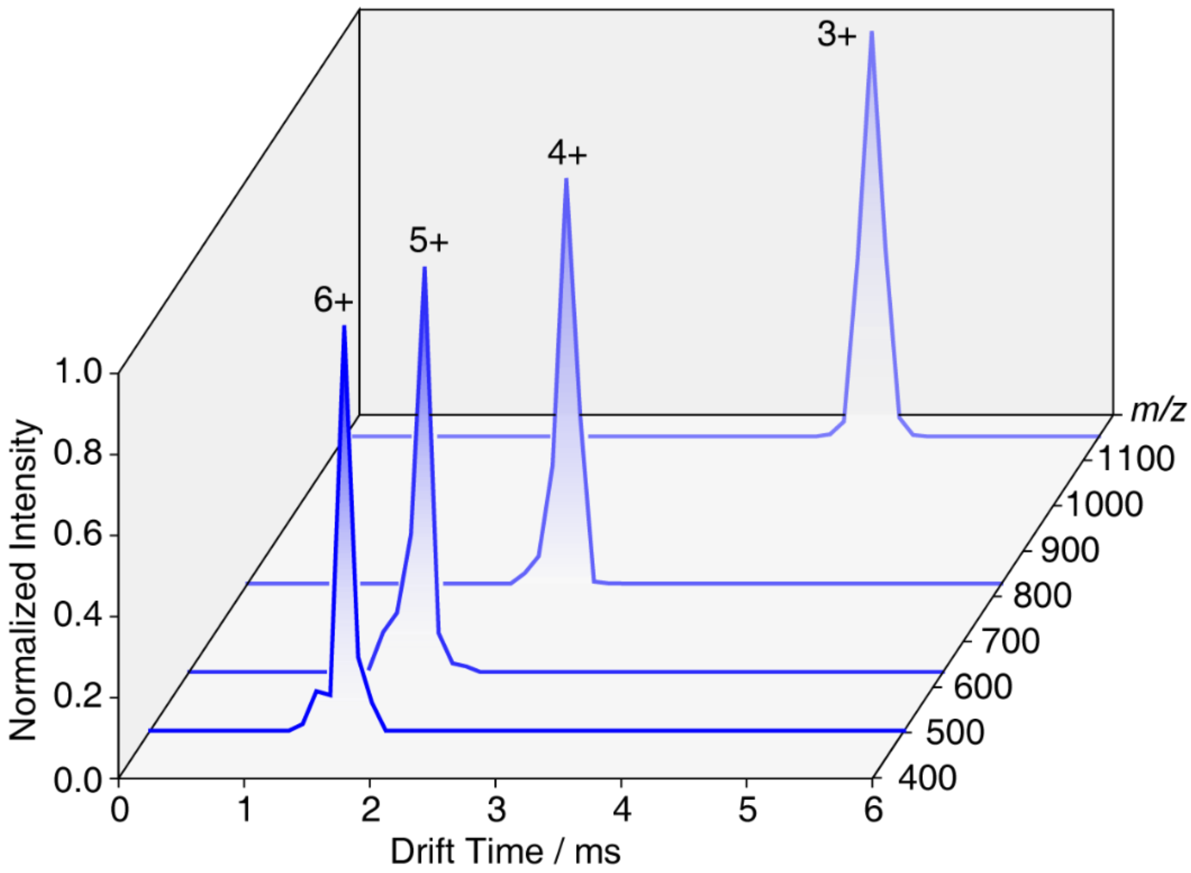

Supplementary Fig. 34 | 3D Plot of the travelling wave-ion mobility mass spectrometry (TWIM-MS, drift time versus *m*/*z* vs normalized intensity) of the [3]catenane **[3]CMM•**13PF$_6$. TWIM-MS could be regarded as a gas-phase separation which tells how charged ions drift under the influence of an electric field against a gas stream. The charged ions pass through the ion mobility cell—a N$_2$-filled RF ion guide and are separated by DC waves moving with certain velocity. Drift time is the amount of time ions take to pass the ion mobility cell, which is mainly dependent on the collision cross section (CCS) of the ions. Herein, each of the charged species gives only one signal with narrow drift time distribution, which excludes the existence of isomers and co-conformations in the oxidized state. The drift times were then fitted into a calibration curve, giving an average experimental CCS value of 604.9 Å^2, which matches the theoretical calculated CCS value of 599.5 Å^2. Detailed methods for the CCS calibration curve and theoretical CCS calculations have been described in detail in **Section 1. Materials and General Methods**.

5. The [2]Catenane Test

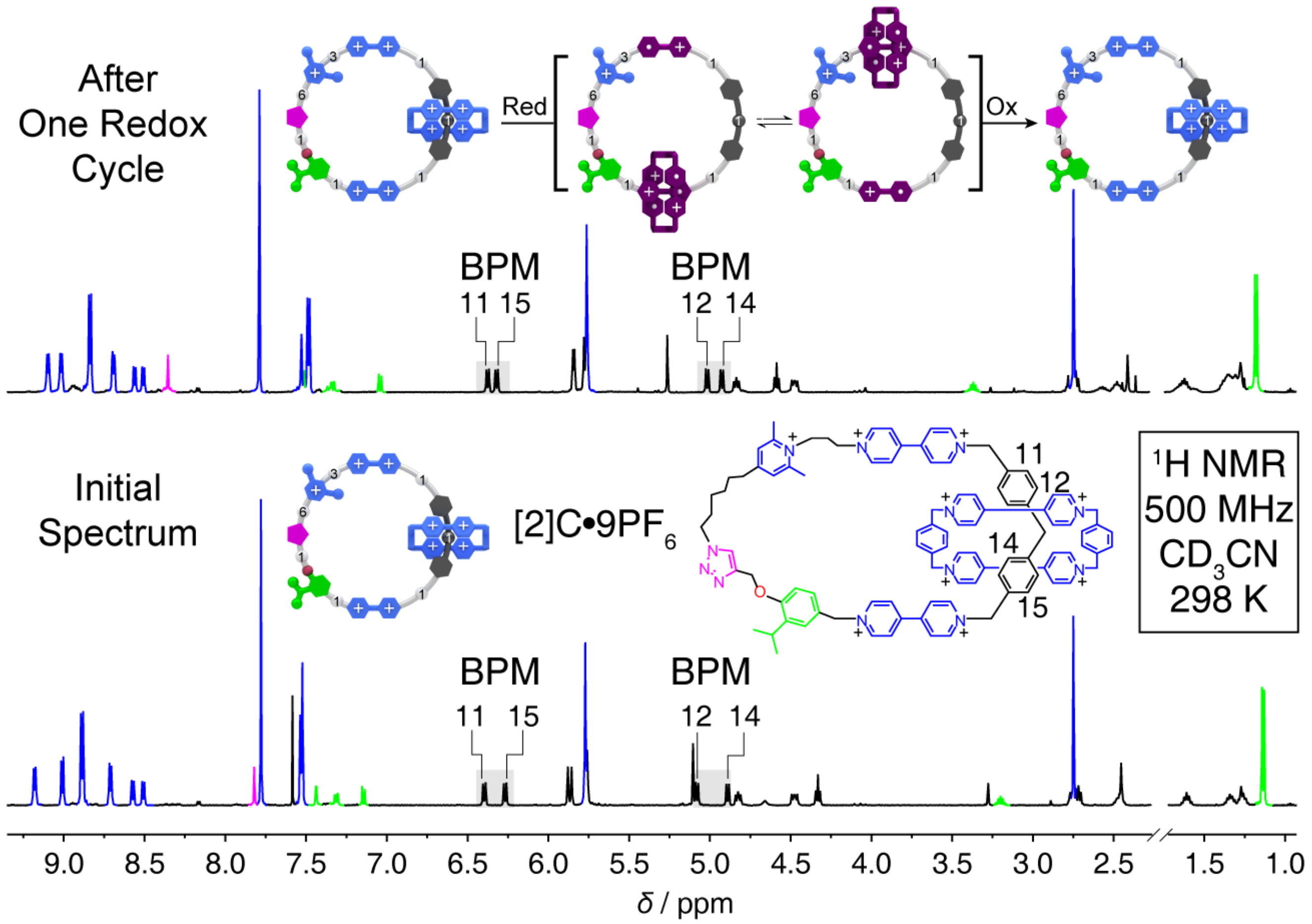

Supplementary Fig. 35 | ^{1}H NMR Spectra (500 MHz, CD_3CN, 298 K) of **[2]C**•9PF_6. Bottom: Initial spectrum. Top: After one redox cycle by using Zn dust (reduction) and an excess of $NOPF_6$ (oxidation). According to the ^{1}H NMR spectra, the CBPQT^{4+} ring does not change its position on the loop after one redox cycle, confirming that a single CBPQT^{4+} ring on the loop switches between one of the $V^{2+/+•}$ and the BPM unit during one redox cycle. Because the sample after one redox cycle has not been purified and contains Zn^{2+} cations in the solution, and the deuterated solvents (CD_3CN) for the redox reaction contains less water, as a result, there are some differences between the two ^{1}H NMR spectra.

6. Quantum Mechanical Calculations

In order to elucidate the working mechanism of the electric molecular motor, we performed Quantum Mechanical (QM) calculations at the level of Density Functional Theory (DFT) to study the potential energy surfaces (PESs) of the [2]catenanes **[2]C$^{9+/5+4•}$** and the [3]catenanes **[3]CMM$^{13+/7+6•}$**. In these calculations, we optimized the geometries in the Poisson-Boltzmann solvation model[8] at the level of M06-2X/6-31G*[9] with Jaguar 10.6[10]. Because of the complexity of the systems, we replaced all counterions by an implicit continuous dielectric solvent. In order to compensate the effect of counterions, parameters resulting in a stronger solvation effect were necessary. We obtained (Supplementary Fig. 36) the new solvation parameters by fitting the experimentally measured barrier height of the $CBPQT^{4+}$ dethreading from a model pseudorotaxane. We have listed the calculated barrier heights in Supplementary Fig. 36b. We chose the solvation parameters ε = 75 and R_0 = 1.4 Å for the calculations performed on **[2]C$^{9+/5+4•}$** and **[3]CMM$^{13+/7+6•}$**.

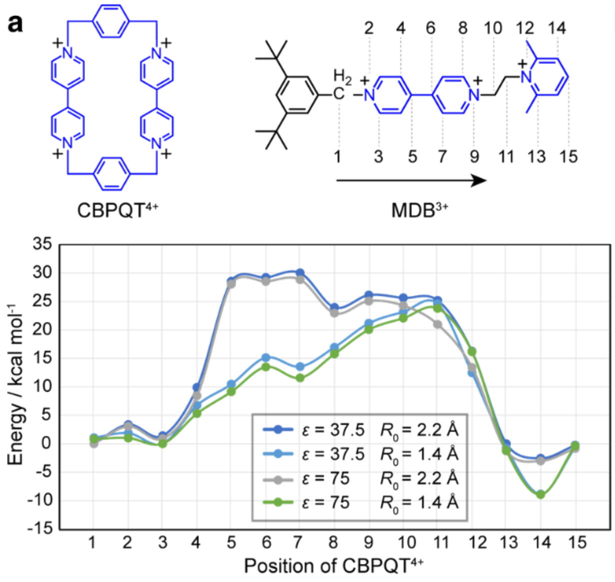

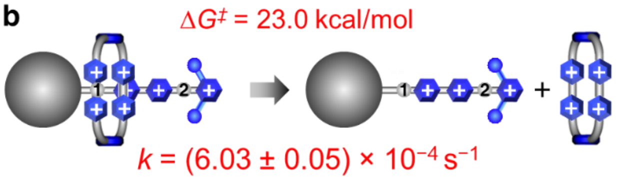

Dielectric Constant ε	Probe Radius R_0 / Å	Barrier Height / kcal mol^{-1}
37.5	2.2	30.0
37.5	1.4	24.6
75	2.2	28.8
**75**	**1.4**	**23.8**
Exp		23

Supplementary Fig. 36 | a, The potential energy surface of $CBPQT^{4+}$ moving on the model molecule MDB^{3+}. **b**, (Top) The experimentally measured kinetic results and (Bottom) the calculated barrier heights versus solvation parameters. The selected parameters are in boldface.

6.1 [2]Catenane

We studied the PESs of **[2]C$^{9+/5+4\bullet}$** by scanning the *z* coordinate from the center of CBPQT$^{4+/2(+\bullet)}$, which is defined as the average position of four methylene carbon atoms of CBPQT$^{4+/2(+\bullet)}$, passing over the atoms—labeled as position 0 to 50—on the loop. The PESs are periodic because of the cyclic nature of the loop.

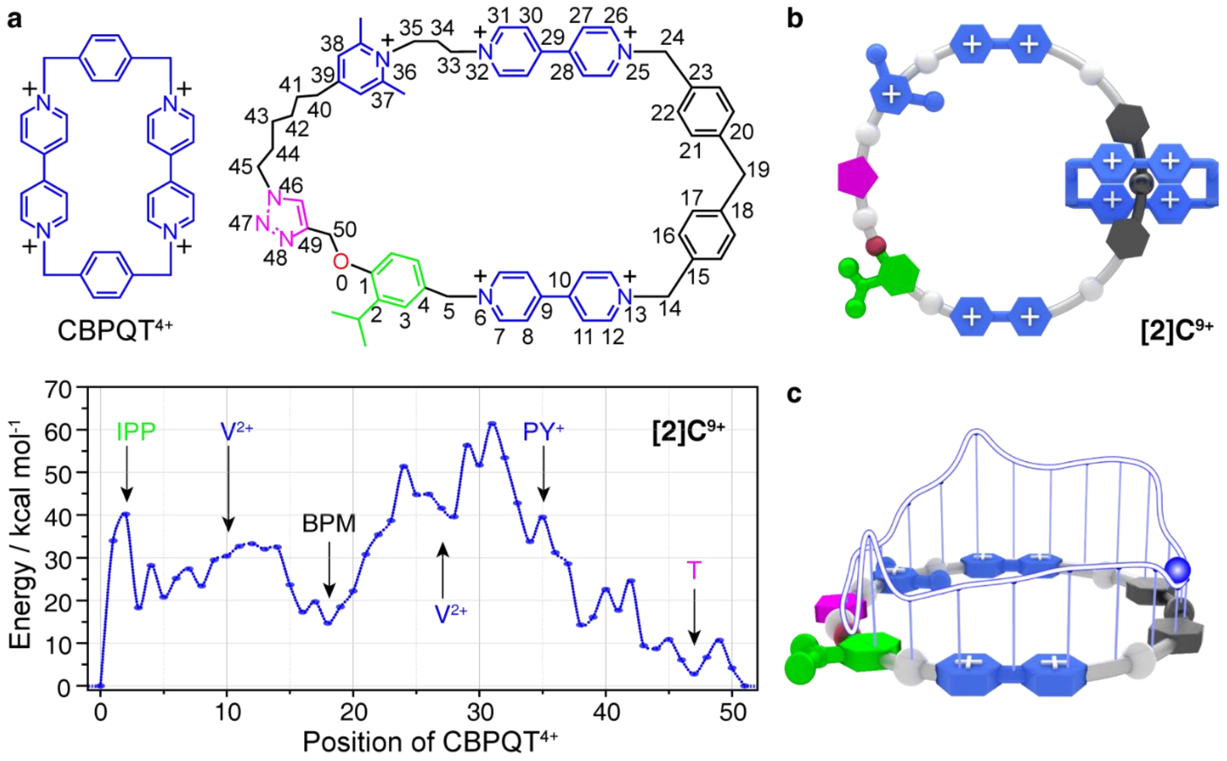

Supplementary Fig. 37 | a, The potential energy for the CBPQT^{4+} ring traversing the loop in the oxidized state. The numbered atoms on the loop are used to define the position of the CBPQT^{4+} ring. **b**, Graphical representation of the oxidized state of the [2]catenane **[2]C^{9+}**. **c**, Graphical representation of the calculated potential energy surface of the CBPQT^{4+} ring moving around the loop, displayed in a rollercoaster manner for the fully oxidized **[2]C**$^{9+}$.

For the oxidized state **[2]C^{9+}**, the PES (Supplementary Fig. 37 and Supplementary Table 1) reaches a maximum (position 31) between the positively charged V^{2+} and PY^{+} units, indicating strong electrostatic repulsion between the CBPQT^{4+} ring and the V^{2+}/PY^{+} units on the loop. The other barrier (position 2) is provided by the IPP unit (Supplementary Fig. 37) because of the bulky

isopropyl group. While the PES shows a minimum (position 0) positioned around the T unit, the other energy well (position 18) (Supplementary Fig. 37) is close to the center of the BPM unit because of donor-acceptor and van der Waals interactions between the $CBPQT^{4+}$ ring and the BPM unit. The energy barriers for the $CBPQT^{4+}$ ring encircling the BPM unit passing over the bulky IPP and the positively charged PY^{+} units are 25.5 and 46.7 kcal mol^{-1}, respectively.

Supplementary Table 1 | The calculated potential energies (kcal mol^{-1}) of the $CBPQT^{4+}$ ring as a function of its position on the loop in the oxidized state of the [2]catenane **[2]C^{9+}**

[2]C^{9+}			
Position of $CBPQT^{4+}$ Ring	**Potential Energy (kcal mol^{-1})**	Position of $CBPQT^{4+}$ Ring	**Potential Energy (kcal mol^{-1})**
0	**0**	26	**44.9**
1	**34.0**	27	**41.6**
2	**40.2**	28	**39.6**
3	**18.3**	29	**56.3**
4	**28.2**	30	**51.7**
5	**20.8**	31	**61.4**
6	**25.2**	32	**53.4**
7	**27.4**	33	**42.8**
8	**23.4**	34	**33.8**
9	**29.5**	35	**39.5**
10	**30.4**	36	**31.2**
11	**32.7**	37	**28.6**
12	**33.3**	38	**14.3**
13	**32.0**	39	**16.1**
14	**32.5**	40	**22.6**
15	**23.7**	41	**17.7**
16	**17.3**	42	**24.6**
17	**19.7**	43	**9.4**
18	**14.7**	44	**8.7**
19	**18.5**	45	**10.9**
20	**22.2**	46	**6.1**
21	**30.8**	47	**2.8**
22	**35.4**	48	**6.7**
23	**38.7**	49	**10.7**
24	**51.4**	50	**4.2**
25	**44.8**	0	**0**

For the reduced state **[2]C$^{5+4\bullet}$**, the PES (Supplementary Fig. 38 and Supplementary Table 2) reaches a maximum (42.2 kcal mol^{-1}) when the CBPQT$^{2(+\bullet)}$ ring passes (position 2) over the bulky IPP unit. All three wells (Supplementary Fig. 38) result from favorable radical-pairing interactions between the CBPQT$^{2(+\bullet)}$ ring and the V$^{+\bullet}$ units: the first (position 10) and the third (position 27) wells correspond to the CBPQT$^{2(+\bullet)}$ ring encircling the two V$^{+\bullet}$ units (Supplementary Fig. 38d), respectively, while the second well (position 21) has a compacted conformation (Supplementary Fig. 38d) because of the radical-pairing interactions between the V$^{+\bullet}$ units and the CBPQT$^{2(+\bullet)}$ ring, which are tilted with respect to each other.

Supplementary Table 2 | The calculated potential energies (kcal mol^{-1}) of the CBPQT$^{2(+\bullet)}$ ring as a function of its position on the loop in the reduced state of the [2]catenane **[2]C$^{5+4\bullet}$**

[2]C$^{5+4\bullet}$			
Position of CBPQT$^{2(+\bullet)}$ Ring	**Potential Energy (kcal mol^{-1})**	Position of CBPQT$^{2(+\bullet)}$ Ring	**Potential Energy (kcal mol^{-1})**
0	**19.4**	26	**14.9**
1	**16.1**	27	**5.8**
2	**42.2**	28	**6.2**
3	**17.7**	29	**13.9**
4	**18.6**	30	**14.6**
5	**18.1**	31	**22.5**
6	**12.8**	32	**22.2**
7	**7.4**	33	**24.4**
8	**8.0**	34	**15.9**
9	**6.8**	35	**15.8**
10	**0**	36	**25.5**
11	**7.0**	37	**14.2**
12	**13.5**	38	**9.9**
13	**16.2**	39	**10.5**
14	**16.1**	40	**8.7**
15	**12.8**	41	**11.5**
16	**7.9**	42	**11.4**
17	**9.4**	43	**10.7**
18	**10.0**	44	**11.5**
19	**12.5**	45	**19.6**
20	**5.4**	46	**18.6**
21	**1.5**	47	**10.4**
22	**8.6**	48	**11.0**
23	**11.7**	49	**11.4**
24	**14.2**	50	**11.9**
25	**14.7**	0	**19.4**

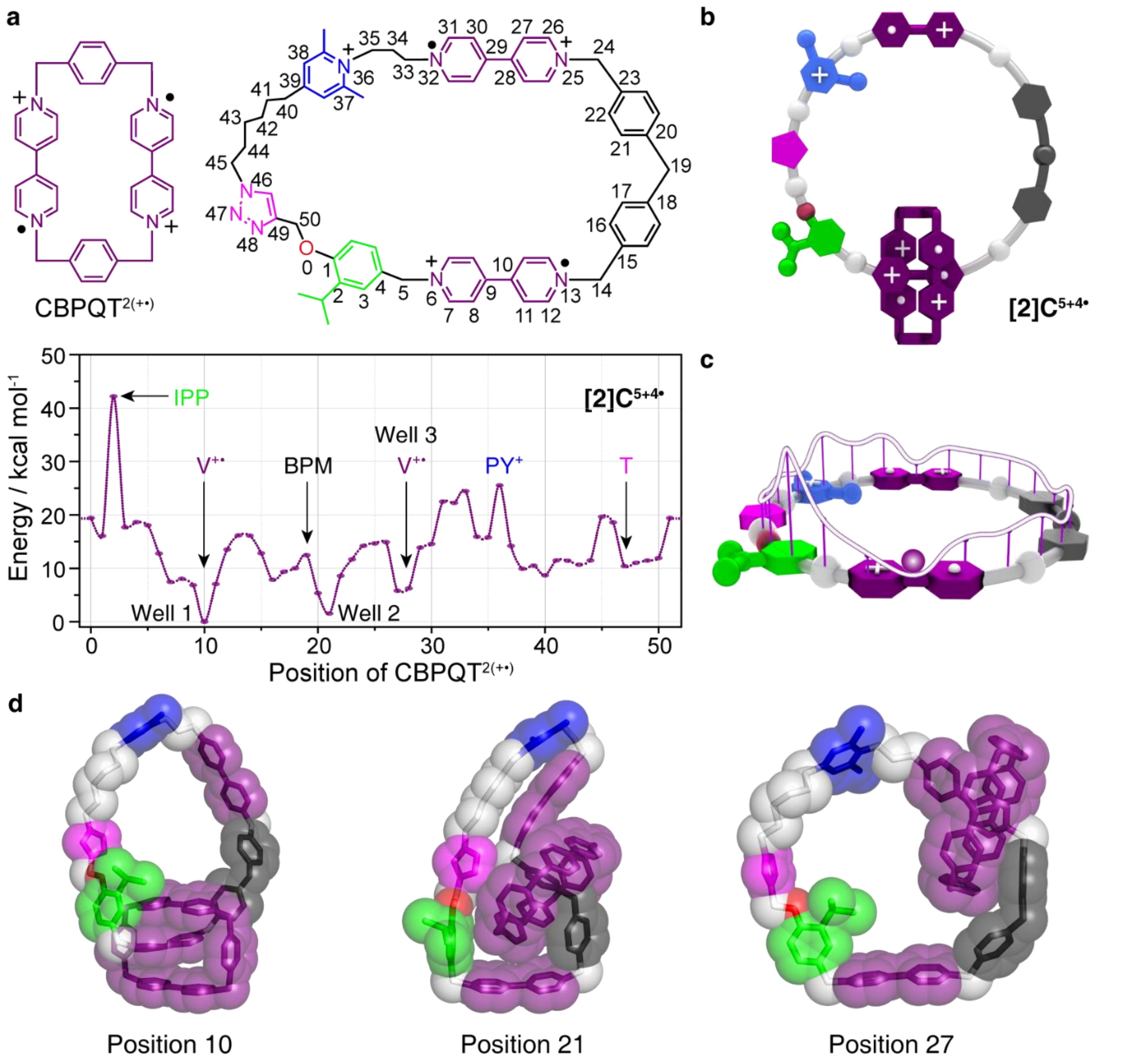

Supplementary Fig. 38 | a, The potential energy for the $CBPQT^{2(+•)}$ ring as a function of its position on the loop in the reduced state of the [2]catenane **$[2]C^{5+4•}$**. The numbered atoms on the loop are used to define the position of the $CBPQT^{2(+•)}$ ring. **b**, Graphical representation of the reduced state of [2]catenane **$[2]C^{5+4•}$** in its lowest energy co-conformation in which the $CBPQT^{2(+•)}$ ring encircles the $V^{+•}$ unit, position 10. **c**, Graphical representation of calculated potential energy of the $CBPQT^{2(+•)}$ ring as it moves around the loop, displayed in a rollercoaster manner for the radical state **$[2]C^{5+4•}$**. **d**, QM minimized structures (M06-2X/6-31G* basis set) for the $CBPQT^{2(+•)}$ ring located at positions 10, 21, and 27, respectively.

6.2 [3]Catenane

In order to describe the movement of the two $CBPQT^{4+/2(+•)}$ rings in **[3]CMM$^{13+/7+6•}$** around the loop, we constructed (Extended Data Fig. 5) a two-dimensional map, in which the x and y axis represent the positions of the $CBPQT^{4+/2(+•)}$ rings on the loop. Note that the map is periodic in both dimensions. In order to simplify the calculations, we moved one of the two $CBPQT^{4+/2(+•)}$ rings (ring A or ring B) to its next position, and then allowed the second ring to relax to its local minimum. The red diagonal dashed line is not only the symmetry line of the PES, but also represents the barrier that is physically uncrossable because two $CBPQT^{4+/2(+•)}$ rings would occupy the same space. The PESs of **[3]CMM$^{13+/7+6•}$** on the two-dimensional map were calculated by scanning the *z* coordinate from the center of one $CBPQT^{4+/2(+•)}$ ring passing over the labeled atoms on the loop while letting every other degree of freedom, including the position of the other $CBPQT^{4+/2(+•)}$ ring, relax to the local minimum. We calculated (Supplementary Tables 3–10) eight hypothetical paths for redox switching in the [3]catenanes **[3]CMM$^{13+/7+6•}$**, which are identified in Supplementary Figs. 39 and 40.

In the case of the reduction process (Supplementary Fig. 39), although all four paths experience a decrease in energy at the beginning, path R3 requires passage over the lowest barrier. In path R3, we moved the $CBPQT^{2(+•)}$ ring B first to the bottom $V^{+•}$ unit, followed by moving the $CBPQT^{2(+•)}$ ring A to pass (Supplementary Fig. 39e) over the PY^{+} unit to reach the end point II. In comparison, both path R2 and R4 require the $CBPQT^{2(+•)}$ ring A to pass (Supplementary Fig. 39f) over the bulky IPP unit from point I to II′. As a result, the path determining energy difference along path R4 is 4.7 kcal mol^{-1} higher than that along path R3. These results indicate that, under reducing conditions, the $CBPQT^{2(+•)}$ rings (1 and 2) strongly prefer to move from point I to II.

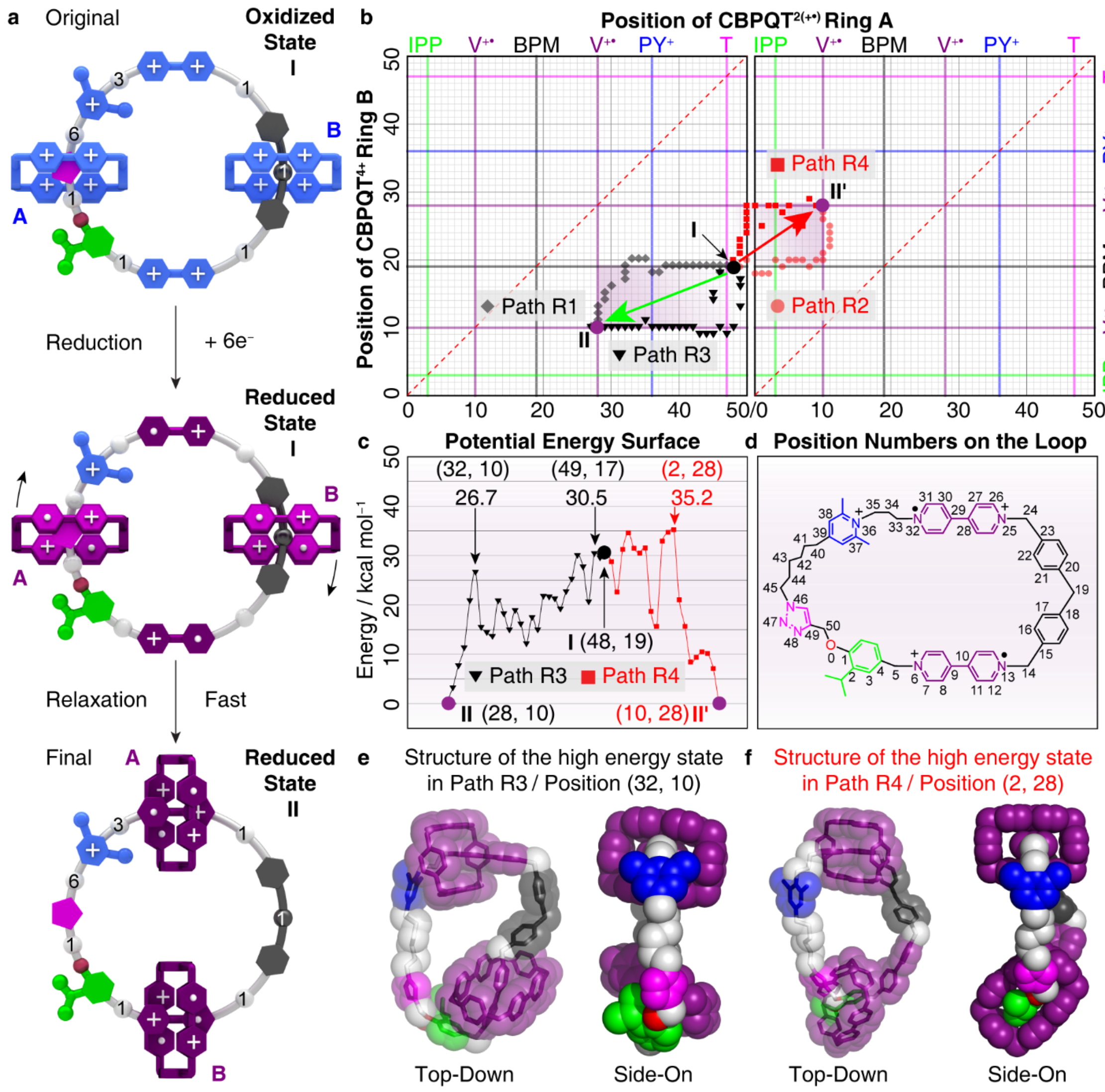

Supplementary Fig. 39 | a, Graphical representations of the redox process experienced by the [3]catenane in going from the oxidized state I (48, 19) to the reduced state II (28, 10). **b**, A 2D-position map describing the movement of the two reduced CBPQT$^{2(+\bullet)}$ rings (A and B) around the loop. Four hypothetical paths (R1–R4) during the reduction process are illustrated by diamond (gray), circle (light red), triangle (black), and square (red) symbols, respectively. The green arrow indicates the preferred direction of movement, and the red arrow indicates the less preferred (nearly precluded) direction of movement. The green, purple, black, blue, and magenta lines represent the position of IPP, V$^{+\bullet}$, BPM, PY^{+}, and T units, respectively. The dashed red diagonal lines represent barriers that cannot be crossed physically because doing so would require the two CBPQT$^{2(+\bullet)}$ rings to occupy the same space. **c**, The PESs of the two CBPQT$^{2(+\bullet)}$ rings moving around the loop in the reduced state, starting from point I (48, 19) and following paths R3 and R4, respectively. **d**, Structural formula of the loop with atoms numbered to define the positions of the CBPQT$^{2(+\bullet)}$ rings. **e** and **f**, QM minimized structures (M06-2X/6-31G* basis set, top-down and side-on views) for the CBPQT$^{2(+\bullet)}$ rings at positions (32, 10) and (2, 28), respectively.

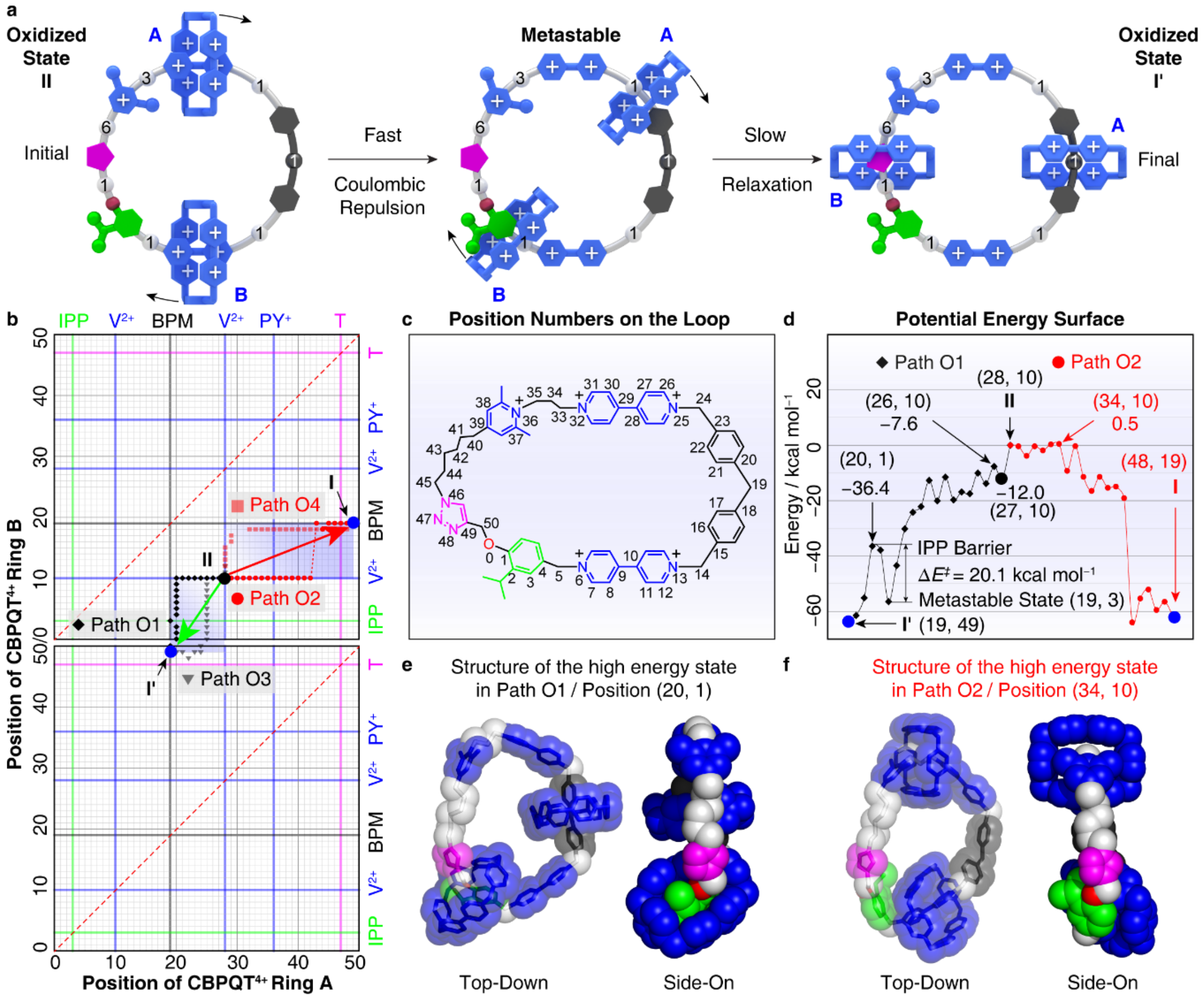

Supplementary Fig. 40 | a, Graphical representations of the process experienced by the [3]catenane in going from the oxidized state II (28, 10) to the oxidized state I′ (19, 49). **b**, A 2D-position map describing the movement of the two oxidized $CBPQT^{4+}$ rings (A and B) around the loop. Four hypothetical paths O1–O4 during the oxidation process are illustrated by diamond (black), circle (red), triangle (gray), and square (light red) symbols, respectively. The green arrow indicates the preferred direction of movement, and the red arrow indicates the less preferred (nearly precluded) direction of movement. The green, blue, black, blue, and magenta lines represent the positions of IPP, V^{2+}, BPM, PY^{+}, and T, respectively. The dashed red diagonal line represents a barrier that cannot be crossed physically because the two $CBPQT^{4+}$ rings would occupy the same space. **c**, Structural formula of the loop with atoms numbered to define the positions of the $CBPQT^{4+}$ rings. **d**, The PESs of the two $CBPQT^{4+}$ rings moving around the loop in the oxidized state starting from point II (28, 10) and following paths O1 and O2, respectively. The value for the energy barrier $\Delta E^{\ddagger}$ of 20.1 kcal mol^{-1} was determined from the energy difference between the position (19, 3) and (20, 1). The position (19, 3) corresponds to the metastable state on Path O1. **e** and **f**, QM minimized structures (M06-2X/6-31G* basis set, top-down and side-on views) for the $CBPQT^{4+}$ rings at positions (20, 1) and (34, 10), respectively. QM minimized structures of the lowest energy state (19, 48) and metastable state (21, 3) are presented in Fig. 2a and Fig. 4a, respectively.

The X-ray single crystal structure (Fig. 2b) of the reduced [3]catenane **[3]CMM$^{7+6•}$** shows clearly that two CBPQT$^{2(+•)}$ rings encircle the two V$^{+•}$ units in the loop. In order to study the unidirectional movement of the CBPQT^{4+} rings under oxidizing condition, we started from point II. The single crystal structure was also used as the initial structure for optimizing the geometry of the [3]catenane **[3]CMM^{13+}**. Upon oxidation, the two CBPQT^{4+} rings move away from the V^{2+} units. The question is—which direction is the more favorable one?

We examined (Supplementary Fig. 40) four paths that all start at point II in two different directions. Among the four paths, path O1 has the lowest energy barrier for the CBPQT^{4+} rings moving towards the end point I′ in which two CBPQT^{4+} rings encircle the T and BPM units, respectively. In path O1, we moved the CBPQT^{4+} ring B first of all to the BPM unit, followed by moving the CBPQT^{4+} ring A to the T unit so as to lower the overall energy. By comparison, in path O2, we moved the CBPQT^{4+} ring A first of all to pass over the PY^{+} unit to its final location at the T unit, followed by moving the CBPQT^{4+} ring A to the BPM unit. The path determining energy difference along path O2 is 8.1 kcal mol^{-1} higher than that along path O1. Therefore, it is far more favorable for the two CBPQT^{4+} rings to move from point II to point I′.

We conclude that, the Quantum Mechanical calculations predict unidirectional movement from point I → II → I′ during the redox cycle of the [3]catenane [3]CMM$^{13+/7+6•}$, which is consistent with our experimental result.

Regarding the Electrostatic Repulsion Energy between the Two CBPQT^{4+} Rings

Before we calculated the potential energies of the [3]catenane **[3]CMM^{13+}**, we hypothesized that the total energy (at each point) can be described as $E_{\text{total}} = E_{\text{A}} + E_{\text{B}} + E_{\text{AB}}$, where E_{A} and E_{B} denote the interaction between the loop and the rings (A and B) and E_{AB} is the electrostatic repulsion energy between the two rings in solution. Unexpectedly, the calculations reveal that this simple energy expression only captures the energy landscape with an accuracy of ~20 kcal/mol. Here we

chose to examine the potential energies of the [3]catenane **[3]CMM^{13+}** because the electrostatic repulsion between the two rings in the oxidized state **[3]CMM^{13+}** is much stronger than that in the case of the reduced state **[3]CMM$^{7+6•}$**.

We can obtain E_A and E_B for the [3]catenane **[3]CMM^{13+}** by removing one of the CBPQT^{4+} rings (B or A) completely and recalculating the potential energy. By plotting the difference between the E_{actual} and E_A+E_B (Supplementary Fig. 42), which is E_{AB} by definition, versus R_{AB} (defined by the distance between the centers of A and B), we found that the relation is complicated.

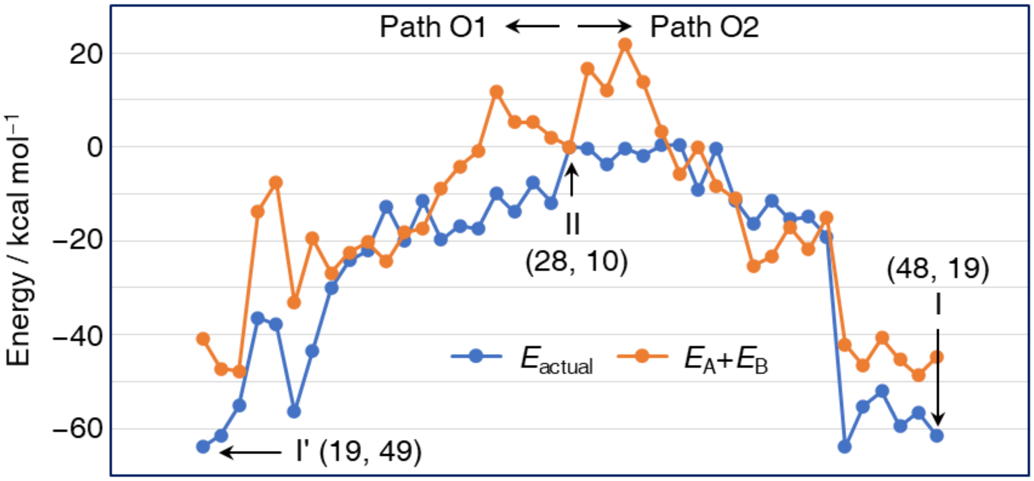

Supplementary Fig. 41 | The energies from the actual calculation (E_{actual}) versus the energies from the summation (E_A+E_B) following paths O1 and O2

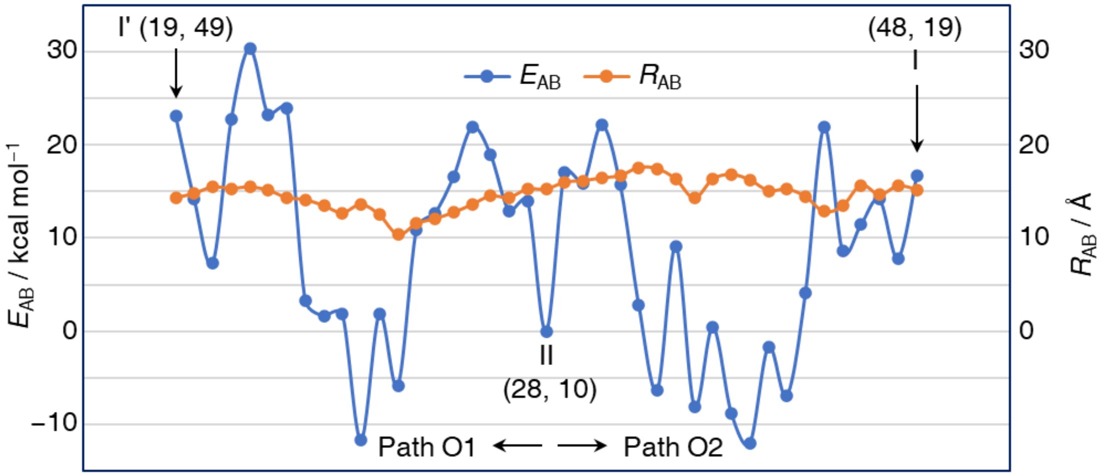

Supplementary Fig. 42 | The electrostatic repulsion energy E_{AB} between the two CBPQT^{4+} rings versus the distance R_{AB} between the centers of the two rings A and B following paths O1 and O2

By plotting (Supplementary Fig. 43) the energy difference of $\Delta E_{A/B}$ between the [3]catenane **[3]CMM^{13+}** and the [2]catenane **[2]C^{9+}**, we found that the E_A and E_B derived from the [3]catenane **[3]CMM^{13+}** deviates significantly from that for the [2]catenane **[2]C^{9+}** in certain regions — particularly the flexible alkyl chain and the hinge atoms between rigid fragments. This realization indicates that the presence of the other CBPQT^{4+} ring in these regions perturbs greatly the geometry of the [2]catenane by hindering the relaxation. This effect is at least as important as the electrostatic repulsion between two CBPQT^{4+} rings but is not included in the model of the [2]catenane.

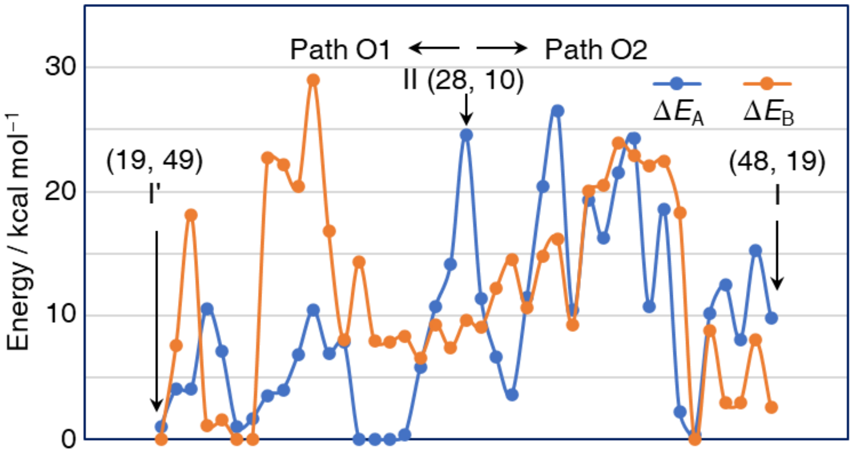

Supplementary Fig. 43 | The calculated potential energy difference between $E_{A/B}$ in the [3]catenane **[3]CMM^{13+}** and the [2]catenane **[2]C^{9+}** following paths O1 and O2

Supplementary Table 3 | The calculated potential energies (kcal mol^{-1}) of the [3]catenane **[3]CMM**$^{7+6\bullet}$ with the $CBPQT^{2(+\bullet)}$ rings A and B moving on the loop along the hypothetical path R1 from point I to II

**Path R1**		
Position of $CBPQT^{2(+\bullet)}$ Ring A	Position of $CBPQT^{2(+\bullet)}$ Ring B	**Potential Energy (kcal mol^{-1})**
48	19	**30.5**
47	19	**30.4**
46	18	**29.6**
46	19	**23.4**
45	19	**20.5**
44	19	**29.7**
43	19	**26.7**
42	19	**26.5**
41	19	**29.6**
40	19	**30.8**
39	19	**31.9**
38	19	**27.0**
37	18	**27.5**
36	18	**41.2**
35	20	**39.0**
34	20	**37.1**
33	20	**38.2**
32	19	**36.6**
32	18	**36.8**
32	17	**40.3**
30	16	**29.7**
29	15	**38.2**
29	14	**22.6**
28	13	**24.4**
28	12	**13.3**
28	11	**4.5**
28	10	**0**
27	10	**0.5**

Supplementary Table 4 | The calculated potential energies (kcal mol^{-1}) of the [3]catenane **[3]CMM$^{7+6\bullet}$** with the CBPQT$^{2(+\bullet)}$ rings A and B moving on the loop along the hypothetical path R2 from point I to II′

Path R2		
Position of CBPQT$^{2(+\bullet)}$ Ring A	Position of CBPQT$^{2(+\bullet)}$ Ring B	**Potential Energy (kcal mol^{-1})**
48	19	**30.4**
48	20	**28.8**
49	21	**22.6**
49	22	**31.2**
49	23	**34.6**
50	24	**31.5**
50	25	**30.5**
50	26	**31.5**
50	27	**18.7**
50	28	**15.6**
0	28	**32.9**
1	25	**34.7**
2	28	**35.2**
3	28	**21.0**
4	27	**15.6**
5	28	**8.4**
6	25	**9.4**
7	25	**10.5**
8	29	**10.2**
9	28	**7.1**
10	28	**0**

Supplementary Table 5 | The calculated potential energies (kcal mol^{-1}) of the [3]catenane **[3]CMM$^{7+6\bullet}$** with the $CBPQT^{2(+\bullet)}$ rings A and B moving on the loop along the hypothetical path R3 from point I to II

Path R3		
Position of $CBPQT^{2(+\bullet)}$ Ring A	Position of $CBPQT^{2(+\bullet)}$ Ring B	**Potential Energy (kcal mol^{-1})**
46	18	**29.6**
49	17	**30.5**
49	16	**20.7**
45	15	**27.8**
45	14	**30.1**
49	13	**25.3**
48	10	**22.8**
47	9	**19.3**
46	10	**21.3**
45	9	**21.7**
44	9	**21.8**
43	9	**15.0**
42	10	**17.5**
41	10	**12.2**
40	10	**16.2**
39	10	**19.0**
38	10	**15.0**
37	10	**18.3**
36	10	**20.9**
35	11	**13.7**
34	10	**14.5**
33	10	**15.5**
32	10	**26.7**
31	10	**20.9**
30	10	**11.4**
29	10	**7.7**
28	10	**3.3**
27	10	**0.5**

Supplementary Table 6 | The calculated potential energies (kcal mol^{-1}) of the [3]catenane **[3]CMM$^{7+6\bullet}$** with the CBPQT$^{2(+\bullet)}$ rings A and B moving on the loop along the hypothetical path R4 from point I to II′

Path R4		
Position of CBPQT$^{2(+\bullet)}$ Ring A	Position of CBPQT$^{2(+\bullet)}$ Ring B	**Potential Energy (kcal mol^{-1})**
48	19	**30.4**
48	20	**28.8**
49	21	**22.6**
49	22	**31.2**
49	23	**34.6**
50	24	**31.5**
50	25	**30.5**
50	26	**31.5**
50	27	**18.7**
50	28	**15.6**
0	28	**32.9**
1	25	**34.7**
2	28	**35.2**
3	28	**21.0**
4	27	**15.6**
5	28	**8.4**
6	25	**9.4**
7	25	**10.5**
8	29	**10.2**
9	28	**7.1**
10	28	**0**

Supplementary Table 7 | The calculated potential energies (kcal mol^{-1}) of the [3]catenane **[3]CMM^{13+}** with the CBPQT^{4+} rings A and B moving on the loop along the hypothetical path O1 from point II to I′

Path O1		
Position of CBPQT^{4+} Ring A	Position of CBPQT^{4+} Ring B	**Potential Energy (kcal mol^{-1})**
28	10	**0**
27	10	**−12.0**
26	10	**−7.6**
25	10	**−13.8**
24	10	**−10.0**
23	10	**−17.5**
22	10	**−16.9**
21	10	**−19.7**
20	10	**−11.5**
20	9	**−20.0**
20	8	**−12.7**
20	7	**−22.2**
20	6	**−24.2**
20	5	**−30.2**
20	4	**−43.4**
19	3	**−56.5**
20	2	**−37.8**
20	1	**−36.4**
20	0	**−55.1**
19	50	**−61.5**
19	49	**−63.9**

Supplementary Table 8 | The calculated potential energies (kcal mol^{-1}) of the [3]catenane **[3]CMM^{13+}** with the CBPQT^{4+} rings A and B moving on the loop along the hypothetical path O2 from point II to I

Path O2		
Position of CBPQT^{4+} Ring A	Position of CBPQT^{4+} Ring B	**Potential Energy (kcal mol^{-1})**
28	10	**0**
29	10	**−0.4**
30	10	**−3.8**
31	10	**−0.4**
32	10	**−1.9**
33	10	**0.3**
34	10	**0.5**
35	10	**−9.2**
36	10	**−0.3**
37	10	**−11.4**
38	10	**−16.5**
39	10	**−11.4**
40	10	**−15.4**
41	10	**−14.9**
42	10	**−19.1**
43	19	**−63.9**
44	18	**−55.3**
45	19	**−52.1**
46	19	**−59.5**
47	19	**−56.5**
48	19	**−61.4**

Supplementary Table 9 | The calculated potential energies (kcal mol^{-1}) of the [3]catenane **[3]CMM^{13+}** with the CBPQT^{4+} rings A and B moving on the loop along the hypothetical path O3 from point II to I′

Path O3		
Position of CBPQT^{4+} Ring A	Position of CBPQT^{4+} Ring B	**Potential Energy (kcal mol^{-1})**
28	12	**−3.3**
28	11	**−15.3**
27	10	**−14.2**
25	9	**−4.0**
25	8	**−13.4**
25	7	**−10.0**
25	6	**−7.4**
25	5	**−20.6**
25	4	**−23.3**
25	3	**−19.6**
25	2	**−6.9**
25	1	**−3.5**
25	0	**−27.3**
24	50	**−45.2**
24	49	**−42.5**
23	49	**−56.5**
22	48	**−56.5**
21	49	**−58.4**
20	49	**−55.9**
19	49	**−59.1**

Supplementary Table 10 | The calculated potential energies (kcal mol^{-1}) of the [3]catenane **[3]CMM^{13+}** with the CBPQT^{4+} rings A and B moving on the loop along the hypothetical path O4 from point II to I

Path O4		
Position of CBPQT^{4+} Ring A	Position of CBPQT^{4+} Ring B	**Potential Energy (kcal mol^{-1})**
28	12	**−3.3**
28	13	**5.3**
28	14	**7.8**
28	15	**17.9**
29	16	**25.4**
29	17	**24.1**
32	18	**19.8**
33	18	**23.8**
34	18	**19.9**
35	18	**14.6**
36	18	**16.9**
37	18	**8.1**
38	18	**−4.4**
39	18	**−14.0**
40	18	**−51.5**
41	18	**−52.9**
42	18	**−43.6**
43	18	**−44.4**
44	18	**−52.4**
45	18	**−53.2**
46	18	**−52.6**
47	18	**−50.7**
48	18	**−52.9**

7. X-Ray Crystallography

Crystallization Procedure: A 0.2 mM MeCN solution of the [3]catenane **[3]CMM**•13PF_6 was reduced by Zn dust to generate **[3]CMM$^{7+6•}$** before being filtered through a Pall syringe filter (pore size 0.45 μm) into VWR culture tubes (6 × 50 mm), followed by the addition of an excess of sodium trifluoromethanesulfonate into the solution. The tubes were allowed to stand at room temperature in a closed scintillation vial containing Et_2O (3 mL). After one-week, black purple block crystals appeared in the tubes. A suitable single crystal was selected and it was mounted on a MITIGEN holder with Paratone oil on a Bruker APEX-II CCD diffractometer. The crystal was kept at 100.01 K during data collection. Using Olex2[11], the structure was solved by employing the ShelXT[12] structure solution program using Intrinsic Phasing and refined with the XL[13] refinement package using Least Squares minimization.

Crystal Data for [(**[3]CMM$^{7+6•}$**)$_2$•(CF_3O_3S)$_9$•(PF_6)$_5$]•(MeCN)$_4$ (M = 6249.88): triclinic, space group $P\bar{1}$(no. 2), a / b / c = 19.268(2) / 20.918(3) / 22.183(3) Å, α / β / γ = 94.238(9) / 113.062(8) / 98.518(8)°, V = 8049.6(18) Å^3, Z = 1, T = 100.01 K, μ(CuKα) = 1.661 mm^{-1}, $Dcalc$ = 1.289 g/mm^3, 30014 reflections measured (4.316 ≤ 2Θ ≤ 100.894), 16615 unique (R_{int} = 0.0778, R_{sigma} = 0.1084) which were used in all calculations. The final R_1 was 0.2143 ($I > 2\sigma(I)$) and wR_2 was 0.5574 (all data).

Refinement Details. Distance restraints were imposed on the six-carbon methylene chain and the disordered anions. The enhanced rigid-bond restraint (SHELX keyword RIGU) was applied[14] globally. Restraints of similar amplitudes separated by less than 1.7 Å were also imposed globally. Equal displacement parameters were imposed on F atoms in the PF_6^- anion.

Solvent Treatment Details. The solvent masking procedure as implemented in Olex2 was used to remove the electronic contribution of solvent molecules from the refinement. As the exact solvent content is not known, only the atoms used in the refinement model are reported in the formula. Total solvent accessible volume / cell = 935.3 Å^3 [11.6%]. Total electron count / cell = 291.4.

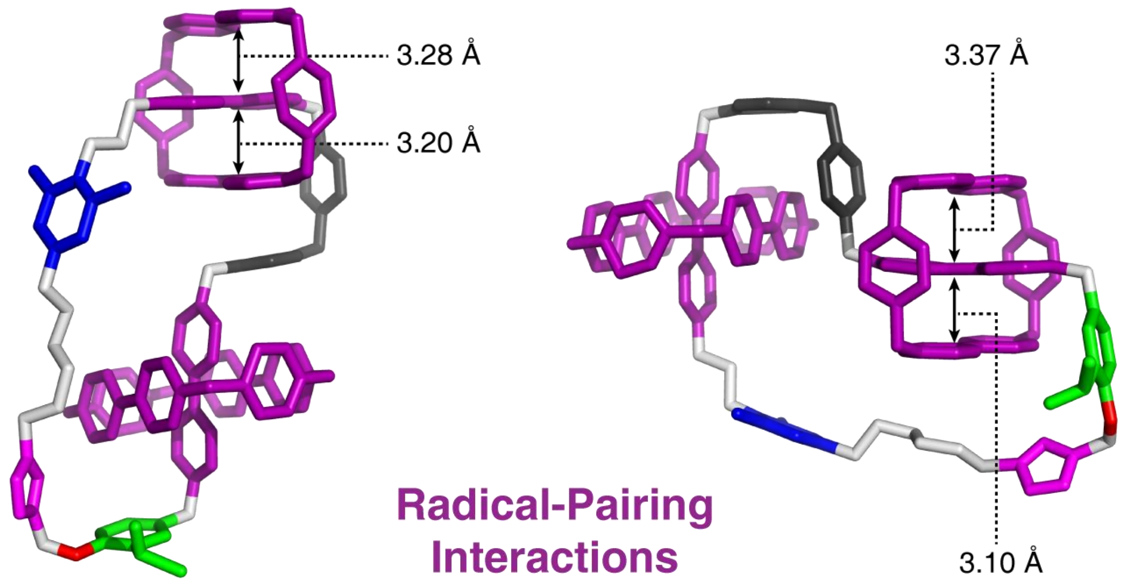

Supplementary Fig. 44 | Stick representations of the X-ray crystal structure of **[3]CMM$^{7+6•}$**. The annotated plane-to-plane distances are those between planes defined by the adjacent $V^{+•}$ units, showing the radical-pairing interactions. Hydrogen atoms and counterions were removed for the sake of clarity.

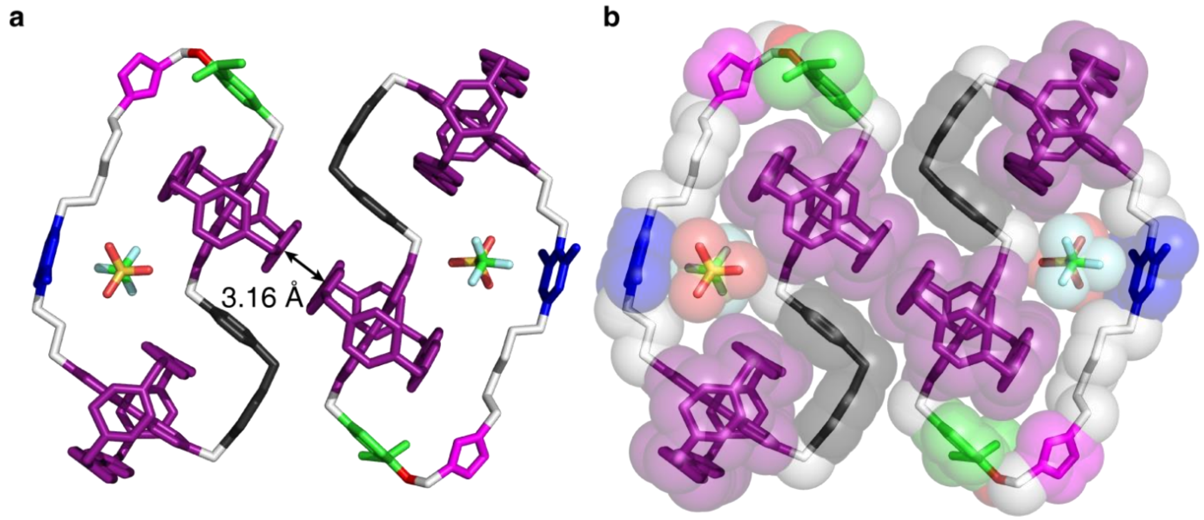

Supplementary Fig. 45 | a and **b**, Stick and superimposed space-filling representations of the X-ray crystal superstructures of **[3]CMM$^{7+6•}$**. The annotated plane-to-plane distance is that between adjacent CBPQT$^{2(+•)}$ rings, suggesting the presence of intermolecular radical-radical interactions. Hydrogen atoms and counterions were removed for the sake of clarity, except for two $CF_3SO_3^-$ counterions occupying the cavity of adjacent [3]catenanes **[3]CMM$^{7+6•}$**.

Supplementary Table 11 | Crystal data and structure refinement for **[3]CMM**$^{7+6•}$

Identification code	[(**[3]CMM**$^{7+6•}$)$_2$•(CF_3O_3S)$_9$•(PF_6)$_5$]•(MeCN)$_4$
Empirical formula	$C_{289}H_{282}F_{57}N_{36}O_{29}P_5S_9$
Formula weight	6249.88
Temperature / K	100.01
Crystal system	triclinic
Space group	$P\bar{1}$
a / Å, b / Å, c / Å	19.268(2), 20.918(3), 22.183(3)
α/°, β/°, γ/°	94.238(9), 113.062(8), 98.518(8)
Volume / Å^3	8049.6(18)
Z	1
ρ_{calc} / mg mm^{-3}	1.289
μ / mm^{-1}	1.661
F(000)	3232
Crystal size / mm^3	0.148 × 0.115 × 0.022
2Θ range for data collection	4.316 to 100.894°
Index ranges	$-19 \le h \le 18$, $-20 \le k \le 19$, $-13 \le l \le 22$
Reflections collected	30014
Independent reflections	16615[R(int) = 0.0778]
Data / restraints / parameters	16615 / 4877 / 2186
Goodness-of-fit on F^2	1.874
Final R indexes [$I > 2\sigma(I)$]	R_1 = 0.2143, wR_2 = 0.5004
Final R indexes [all data]	R_1 = 0.2930, wR_2 = 0.5574
Largest diff. peak / hole / e Å^{-3}	1.729 / −0.854

8. Cyclic Voltammetry

In order to gain a better understanding of the electron transfer processes during the redox cycle undergone by the [3]catenane **[3]CMM^{13+}**, cyclic voltammetry (CV) was carried out at room temperature in N_2-purged acetonitrile (MeCN) solutions with a Gamry Multipurpose instrument (Reference 600) interfaced to a PC. A three-electrode system was used to record the data, in which the working electrode was a glassy carbon (0.071 cm^2), the counter electrode was a Pt wire, and the reference electrode was an Ag/AgCl electrode. The surface of working electrode was polished routinely with 0.05 μm alumina-water slurry on a felt surface immediately before use. Tetrabutylammonium hexafluorophosphate ($TBAPF_6$) was used as the supporting electrolyte at a concentration of 0.1 M. **[3]CMM**•13PF_6 (2 mg) was dissolved in 1 mL MeCN solution ($TBAPF_6$, 0.1 M), resulting in the sample with an analyte concentration of 0.5 mM.

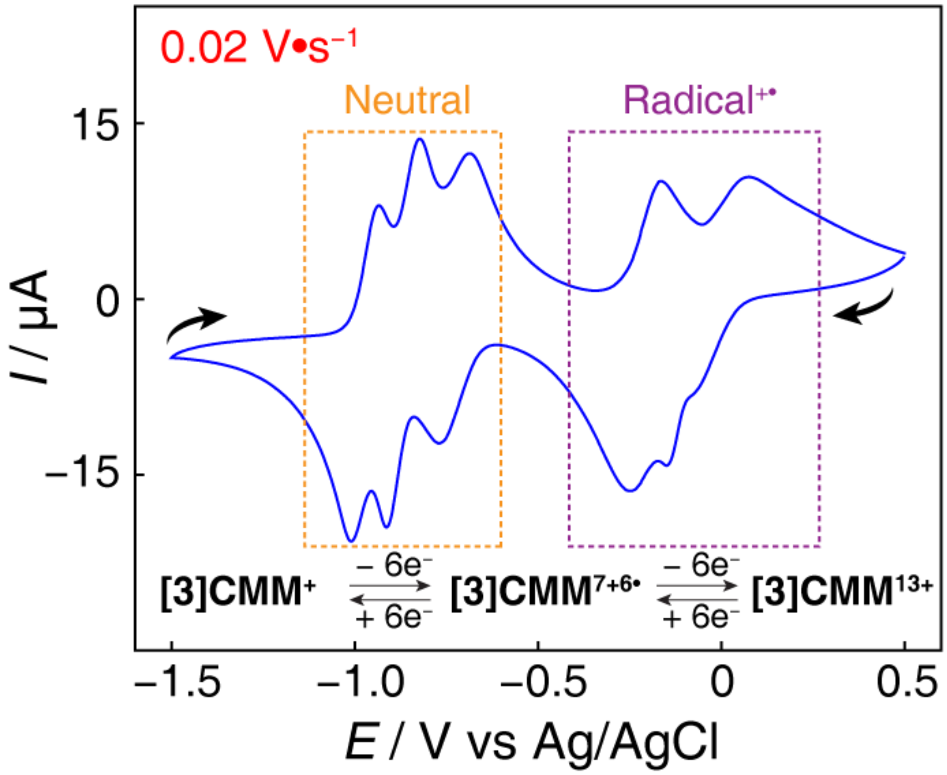

Supplementary Fig. 46 | Cyclic voltammograms of **[3]CMM**•13PF_6, scan rate / 0.02 V•s^{-1}

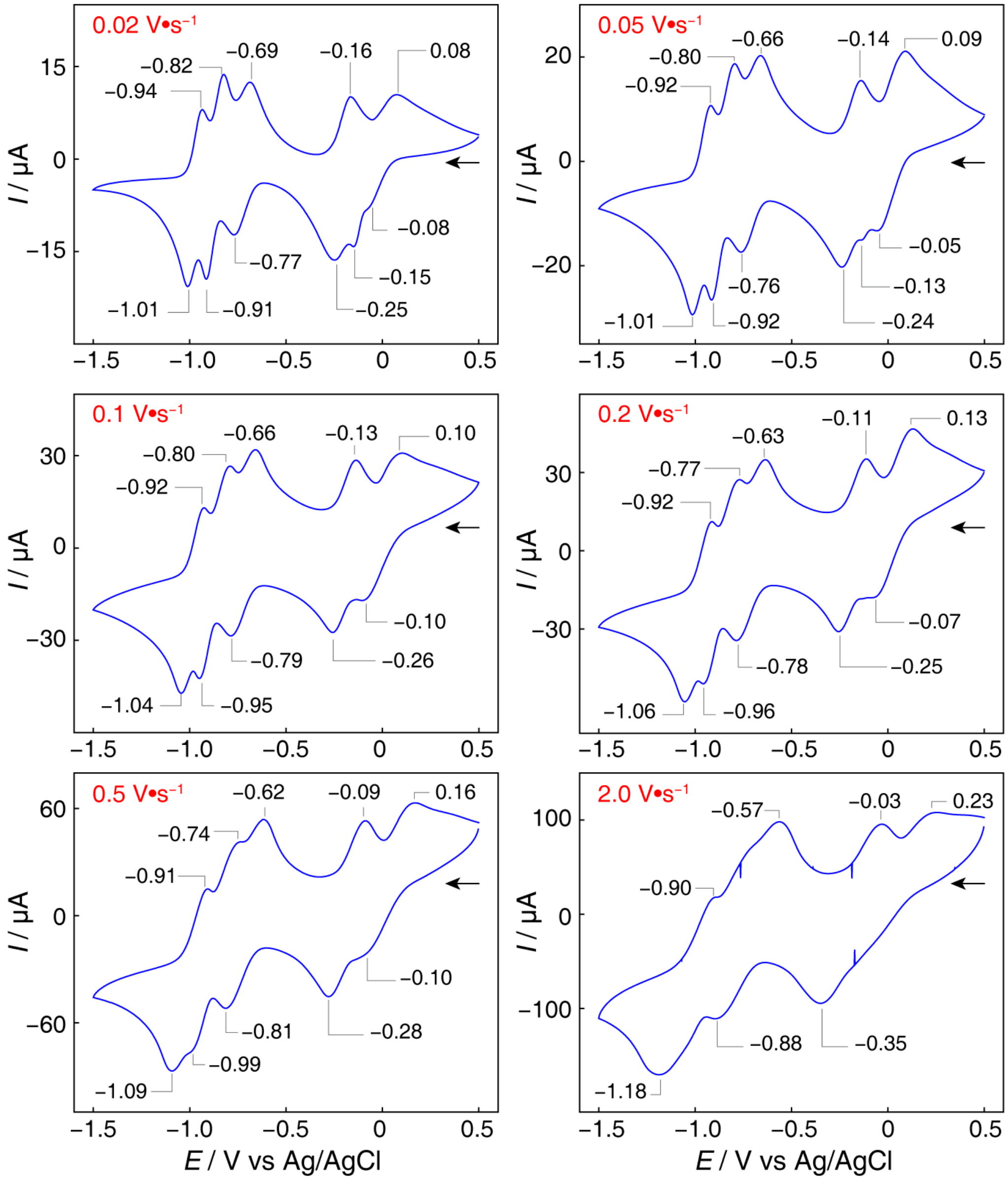

Supplementary Fig. 47 | Scan-rate variation (0.02 — 2.0 V•s^{-1}) of cyclic voltammograms of **[3]CMM**•13PF$_6$ (0.5 mM)

The CV profile displays (Supplementary Figs. 46 and 47) three reduction peaks with potentials at −0.08, −0.15, −0.25 V at a low scan rate (0.02 V•s^{-1}), corresponding to reduction associated with radical formation, starting from **[3]CMM^{13+}** and leading to the production of **[3]CMM$^{7+6•}$**. The first two reduction peaks (−0.08, −0.15 V) account for the stepwise formation of viologen radical pairs as a consequence of the different chemical environments experienced by the two CBPQT^{4+} rings in the [3]catenane **[3]CMM^{13+}**. The first reduction peak at −0.08 V can be assigned to the

reduction of one of the V^{2+} units in the loop and one of the two V^{2+} units in the $CBPQT^{4+}$ ring encircling the BPM unit, resulting in a decrease in Coulombic repulsion, while establishing stabilizing radical-pairing interactions between the mechanically interlocked components. The following reduction peak at −0.15 V can be attributed to the reduction of the other V^{2+} unit in the loop and one of the two V^{2+} units in the other $CBPQT^{4+}$ ring encircling the T unit. The third reduction peak observed at −0.25 V, corresponding to two simultaneous one-electron reductions, accounts for the further reduction of both $CBPQT^{2+(\bullet+)}$ monoradical trication rings to their diradical dicationic states $CBPQT^{2(\bullet+)}$. The oxidation of the radical state **[3]CMM$^{7+6\bullet}$** back to **[3]CMM^{13+}** occurs in two steps at −0.16 and +0.08 V. The first oxidation peak at −0.16 V can be assigned to two one-electron oxidations of the two unpaired $V^{+\bullet}$ units in the two $CBPQT^{(2+\bullet)}$ rings ($CBPQT^{2(+\bullet)}$ → $CBPQT^{2+(+\bullet)}$), resulting in much weaker binding interactions and increased Coulombic repulsion between the components ($V^{+\bullet}$ and $CBPQT^{2+(+\bullet)}$), i.e., **[3]CMM$^{7+6\bullet}$** is oxidized to **[3]CMM$^{5+4(+\bullet)}$**. It is followed by four simultaneous one-electron oxidations — namely, two $V^{+\bullet}$ units in the two $CBPQT^{2+(\bullet+)}$ rings and two $V^{\bullet+}$ in the loop are oxidized at the same oxidization potential (+0.08 V), resulting in the formation of the fully oxidized state **[3]CMM^{13+}**. These observations are consistent with previously published results[15].

The reduction of the radical state **[3]CMM$^{7+6\bullet}$** to its neutral (viologen) form **[3]CMM^{+}** involves (Supplementary Figs. 46 and 47) three sequential two-electron reversible processes. The first and less negative one (−0.77 V, peak potential) can be assigned to the reduction of the two unpaired $V^{+\bullet}$ units in the two $CBPQT^{2(+\bullet)}$ rings, leading to the formation of **[3]CMM$^{+4(+\bullet)}$**, which is stabilized[16] by both radical-pairing and donor-acceptor interactions. The following two-electron process accounts for the reduction of the other two $V^{+\bullet}$ units in the two $CBPQT^{\,(+\bullet)}$ rings. Finally, the reduction of the remaining two $V^{+\bullet}$ units in the loop occurs at −1.01 V, reflecting the presence of mechanical bonding and the nanoconfined geometry of the [3]catenane.

Variable scan-rate CV experiments (Supplementary Fig. 47) were also performed. As the scan rate is increased to 2.0 V•s^{-1}, only one reduction peak for the radical state **[3]CMM$^{7+6\bullet}$** is observed, indicating that the electron-transfer process is much faster than ring movement at this fast scan rate. This observation suggests that, under the experimental conditions employed during the operation of the [3]catenane motor, the reduction to the radical state **[3]CMM$^{7+6\bullet}$** and the re-oxidation to the fully oxidized state **[3]CMM^{13+}** is completed fully and very rapidly.

9. Vis/NIR Absorption Spectroscopy

The radical characteristics of **[3]CMM$^{7+6•}$** were investigated by Vis/NIR spectrophotometry. Treatment of the [3]catenane **[3]CMM^{13+}** in MeCN with 6 equiv of the one-electron reductant cobaltocene (Cp_2Co) resulted immediately in a characteristic color change from colorless to dark purple, indicating the formation of its radical state, namely **[3]CMM$^{7+6•}$**. The absorption spectra of a series of MeCN solutions, containing different concentrations of **[3]CMM$^{7+6•}$**, ranging from 5 – 40 μM, were recorded.

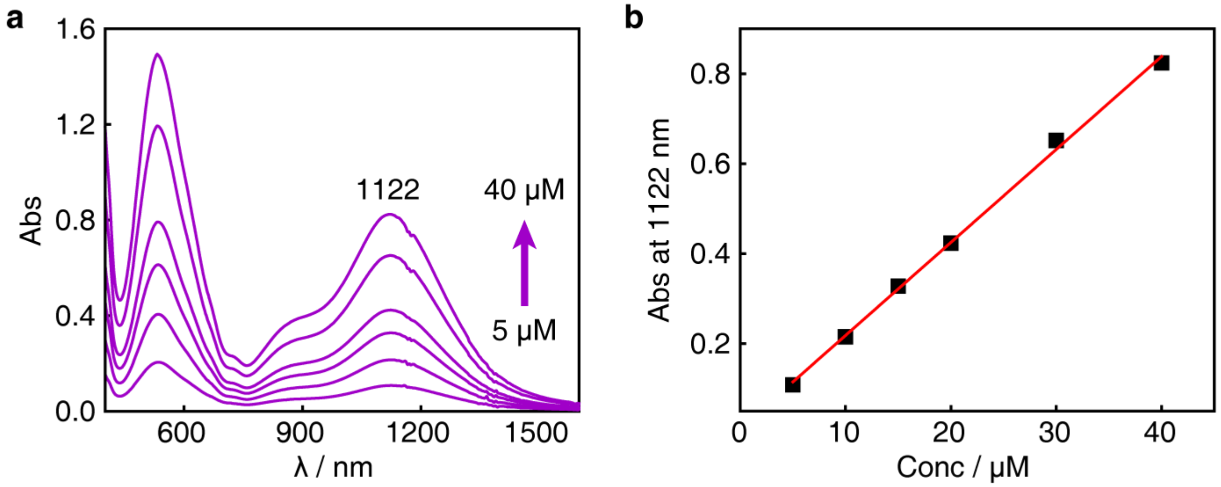

Supplementary Fig. 48 | a, Vis/NIR Spectra of the reduced [3]catenane **[3]CMM$^{7+6•}$** recorded over a range of different concentrations. **b**, Dependence of the intensity of the absorption band at 1122 nm for **[3]CMM$^{7+6•}$** on concentration. The linear relationship can be ascribed to the intramolecular trisradical interactions.

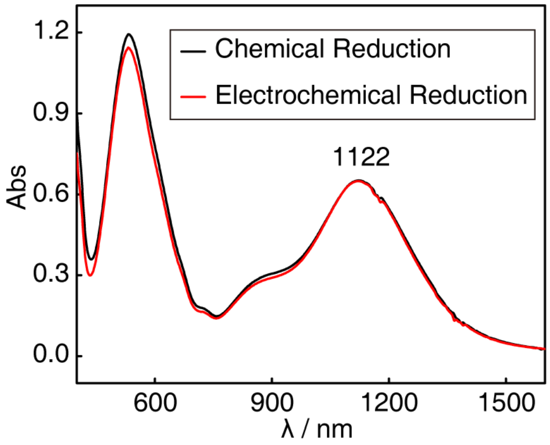

Supplementary Fig. 49 | Vis/NIR Spectra of the reduced [3]catenane **[3]CMM$^{7+6•}$** (30 μM) obtained by both chemical and electrochemical reduction.

10. Electrically Driven Operation of [3]CMM

Controlled Potential Electrolysis (CPE) Procedure: The CPE experiments were performed[17] inside a N_2-filled glovebox using a BASi® bulk electrolysis cell which was equipped with a reticular vitreous carbon (RVC) working electrode, a coiled platinum wire auxiliary electrode within a fritted glass chamber and a Ag/AgCl reference electrode, and connected to a Gamry multipurpose instrument (Reference 600) interfaced to a PC. The experimental parameters were controlled using the software of a Gamry Framework Version 6.30 operating in the chronocoulometry mode. In a typical experiment, **[3]CMM**•13PF_6 (4 mg) was dissolved in MeCN (30 mL, 0.1 M $TBAPF_6$) in the working cell, while the auxiliary electrode chamber was filled with ferrocene dissolved in MeCN (1 mL, 0.1 M $TBAPF_6$). The whole apparatus was subjected to one redox cycle with alternate constant potentials of –0.7 V (reduction potential vs Ag/AgCl) and +1.4 V (oxidation potential vs Ag/AgCl) for 10 min with no resting periods between reduction and oxidation. After this redox cycle, the solution in the working cell was transferred to a round-bottomed flask. Following removing all the solvent, the resulting solid was washed with CH_2Cl_2 to remove the excess of $TBAPF_6$. After drying under vacuum, the recovered **[3]CMM**•13PF_6 was characterized by ^{1}H NMR spectroscopy.

Repeated CPE Procedure: In the repeated controlled potential electrolysis experiment, we optimized[18] (Supplementary Fig. 50) the electrochemical set-up, in which the auxiliary electrode was composed of a platinum wire wrapped with a copper wire instead of using ferrocene. In order to limit the degradation of the [3]catenane, we used a less negative reduction potential (−0.5 V) and a less positive oxidation potential (+0.7 V). Accordingly, the time for the oxidation step was extended from 10 to 15 min. An MeCN (38 mL, 0.1 M $TBAPF_6$) solution of **[3]CMM**•13PF_6 (30 μM) was added to the working cell, while the auxiliary electrode chamber was filled with an excess

of $Cu(MeCN)_4PF_6$ dissolved in MeCN (1 mL, 0.1 M $TBAPF_6$). The auxiliary electrode was constituted of a platinum wire wrapped with a copper wire (diam. 0.25 mm, 99.999% trace metals basis from Sigma Aldrich). The whole apparatus was subjected to five redox cycles with alternate constant potentials of –0.5 V (reduction potential vs Ag/AgCl) and +0.7 V (oxidation potential vs Ag/AgCl) for 10 and 15 min, respectively. After each reduction/oxidation, 1 mL of solution was drawn out from the working cell for further analysis by Vis/NIR spectroscopy. After five redox cycles, the ^{1}H NMR spectrum (Supplementary Fig. 51) of the re-oxidized species, recorded after removing the excess of $TBAPF_6$ by washed with CH_2Cl_2, shows a recovery of more than 95% of **[3]CMM**•$13PF_6$ with a minimal amount (5%) of degradation when compared with the initial spectrum.

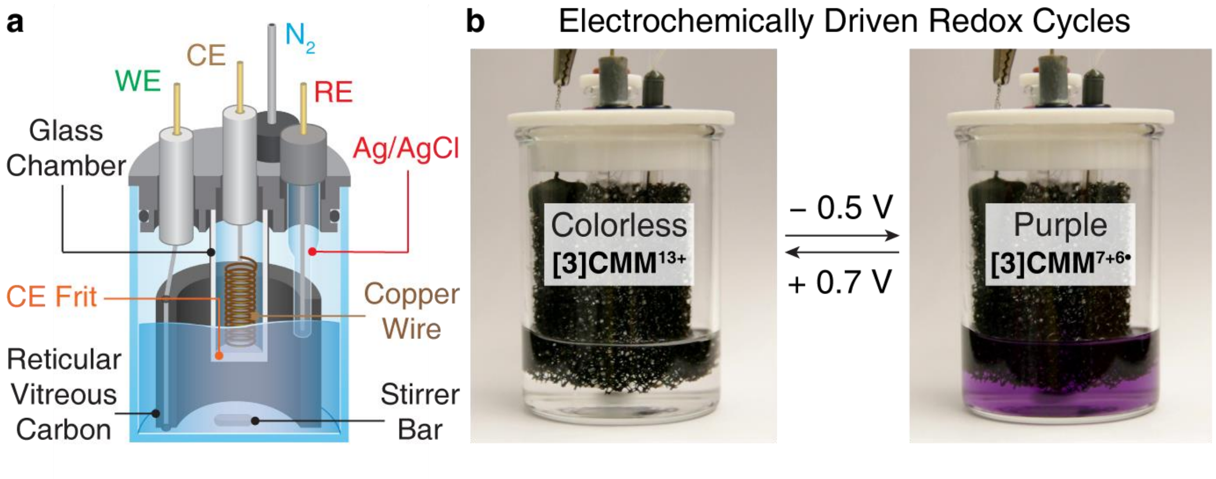

Supplementary Fig. 50 | a, Graphical illustration of the electrochemical cell used in the repeated controlled potential electrolysis experiments. Working Electrode, WE; Counter Electrode, CE; Reference Electrode, RE. **b**, Photographs of the oxidized state (Left) **[3]CMM^{13+}** and the reduced state (Right) **[3]CMM$^{7+6•}$** in the electrochemical cell

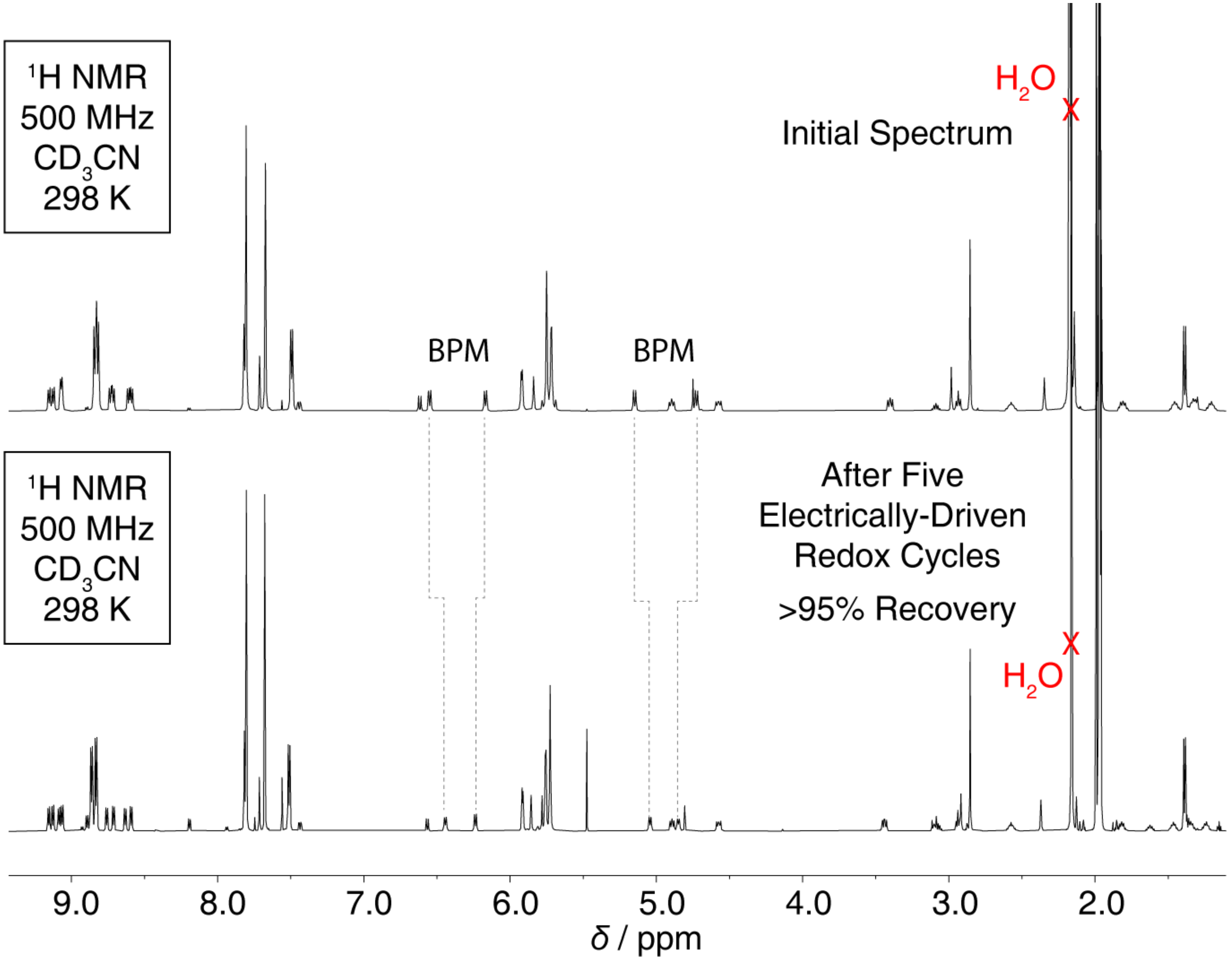

Supplementary Fig. 51 | ^{1}H NMR Spectra (500 MHz, CD_3CN, 298 K) of **[3]CMM**•13PF_6. Bottom: Initial spectrum. Top: After five electrically driven redox cycles. On account of the different water contents in CD_3CN, there are subtle differences between the two ^{1}H NMR spectra.

11. Chemically Driven Operation of [3]CMM

Reversible switching between the fully oxidized **[3]CMM^{13+}** and the reduced **[3]CMM$^{7+6•}$** states using chemical stimuli followed by Vis/NIR spectrophotometry. The switching between the two states was repeated five times, starting with **[3]CMM$^{7+6•}$**. The reduced state **[3]CMM$^{7+6•}$** was obtained by treated with 6 equiv Cp_2Co, and the re-oxidization of the **[3]CMM$^{7+6•}$** back to **[3]CMM^{13+}** was achieved by using 6 equiv $NOPF_6$ to complete one cycle.

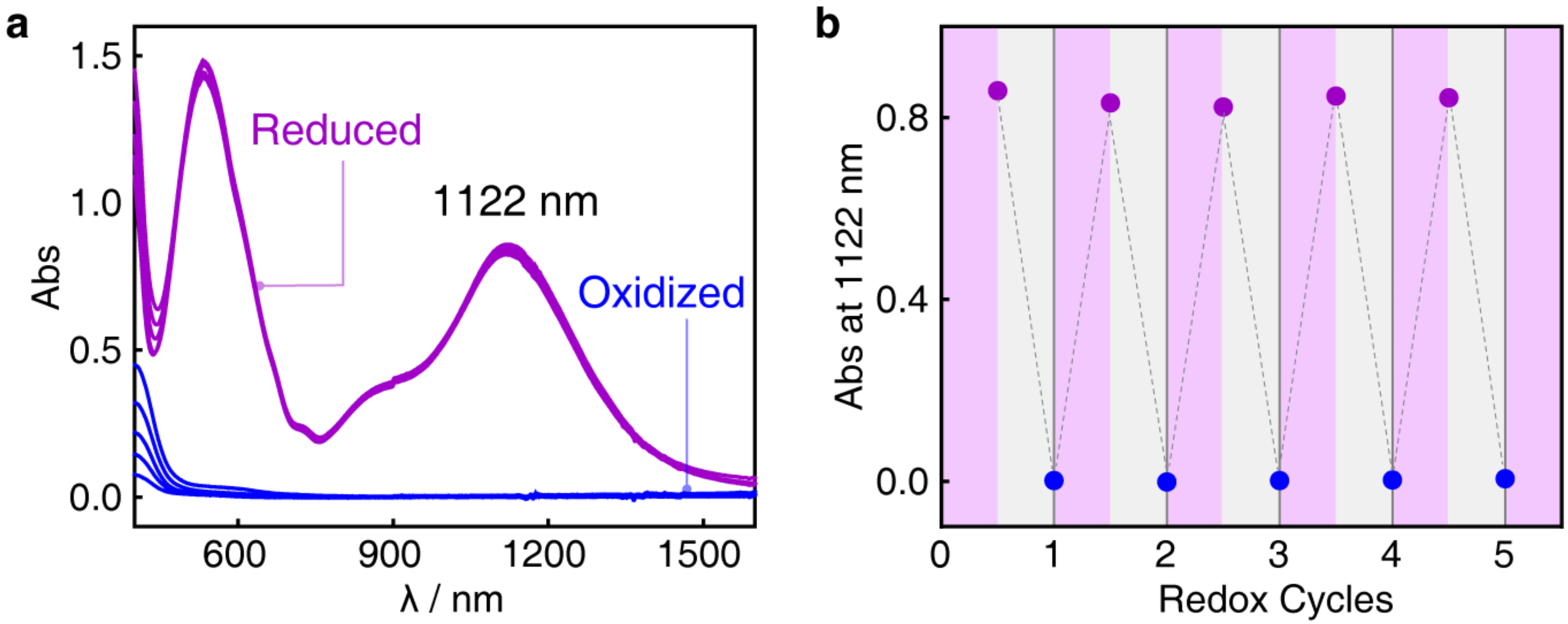

Supplementary Fig. 52 | a, Vis/NIR Spectra (40 µM) of the reduced state **[3]CMM$^{7+6•}$** and the oxidized state **[3]CMM^{13+}**. **b**, Absorption intensities of **[3]CMM$^{7+6•}$** (purple) and **[3]CMM^{13+}** (blue) at 1122 nm wavelength showing the reversible switching between the two states during each cycle

12. Measurements of the Directionality

Supplementary Scheme 7 | Synthesis of Deuterium-Labeled [D_{16}]-**CBPQT•**$4PF_6$

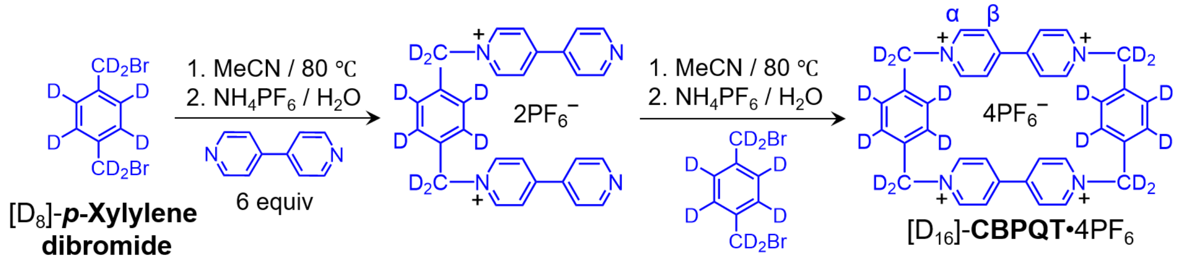

[D_{16}]-CBPQT•$4PF_6$: Bromination of [D_{10}]-*p*-xylylene (Cambridge) with *N*-bromosuccinimide gave [D_8]-*p*-xylylene dibromide, yield 60%. [D_{16}]-**CBPQT**•$4PF_6$ was prepared according to the same procedure for **CBPQT**•$4PF_6$ by using [D_8]-*p*-xylylene dibromide instead of *p*-xylylene dibromide (18% yield after two steps).

^{1}H NMR (500 MHz, CD_3CN) δ 8.87 (d, J = 7.0 Hz, 8H, H_α), 8.16 (d, J = 7.0 Hz, 2H, H_β). **^{13}C NMR** (125 MHz, CD_3CN) δ 150.31, 146.18, 136.71, 128.26. **HRMS-ESI** (*m/z*): calcd. for [$C_{36}H_{16}D_{16}F_{24}N_4OP_4$ – $2PF_6$]$^{2+}$ 413.1452, found 413.1477; calcd. for [$C_{36}H_{16}D_{16}F_{24}N_4OP_4$ – PF_6]$^+$ 971.2551, found 971.2579.

Supplementary Scheme 8 | Synthesis of Deuterium-Labeled [D_{32}]-**[3]CMM•**$13PF_6$

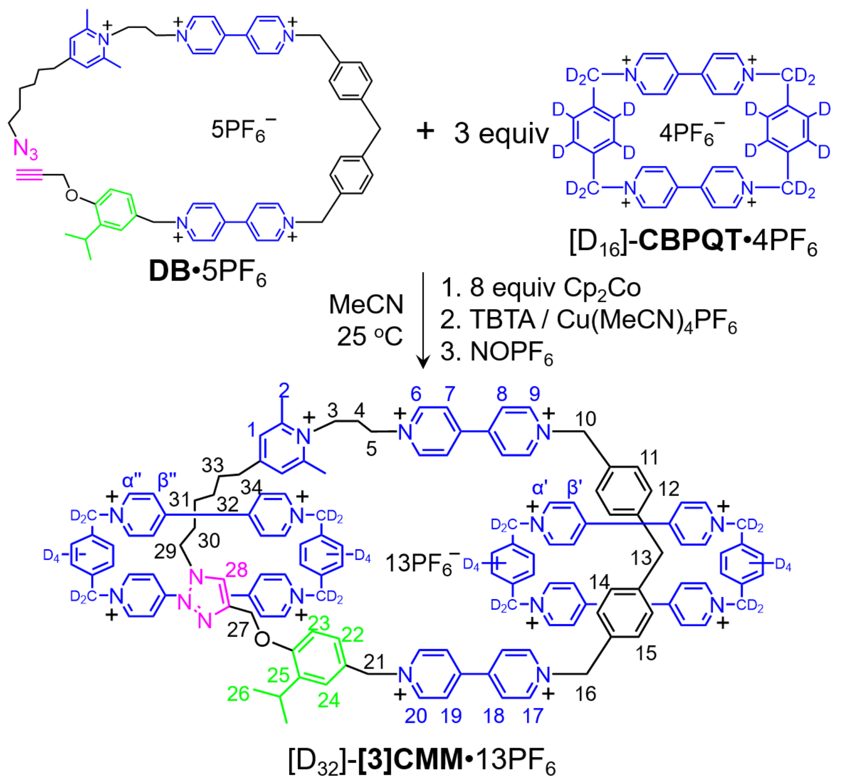

$[D_{32}]$-**[3]CMM**•13PF_6 was prepared according to the same procedure (**Supplementary Scheme 6**) employed in the preparation of **[3]CMM**•13PF_6 by using $[D_{16}]$-**CBPQT**•4PF_6 instead of **CBPQT**•4PF_6. **^{1}H NMR** (500 MHz, CD_3CN) δ 9.12 (d, J = 7.0 Hz, 2H, H_9), 9.09 (d, J = 7.0 Hz, 2H, H_{20}), 9.06 – 9.03 (m, 4H, $H_{6,17}$), 8.82 (d, J = 6.5 Hz, 8H, $H_{\alpha'}$), 8.79 (d, J = 6.5 Hz, 8H, $H_{\alpha''}$), 8.72 (d, J = 6.5 Hz, 2H, H_8), 8.68 (d, J = 6.5 Hz, 2H, H_{18}), 8.58 (d, J = 7.0 Hz, 2H, H_{19}), 8.56 (d, J = 6.5 Hz, 2H, H_7), 7.78 (d, J = 7.0 Hz, 8H, $H_{\beta''}$), 7.68 (s, 2H, H_1), 7.65 (d, J = 7.5 Hz, 1H, H_{24}), 7.47 (d, J = 7.0 Hz, 8H, $H_{\beta'}$), 7.41 (dd, J = 8.5, 2.0 Hz, 1H, H_{22}), 6.58 (d, J = 8.5 Hz, 1H, H_{23}), 6.45 (d, J = 8.0 Hz, 2H, H_{11}), 6.16 (d, J = 8.0 Hz, 2H, H_{15}), 5.89 (s, 4H, $H_{10,21}$), 5.82 (s, 2H, H_{16}), 5.05 (d, J = 7.5 Hz, 2H, H_{12}), 4.86 (t, J = 8.0 Hz, 2H, H_5), 4.76 (d, J = 7.5 Hz, 2H, H_{14}), 4.69 (s, 1H, H_{28}), 4.55 (t, J = 7.5 Hz, 2H, H_3), 3.37 (t, J = 8.0 Hz, 2H, H_{34}), 3.06 (quint, J = 7.0 Hz, 1H, H_{25}), 2.93 (s, 2H, H_{27}), 2.91 (t, J = 7.0 Hz, 2H, H_{29}), 2.83 (s, 6H, H_2), 2.57 – 2.51 (m, 2H, H_4), 2.33 (s, 2H, H_{13}), 1.78 (quint, J = 7.0 Hz, 2H, H_{30}), 1.43 (quint, J = 7.0 Hz, 2H, H_{31}), 1.36 (d, J = 6.5 Hz, 6H, H_{26}), 1.32 – 1.27 (m, 2H, H_{32}), 1.21 – 1.15 (m, 2H, H_{33}). **HRMS-ESI** (*m/z*): calcd. for $[C_{136}H_{103}D_{32}F_{78}N_{16}OP_{13} - 2PF_6]^{2+}$ 1817.9548, found 1817.9547; calcd. for $[C_{136}H_{103}D_{32}F_{78}N_{16}OP_{13} - 3PF_6]^{3+}$ 1163.6483, found 1163.6485.

Supplementary Scheme 9 | Synthesis of [D$_n$]**-[3]CMM•**13PF$_6$

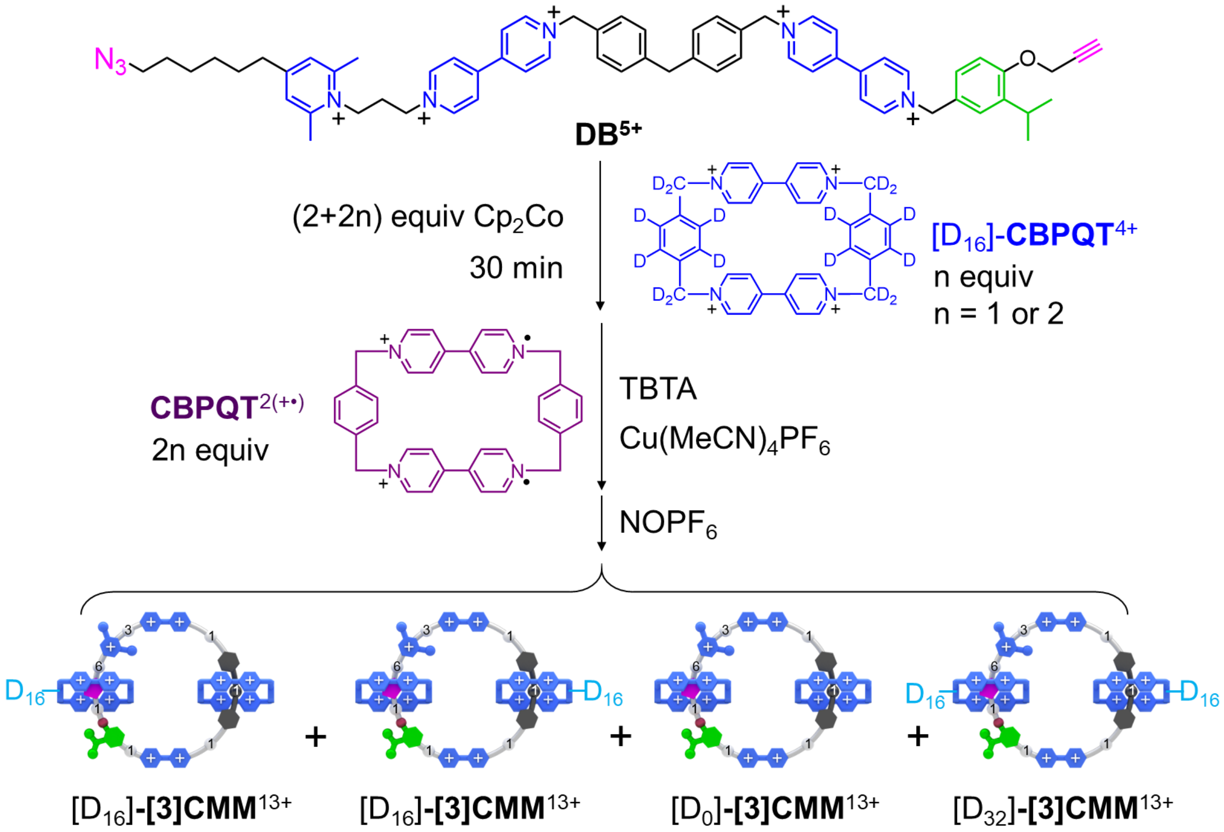

[D$_n$]**-[3]CMM**•13PF$_6$ (n = 0, 16, 32) was prepared according to the same procedure (**Supplementary Scheme 6**) employed in the synthesis of **[3]CMM•**13PF$_6$, except [D$_{16}$]-**CBPQT^{4+}** and **CBPQT^{4+}** were added (**Supplementary Scheme 9**) in sequence during the radical template-directed synthesis.

Note: Depending on the different equivs (1 or 2) of [D$_{16}$]-**CBPQT^{4+}** added during the synthesis, we observed batch variations with regard to the ratio of each isotopologues, according to ^{1}H NMR spectroscopic analyses.

HRMS-ESI (*m/z*): [D$_0$]-**[3]CMM•**13PF$_6$, calcd. for [$C_{136}H_{135}F_{78}N_{16}OP_{13}$ − 3PF$_6$]$^{3+}$ 1152.9147, found 1152.9157; [D$_{16}$]-**[3]CMM•**13PF$_6$, calcd. for [$C_{136}H_{119}D_{16}F_{78}N_{16}OP_{13}$ − 3PF$_6$]$^{3+}$ 1158.2815, found 1158.2838; [D$_{32}$]-**[3]CMM•**13PF$_6$, calcd. for [$C_{136}H_{103}D_{32}F_{78}N_{16}OP_{13}$ − 3PF$_6$]$^{3+}$ 1163.6483, found 1163.6490.

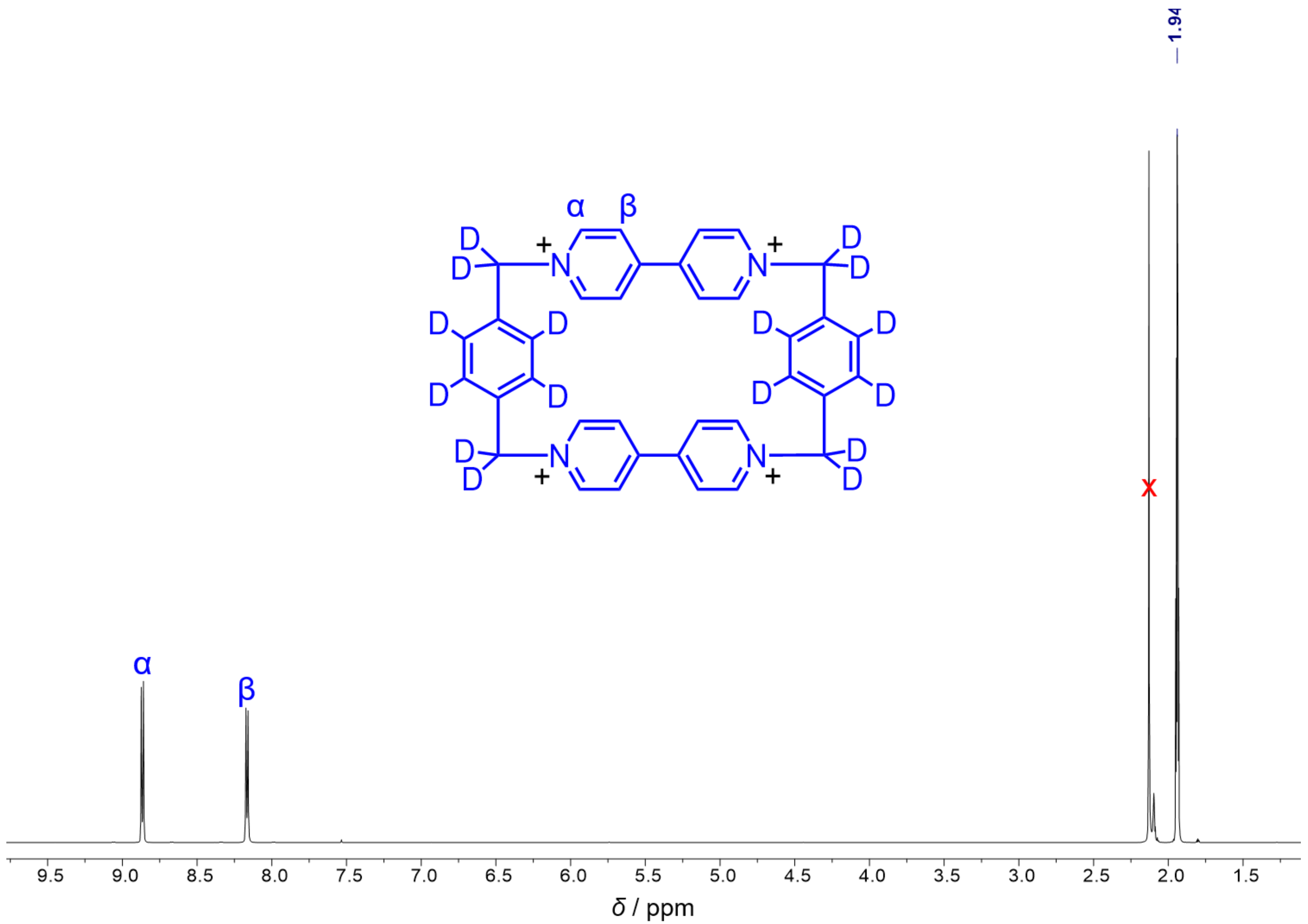

Supplementary Fig. 53 | ^{1}H NMR Spectrum (500 MHz, CD_3CN, 298 K) of [D_{16}]-**CBPQT**•$4PF_6$

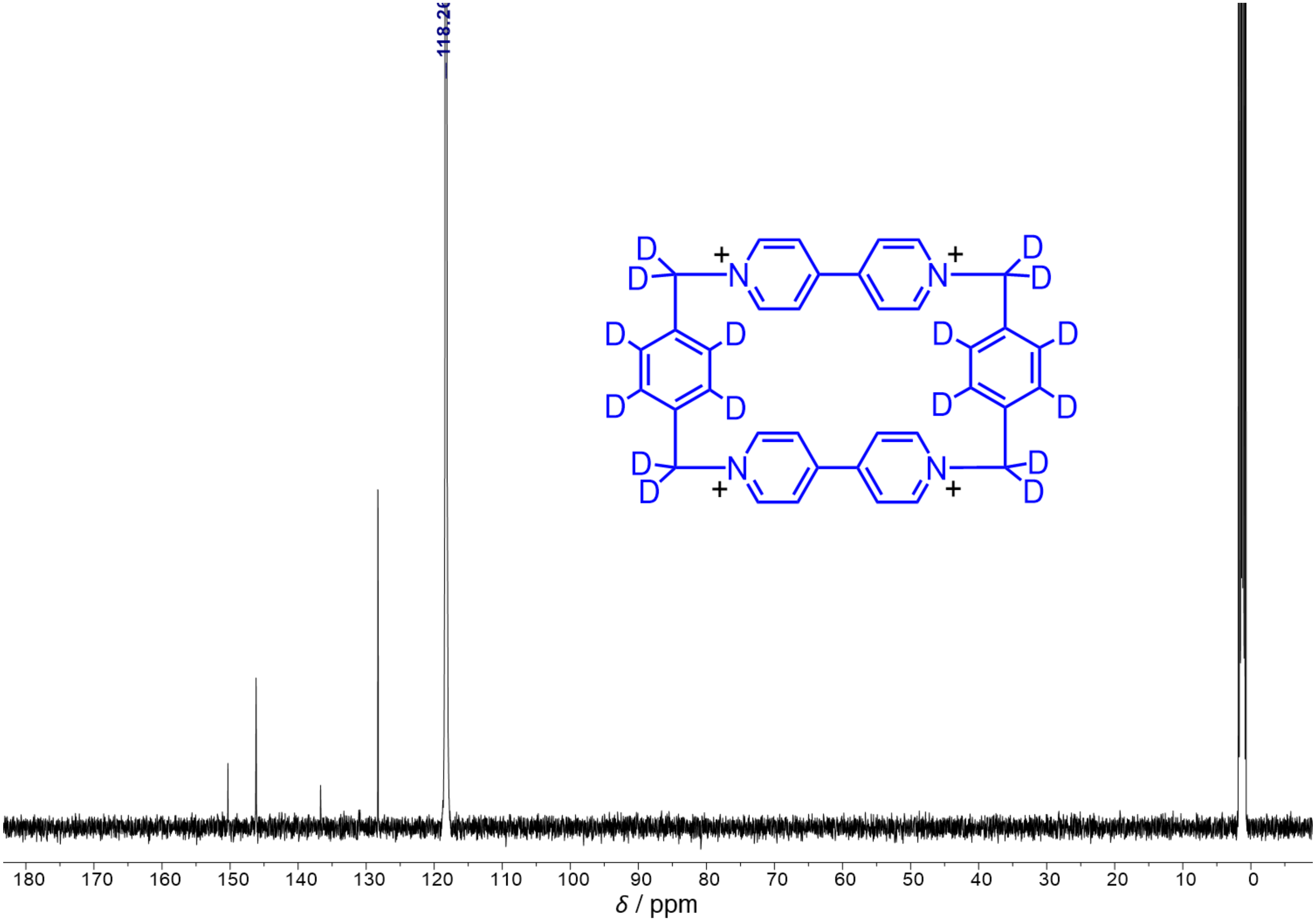

Supplementary Fig. 54 | ^{13}C NMR Spectrum (125 MHz, CD_3CN, 298 K) of [D_{16}]-**CBPQT**•$4PF_6$

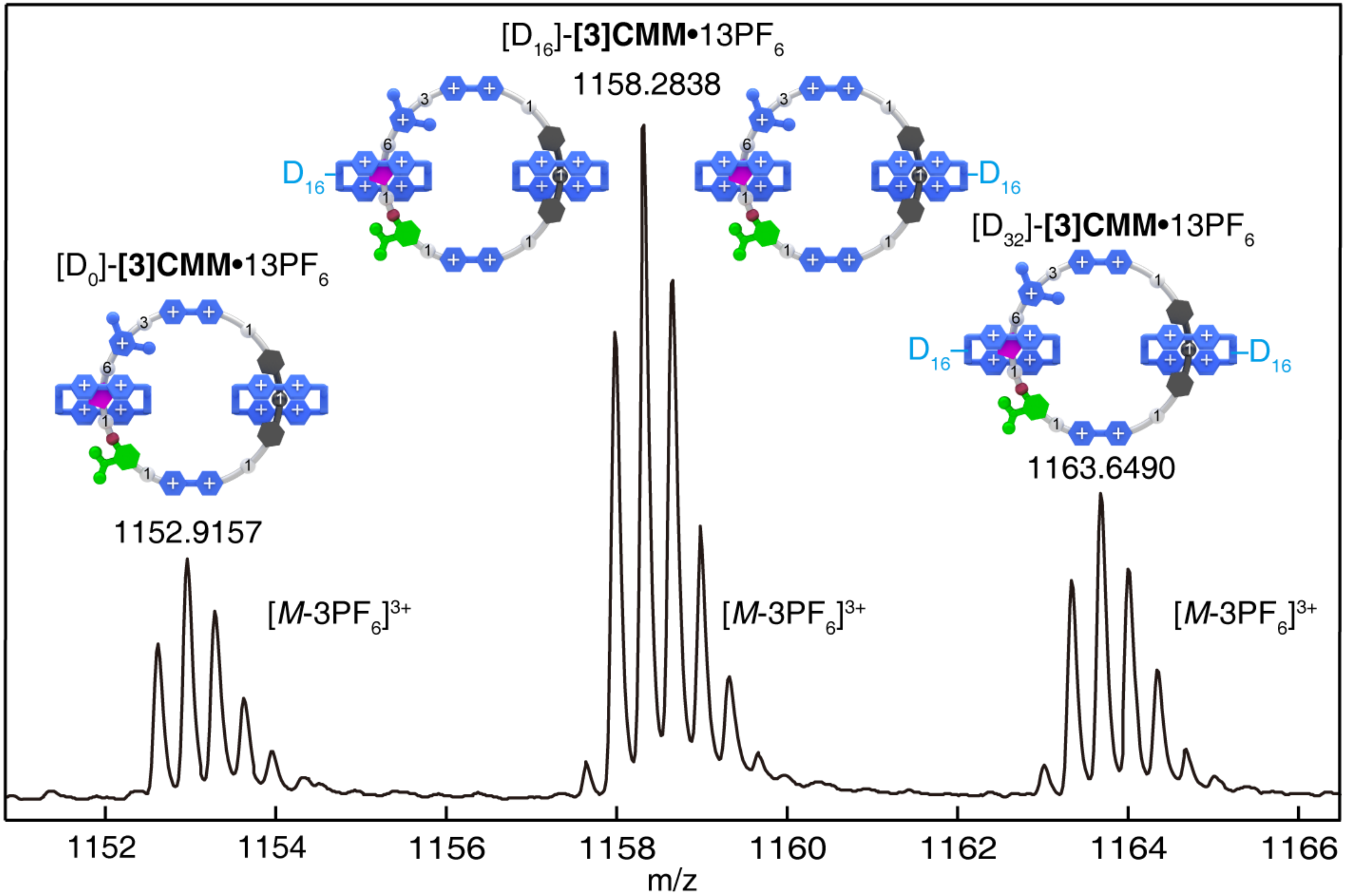

Supplementary Fig. 55 | HRMS-ESI Spectra of [D$_n$]-**[3]CMM**•13PF$_6$

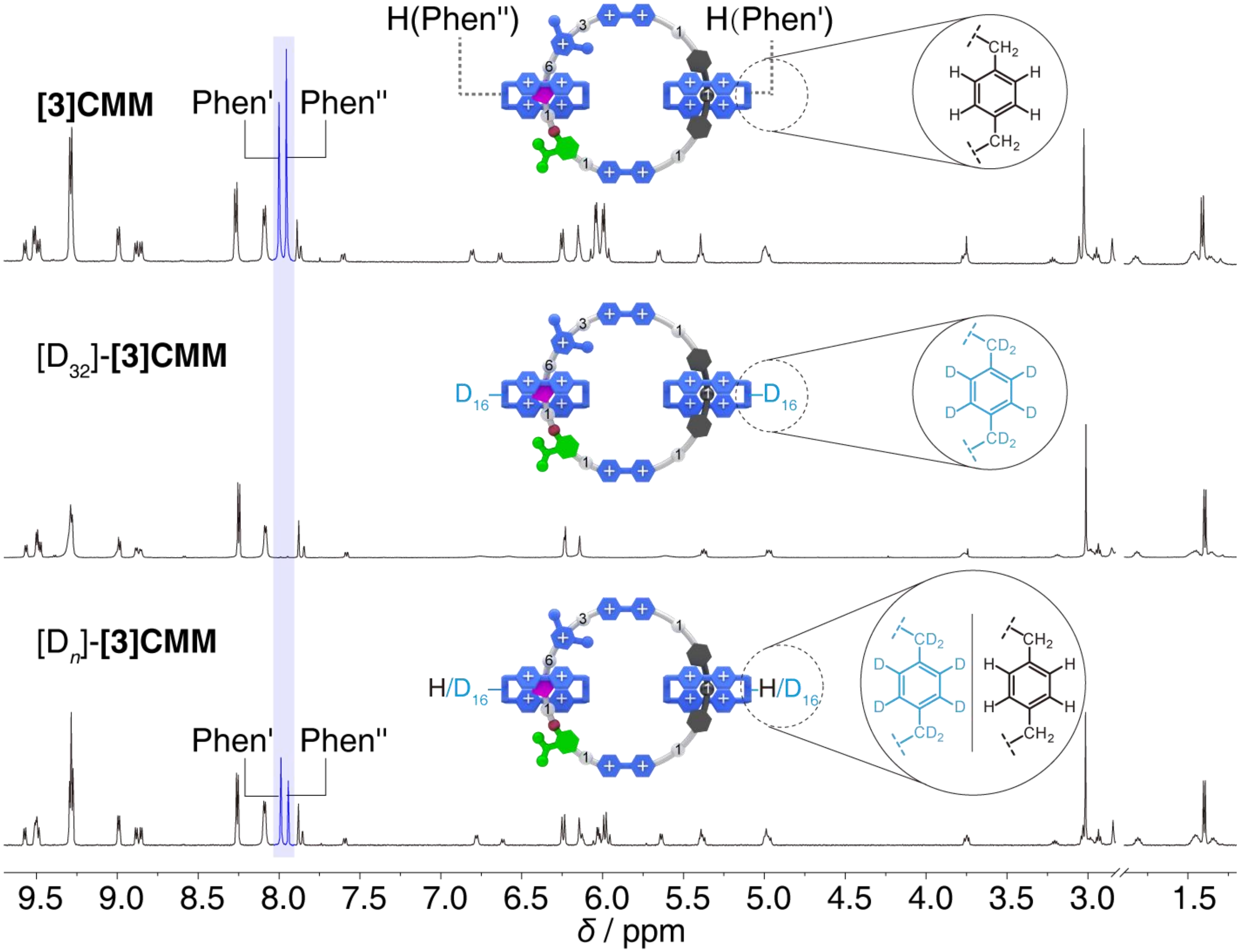

Supplementary Fig. 56 | Stacked ^{1}H NMR spectra (500 MHz, CD_3COCD_3, 298 K) of **[3]CMM**•13PF_6 (top), [D_{32}]-**[3]CMM**•13PF_6 (middle), and [D_n]-**[3]CMM**•13PF_6 (bottom)

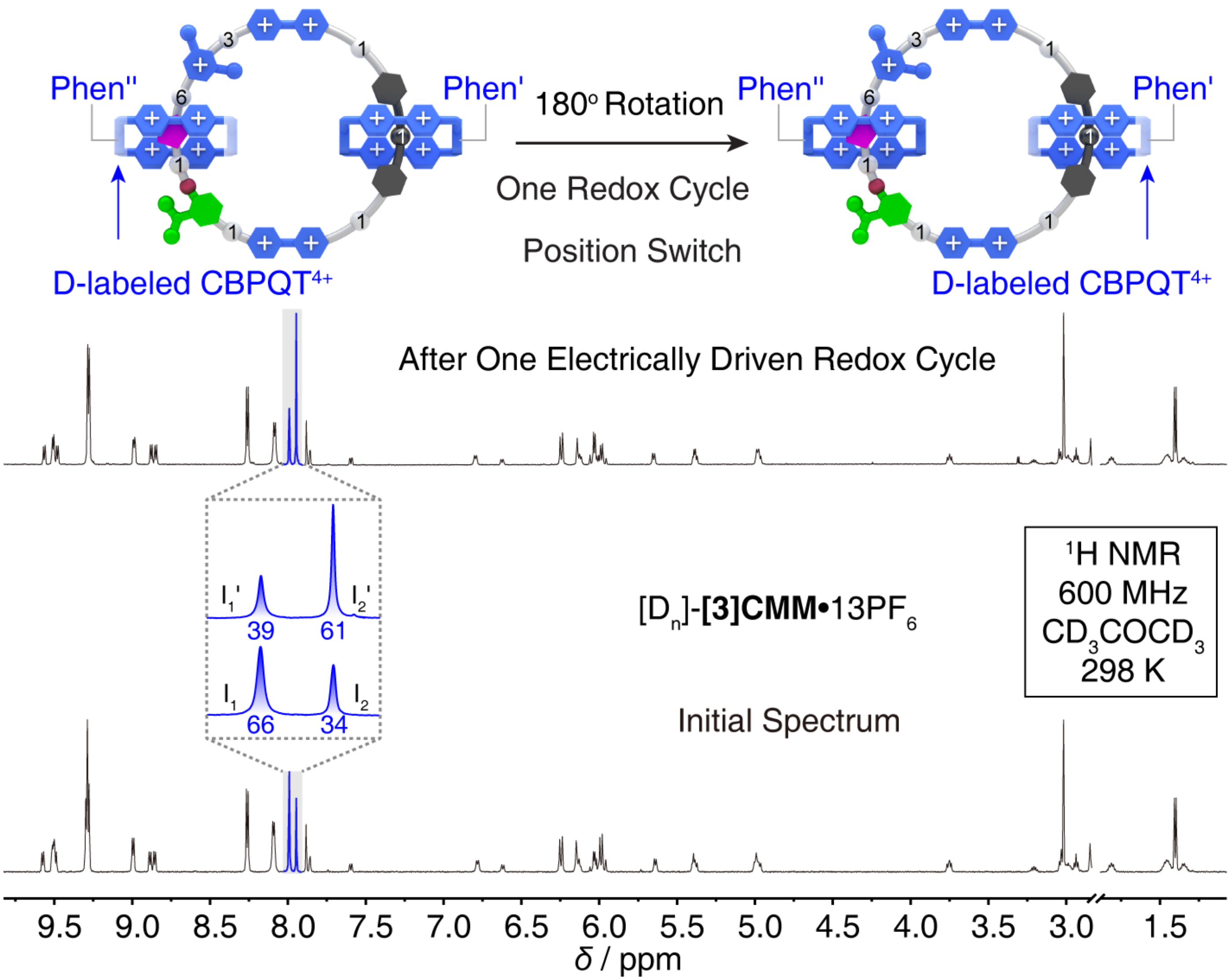

Supplementary Fig. 57 | ^{1}H NMR Spectra (600 MHz, CD_3COCD_3, 298 K) of [D_n]-**[3]CMM**•13PF_6. Bottom: Initial spectrum. Top: After one redox cycle of electrically driven operation. Conditions: [D_n]-**[3]CMM**•13PF_6 (4 mg) in MeCN (30 mL, 0.1 M $TBAPF_6$), reduction potential at −0.7 V (vs Ag/AgCl) for 10 min, oxidation potential at +1.4 V (vs Ag/AgCl) for 10 min

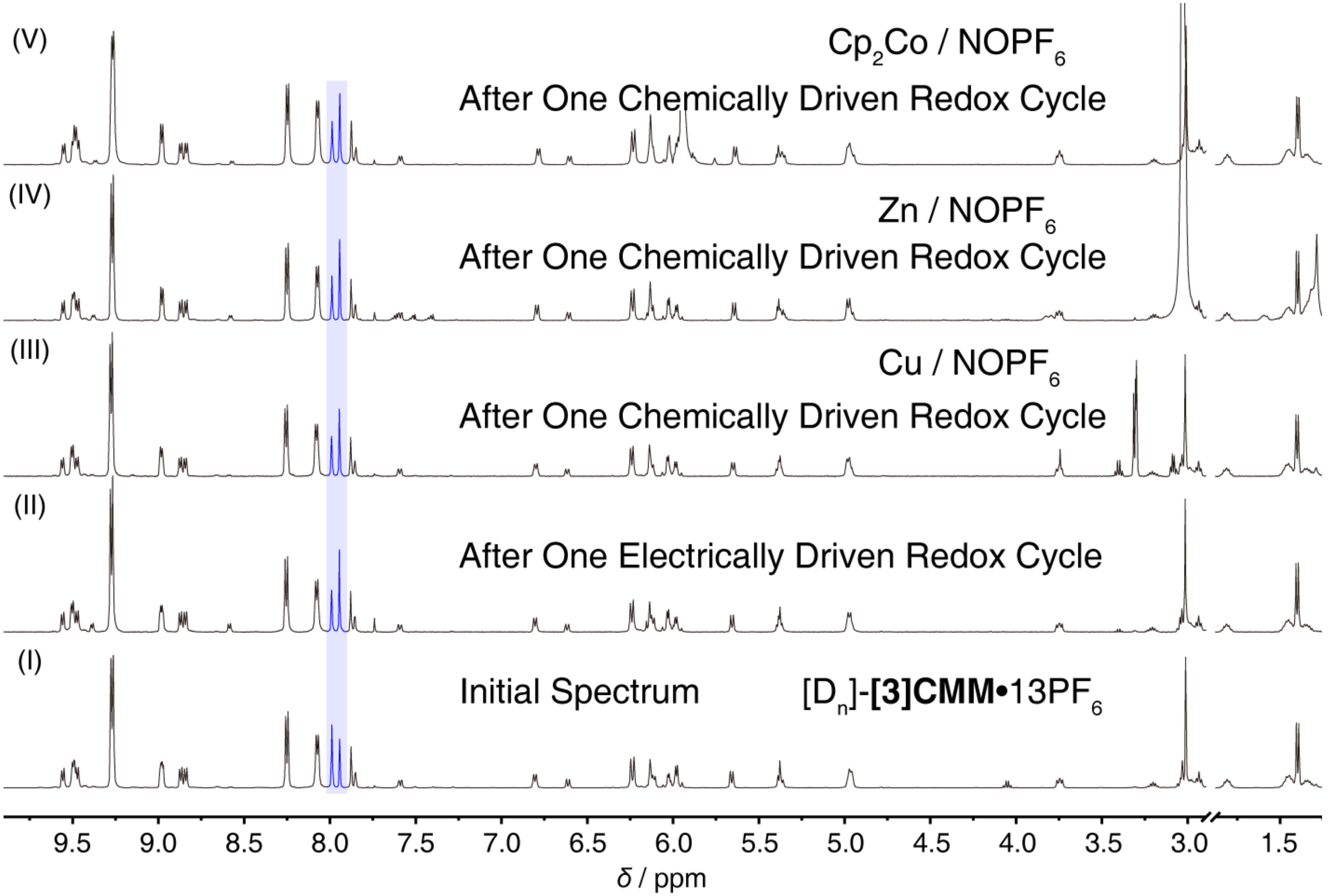

Supplementary Fig. 58 | ^{1}H NMR Spectra (600 MHz, CD_3COCD_3, 298 K) of [D_n]-**[3]CMM•**13PF_6. (I) Initial spectrum; (II) after one redox cycle of electrically driven operation. Conditions: [D_n]-**[3]CMM•**13PF_6 (4 mg) in MeCN (30 mL, 0.1 M $TBAPF_6$), reduction potential at −0.7 V (vs Ag/AgCl) for 10 min, oxidation potential at +1.4 V (vs Ag/AgCl) for 10 min; (III) after one redox cycle of a chemically driven operation by using Cu dust and $NOPF_6$; (IV) after one redox cycle of a chemically driven operation by using Zn dust and $NOPF_6$; (V) after one redox cycle of a chemically driven operation by using Cp_2Co and $NOPF_6$

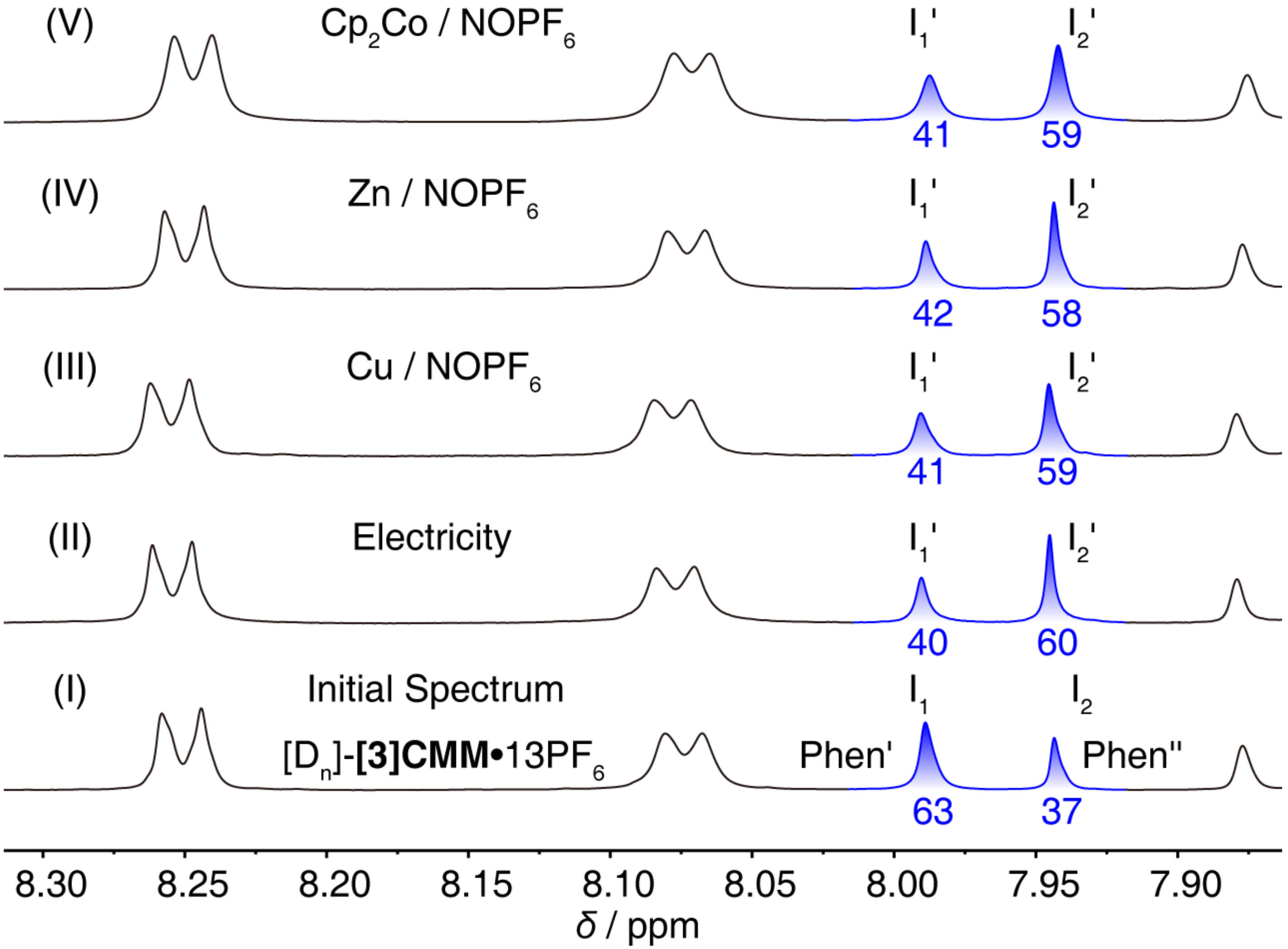

Supplementary Fig. 59 | Partial ^{1}H NMR spectra of [D$_n$]-**[3]CMM**•13PF$_6$. (I) Initial spectrum; (II) after one redox cycle of electrically driven operation. Conditions: [D$_n$]-**[3]CMM**•13PF$_6$ (4 mg) in MeCN (30 mL, 0.1 M TBAPF$_6$), reduction potential at −0.7 V (vs Ag/AgCl) for 10 min, oxidation potential at +1.4 V (vs Ag/AgCl) for 10 min; (III) after one redox cycle of a chemically driven operation by using Cu dust and NOPF$_6$; (IV) after one redox cycle of a chemically driven operation by using Zn dust and NOPF$_6$; (V) after one redox cycle of a chemically driven operation by using Cp$_2$Co and NOPF$_6$

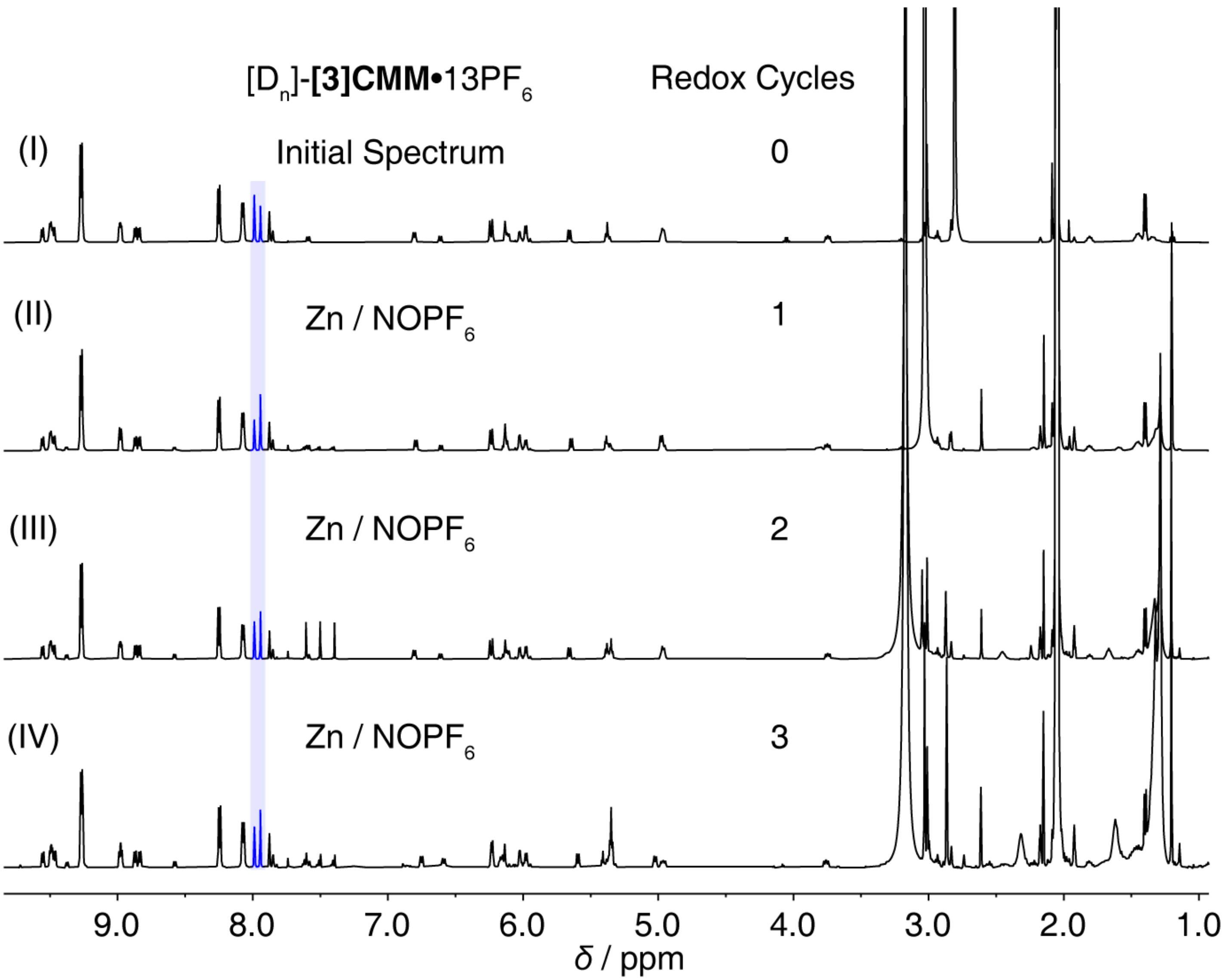

Supplementary Fig. 60 | ^{1}H NMR spectra of [D$_n$]-**[3]CMM**•13PF$_6$. (I) Initial spectrum; (II) / (III) / (IV) after 1 / 2 / 3 redox cycles of a chemically driven operation by using Zn dust and NOPF$_6$

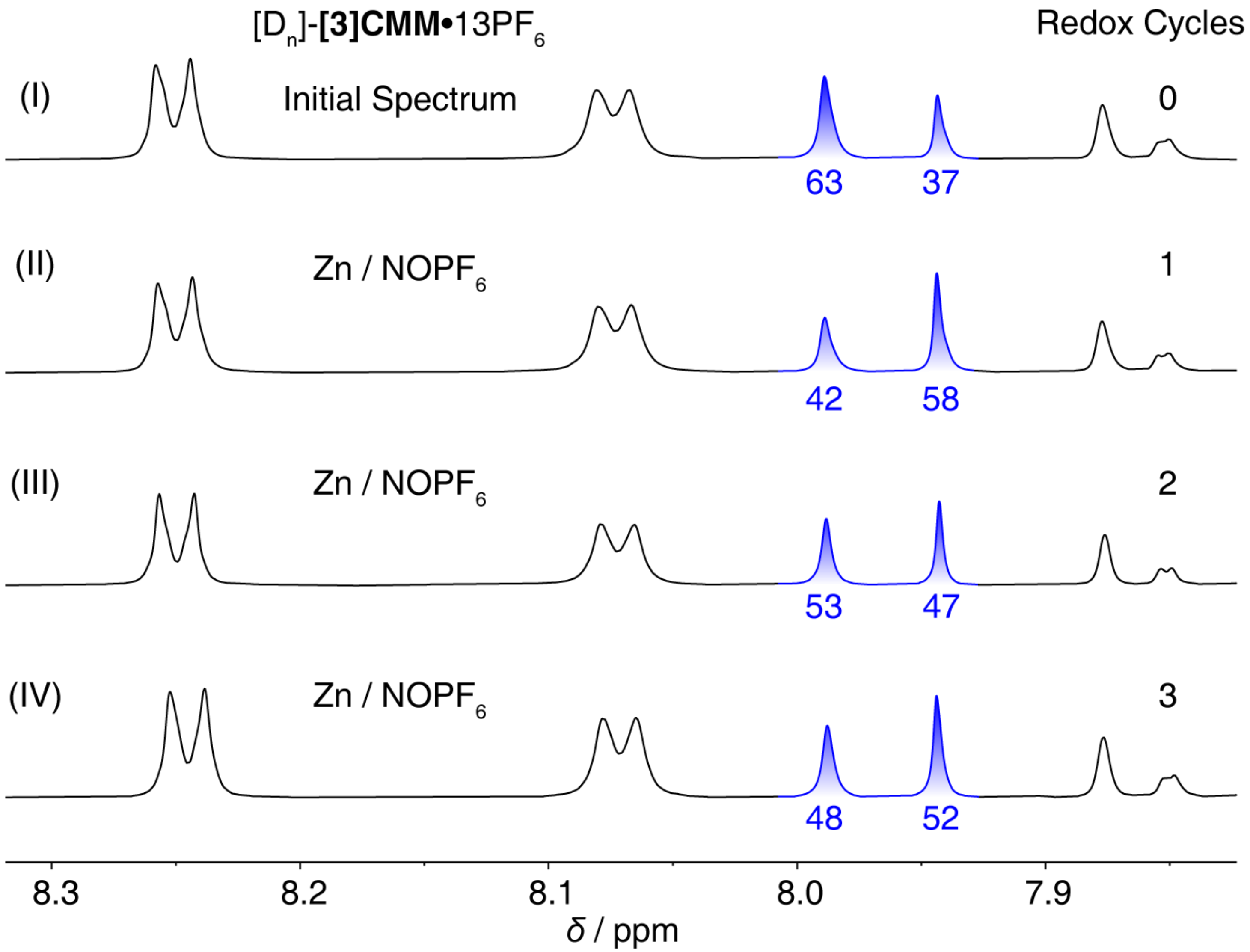

Supplementary Fig. 61 | Partial ^{1}H NMR spectra of [D$_n$]-**[3]CMM**•13PF$_6$. (I) Initial spectrum; (II) / (III) / (IV) after 1 / 2 / 3 redox cycles of a chemically driven operation by using Zn dust and NOPF$_6$

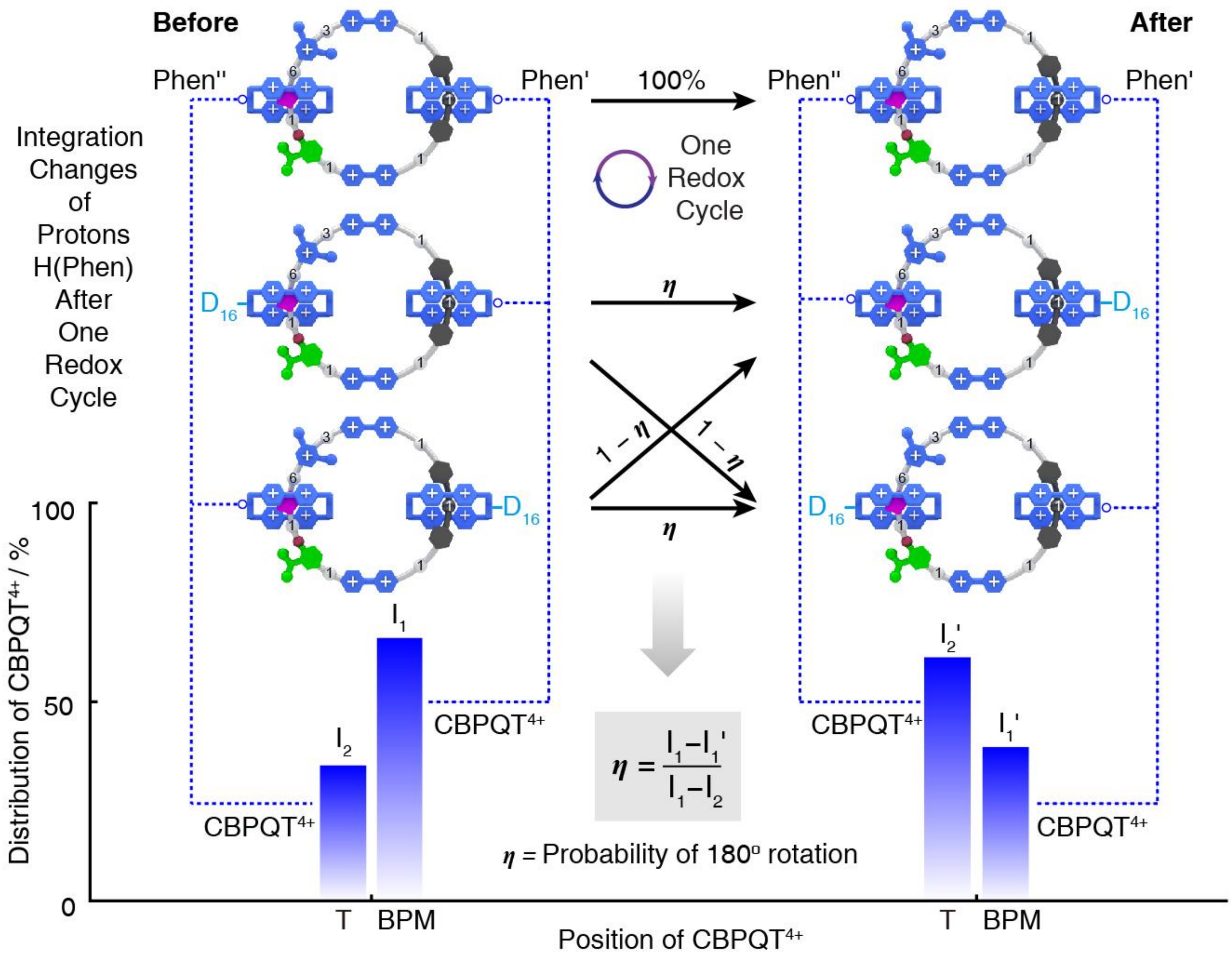

Supplementary Fig. 62 | Distribution of $CBPQT^{4+}$ rings on the two positions (T and BPM) of the cyclic track according to the ^{1}H NMR spectra (Supplementary Fig. 57) of [D_n]-**[3]CMM**•$13PF_6$ before and after one redox cycle, based on the relevant integration of protons H-Phen'' (I_1/I_1') and H-Phen' (I_2/I_2'), respectively. The probability of 180º rotation for both rings on the loop is represented by symbol $\boldsymbol{\eta}$.

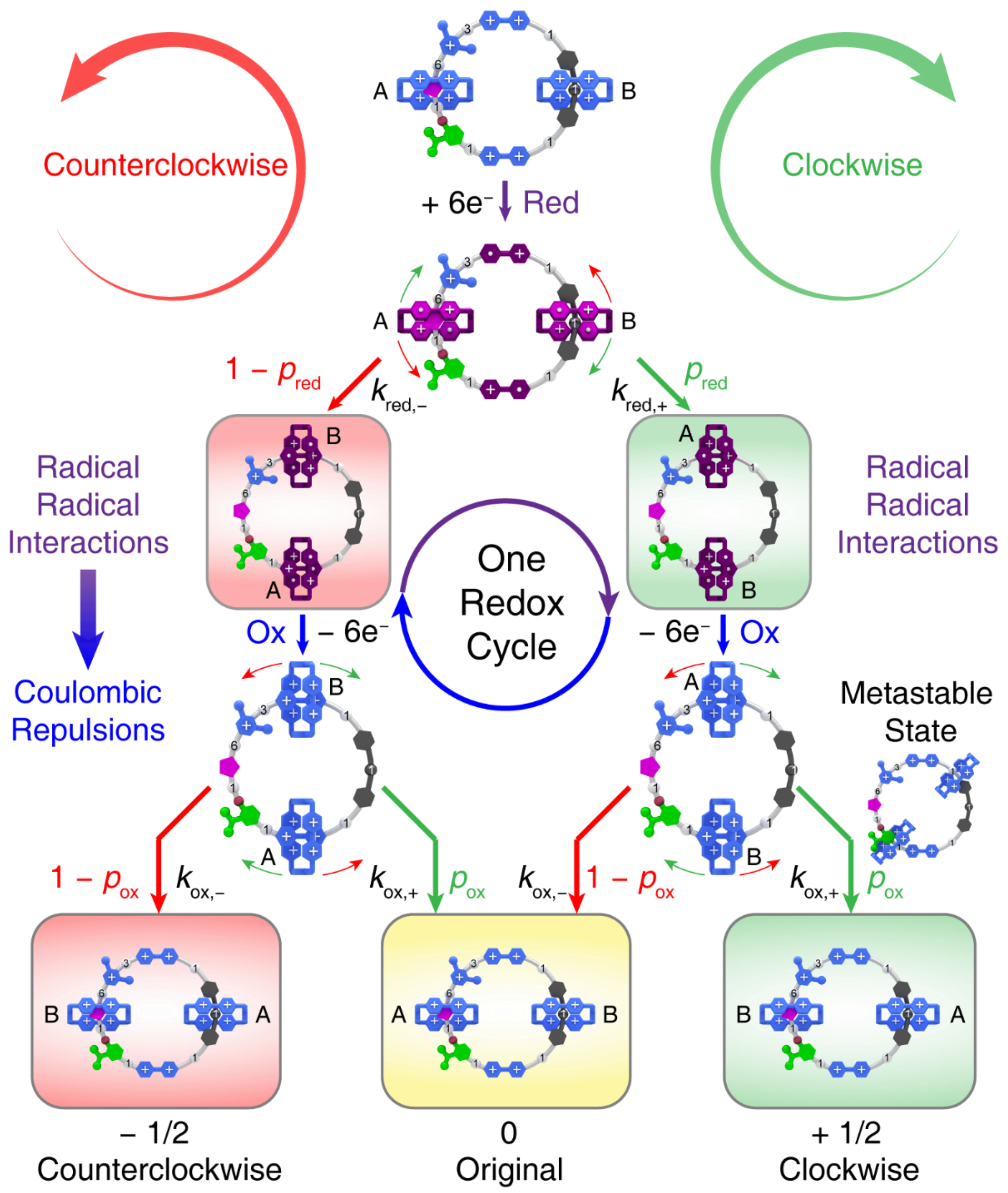

Supplementary Fig. 63 | A probability tree that shows the probable direction of movement of the two CBPQT$^{4+/2(+•)}$ rings during the redox operation of the [3]catenane molecular motor **[3]CMM^{13+}**. In order to simplify the discussion, we will loosely use the terms clockwise (CW) or counter-clockwise (CCW), but what we really mean by CW is that each CBPQT$^{4+/2(+•)}$ ring sees the substituents on the loop in the order T→PY^{+}→V$^{2+/+•}$→ V$^{2+/+•}$→IPP→T and by CCW that each ring sees the substituents on the loop in the order T→IPP → V$^{2+/+•}$→ V$^{2+/+•}$→PY^{+}→T. The curly arrows represent the direction in which the two rings move around the loop. The "p_{red}" and "p_{ox}" near the arrows are the probability of clockwise motion of the two rings on the loop in the redox cycle. The $k_{red,+}$, $k_{ox,-}$, $k_{ox,-}$, and $k_{ox,+}$ are the corresponding rate constant for the probable steps during the redox cycle, respectively, and the CW step and CCW are also indicated by "+" and "–", respectively. The 180° rotation is represented by "1/2".

In order to investigate whether there is any directionality in one redox cycle operation of the [3]catenane molecular motor **[3]CMM**, we have constructed a probability tree (Supplementary Fig. 63). In the trajectory ending at +1/2 each $CBPQT^{4+/2(+\bullet)}$ ring will have undergone clockwise rotation by 180°. This trajectory involves movement of a $CBPQT^{2(+\bullet)}$ ring over the PY^+ electrostatic barrier in the reduced state and movement of a $CBPQT^{4+}$ ring over the IPP steric barrier in the oxidized state. In the trajectory ending at −1/2 each $CBPQT^{4+/2(+\bullet)}$ ring will have undergone a counter-clockwise rotation by 180°. This trajectory involves motion of a $CBPQT^{2(+\bullet)}$ ring over the IPP steric barrier in the reduced state and motion of a $CBPQT^{4+}$ ring over the PY^+ electrostatic barrier in the oxidized state.

Calculation based on the ^{1}H NMR spectra:

According to the probability tree (Supplementary Fig. 63), the probability of 180° rotation $\boldsymbol{\eta}$ for the two $CBPQT^{4+/2(+\bullet)}$ rings on the loop in one redox cycle can be expressed as:

$$\boldsymbol{\eta} = (1 - p_{red})(1 - p_{ox}) + p_{red}p_{ox} = 1 - p_{red} - p_{ox} + 2p_{red}p_{ox}$$

wherein p_{red} and p_{ox} are the probability of clockwise motion of the two $CBPQT^{4+/2(+\bullet)}$ rings on the loop in the redox cycle, respectively.

From the result of the ^{1}H NMR spectroscopic studies (Supplementary Figs. 56–59) on the deuterium-labeled [3]catenane [D$_n$]-**[3]CMM**•13PF$_6$, we can calculate the probability of 180° rotation for two $CBPQT^{4+/2(+\bullet)}$ rings in one redox cycle to be 85%, so

$$\boldsymbol{\eta} = 1 - p_{red} - p_{ox} + 2p_{red}p_{ox} = 0.85$$

Then, we can obtain (Supplementary Fig. 64) the mathematical relationship between p_{red}, $p_{red}p_{ox}$ and p_{ox}, as shown below:

$$p_{red} = (p_{ox} - 0.15)/(2p_{ox} - 1)$$

$$p_{red}p_{ox} = (p_{ox}^2 - 0.15p_{ox})/(2p_{ox} - 1)$$

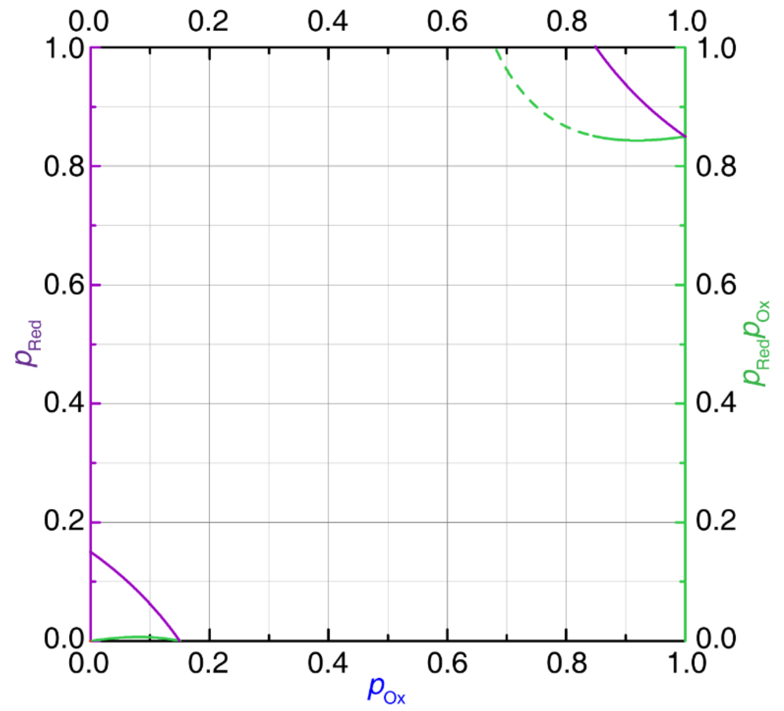

Supplementary Fig. 64 | Mathematic relationships of p_{ox}/p_{red} (purple traces) and $p_{ox}/p_{red}p_{ox}$ (solid green traces)

According to our calculations and previous investigations[2,3], the CBPQT$^{2(+\bullet)}$ ring does not pass over the IPP unit under reducing conditions, i.e., $1 - p_{red} \approx 0$. So,

$$p_{red} \approx 1$$

Then we can conclude (Supplementary Fig. 64) that

$$p_{ox} \approx p_{red}p_{ox} \approx 0.85$$

These calculations based on the observed switching probability provide strong evidence for the unidirectional motion and quantify the directionality to be 85% after one redox cycle.

Calculation based on energy differences in the kinetic barriers:

Because of the difference in passing a CBPQT^{4+} ring with +4 charges over a positively charge PY^{+} group versus passing a CBPQT$^{2(+\bullet)}$ ring with +2 charges over a positively charged PY^{+} group, we conclude that there must be a net clockwise rotation on account of sequential switching between oxidizing and reducing conditions, and that this directionality can be quite pronounced such that 85% of the two CBPQT$^{4+/2(+\bullet)}$ rings will make half a clockwise rotation for each oxidizing—

reducing—oxidizing switch as shown by the ^{1}H NMR spectroscopic studies on the deuterium-labeled [3]catenane [D$_n$]-**[3]CMM**•13PF$_6$. Note that the selection of direction is governed solely by kinetic asymmetry. The energy dissipation is the same for each of the four possible trajectories shown on the probability tree (Supplementary Fig. 63). We can also calculate the probability based on the rate constant ($k_{\mathrm{red},+}$, $k_{\mathrm{ox},-}$, $k_{\mathrm{ox},-}$, and $k_{\mathrm{ox},+}$) under reducing and oxidizing conditions.

Note that, the energy barriers we use here are from the DFT calculations (See Section 6 for details).

$$\Delta G_{\mathrm{red}} = 4.7\ \mathrm{kcal\ mol^{-1}}$$

$$\Delta G_{\mathrm{ox}} = 8.1\ \mathrm{kcal\ mol^{-1}}$$

The ratios of the rate constants can be written (T = 298 K, RT = 0.59 kcal mol^{-1})

$$\frac{k_{\mathrm{red},-}}{k_{\mathrm{red},+}} = e^{-\Delta G_{\mathrm{red}}/RT} = e^{-7.9}; \qquad \frac{k_{\mathrm{ox},-}}{k_{\mathrm{ox},+}} = e^{-\Delta G_{\mathrm{ox}}/RT} = e^{-13.7}.$$

The probability of a clockwise transition is

$$\frac{k_{\mathrm{red},+}k_{\mathrm{ox},+}}{k_{\mathrm{red},+}k_{\mathrm{ox},+} + k_{\mathrm{red},-}k_{\mathrm{ox},-} + k_{\mathrm{red},+}k_{\mathrm{ox},-} + k_{\mathrm{red},-}k_{\mathrm{ox},+}} = \frac{1}{1 + \frac{k_{\mathrm{red},-}k_{\mathrm{ox},-}}{k_{\mathrm{red},+}k_{\mathrm{ox},+}} + \frac{k_{\mathrm{ox},-}}{k_{\mathrm{ox},+}} + \frac{k_{\mathrm{red},-}}{k_{\mathrm{red},+}}} > 95\%$$

13. Metastable State and Kinetic Studies

General Procedure for Kinetic Studies: [3]CMM•$13PF_6$ (2.0 mg, 0.5 mmol) dissolved in degassed CD_3CN (1.0 mL). 6.0 equivalents of cobaltocene (Cp_2Co) were added to the solution and the mixture was stirred for 2 min at room temperature under N_2 atmosphere. The resulting dark purple solution was sealed in a NMR tube and analysed by ^1H NMR spectroscopy immediately after treated with a minimum amount of solid $NOPF_6$.

The ^1H NMR spectrum of the [3]catenane obtained immediately after the redox cycle is much more complex than that of the initial oxidized state **[3]CMM**•$13PF_6$, indicating the formation of a metastable intermediate species. Comparison of the two ^1H NMR spectra reveals (Supplementary Fig. 65) particularly large upfield shifts in the resonances associated with the protons H-22 / H-23 (IPP) and H-11 / H-12 (BPM), whereas resonances for protons H-14 and H-15 on the other phenyl ring of the BPM are shifted downfield. These observations suggest that one $CBPQT^{4+}$ ring is located asymmetrically on the BPM unit, while the other ring is poised to mount the IPP unit, indicating a synchronous and unidirectional motion of the two $CBPQT^{4+}$ ring moving away from the V^{2+} unit on account of the net Coulombic repulsion after oxidation of the reduced state **[3]CMM**$^{7+6\bullet}$. When the ^1H NMR spectrum was recorded at 253 K, no obvious change was detected (Supplementary Fig. 67) even after 5 h, indicating that the thermal energy at a low temperature is not sufficient to cause the co-conformational rearrangement to take place.

In a repetition of the experiment, the co-conformational rearrangement was followed (Supplementary Fig. 76) kinetically by recording ^1H NMR spectra at regular time intervals at 298 K. Kinetic analysis reveals (Supplementary Fig. 77 and 78) that this co-conformational rearrangement follows first-order kinetics at 298 K with an average rate constant k of $(8.6 \pm 0.4) \times 10^{-4}\ s^{-1}$, corresponding to an energy of activation ($\Delta G^{\ddagger}$) of 21.6 kcal mol^{-1} — a $\Delta G^{\ddagger}$ value which is comparable to that (21.8 kcal mol^{-1}) reported[3] previously for a molecular pump. It is worth noting that, in the kinetic studies, we define the starting time t = 0 as the time when we record the first ^1H NMR spectrum. Considering the fact that it takes some time to prepare the sample and record the first ^1H NMR spectrum, during this preparation process, some of the molecules in the metastable state have already relaxed to the final stable oxidized state.

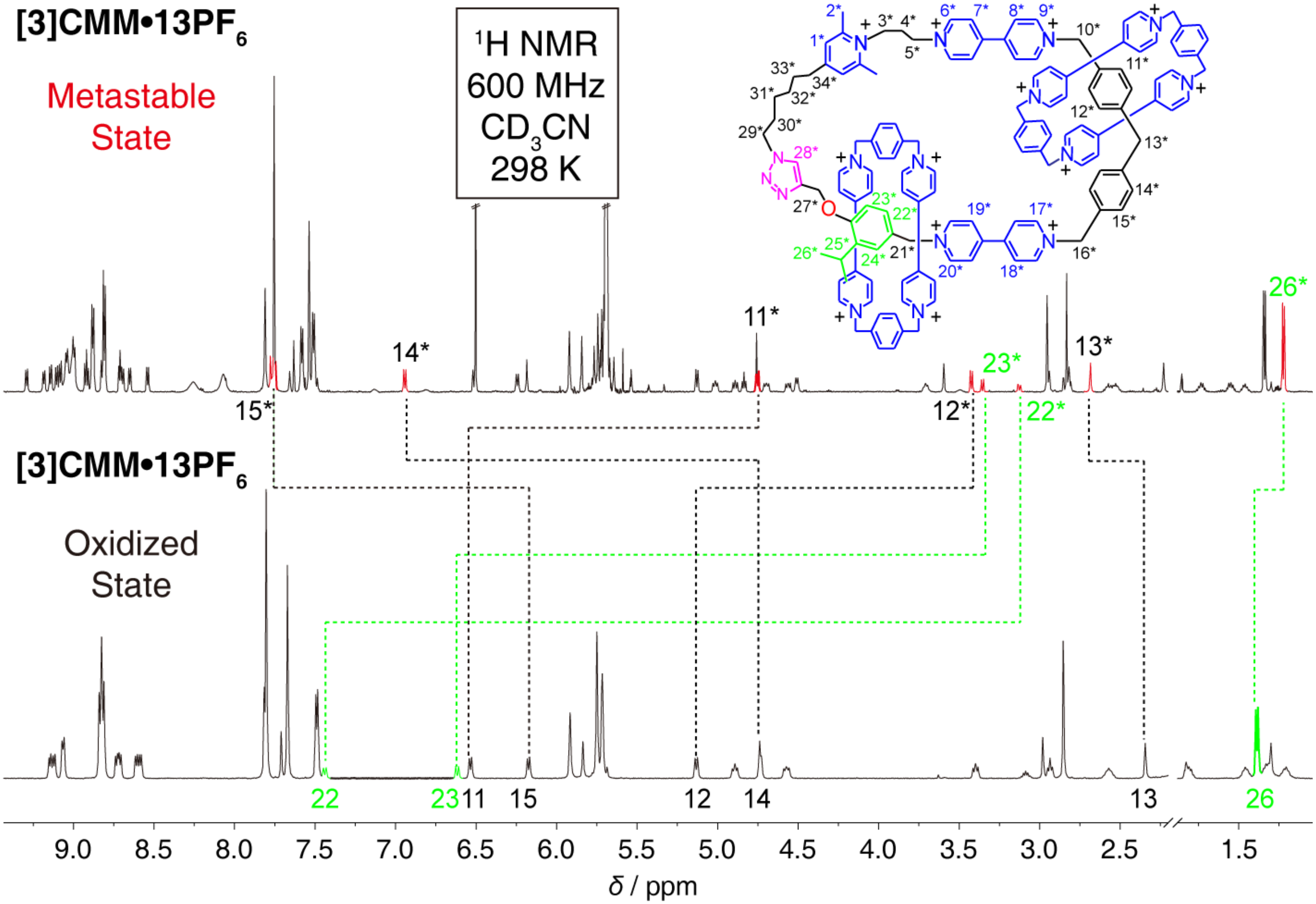

Supplementary Fig. 65 | Comparation of ^{1}H NMR spectra (600 MHz, CD_3CN, 298 K) of the [3]catenane **[3]CMM**•$13PF_6$ with proton assignments labeled. Bottom: Oxidized state. Top: Metastable state. The proton resonances attributable to the metastable state are labeled with an asterisk (*).

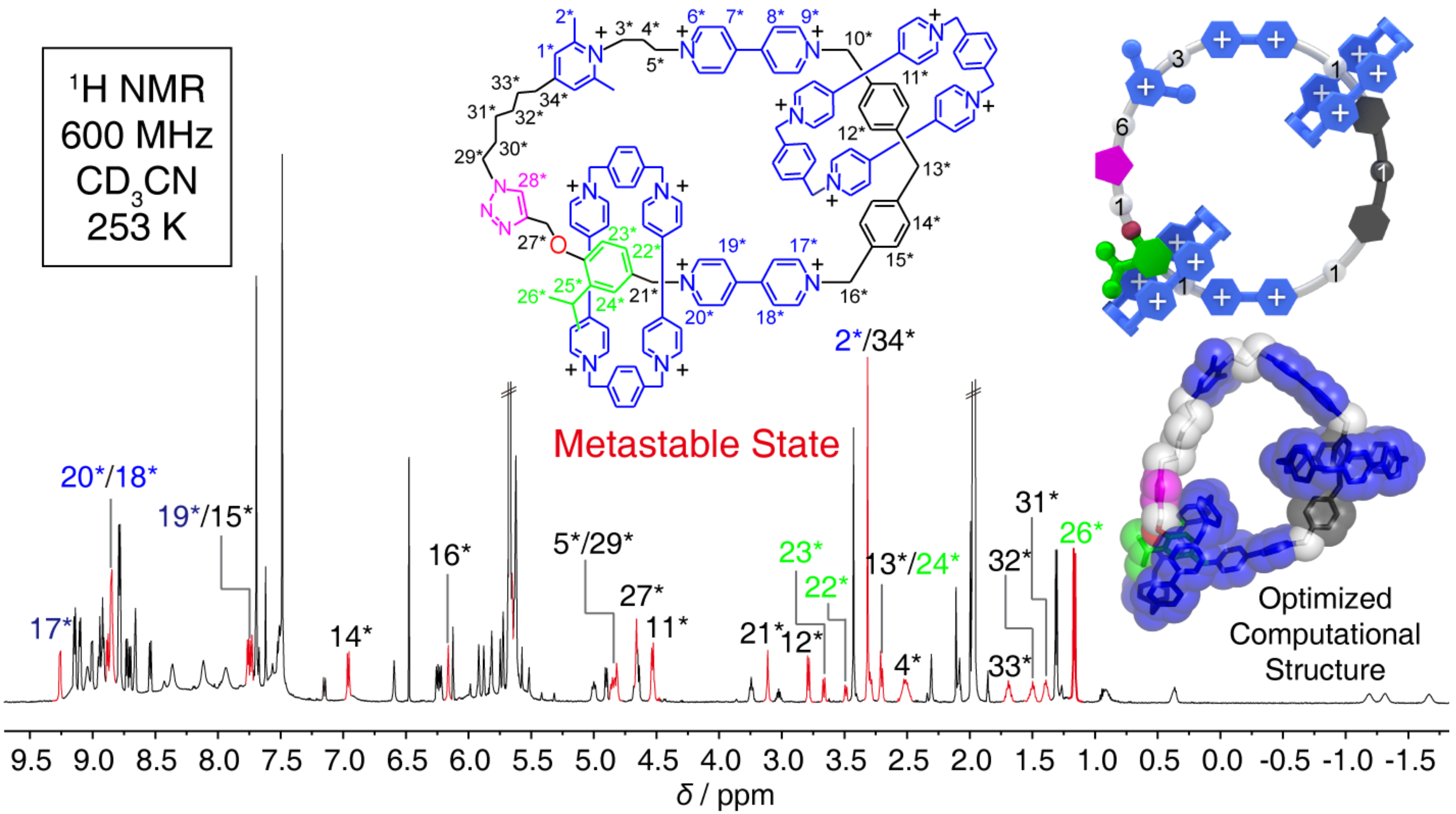

Supplementary Fig. 66 | (Top) Graphical representation and structural formula for the metastable state with an optimized quantum mechanical model structure (M06-2X/6-31G* basis set, in a tubular overlayed with a space-filling representation). (Bottom) ^{1}H NMR Spectrum (600 MHz, CD_3CN, 253 K) of the [3]catenane **[3]CMM**•$13PF_6$ measured immediately after reduction (Cp_2Co) and re-oxidation ($NOPF_6$). The proton resonances (red) attributable to the metastable state are labeled with an asterisk (*).

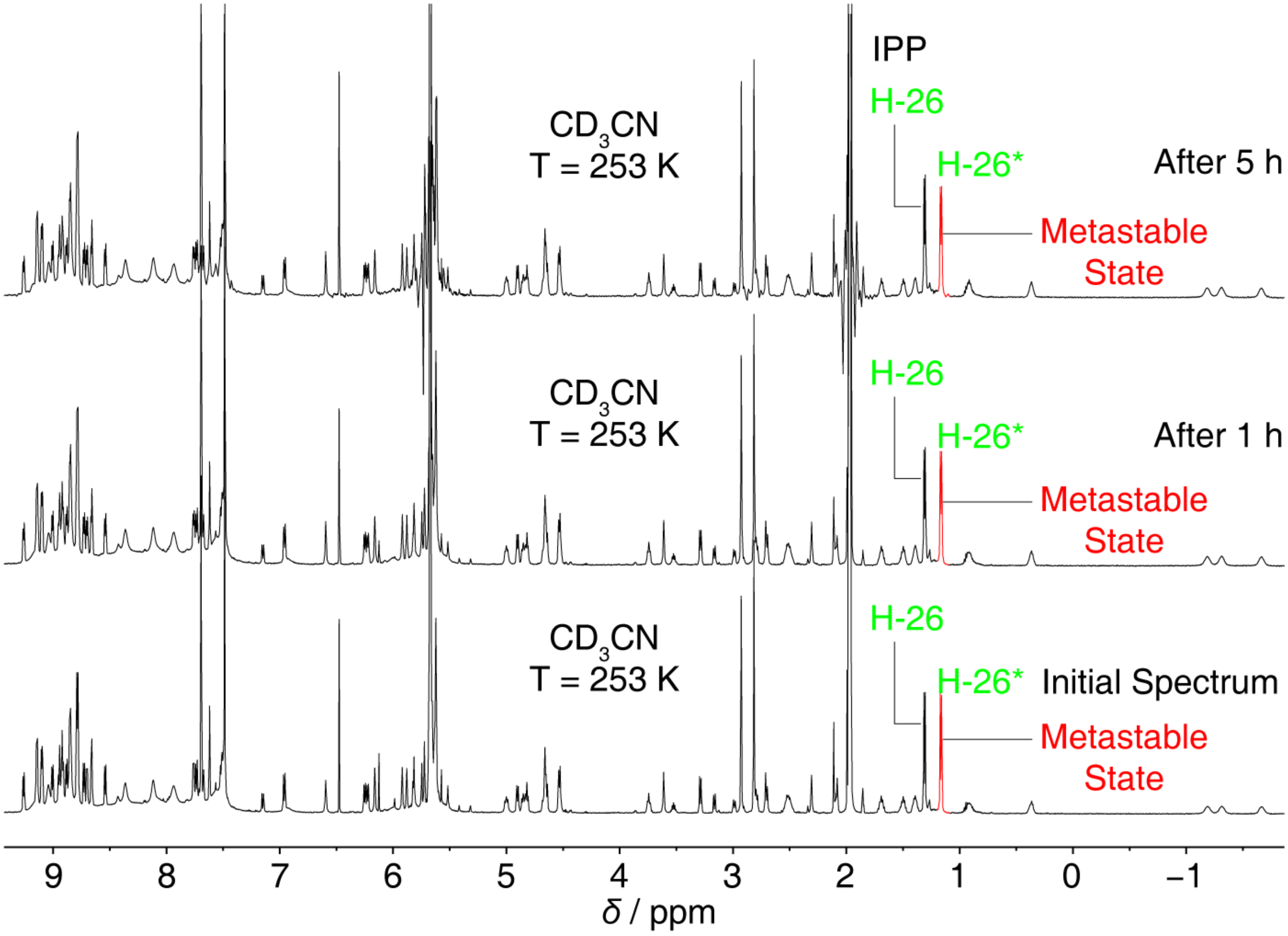

Supplementary Fig. 67 | ^{1}H NMR Spectra (600 MHz, CD_3CN, 253 K) of the [3]catenane **[3]CMM**•$13PF_6$ measured immediately after reduction (Cp_2Co) and re-oxidation ($NOPF_6$)

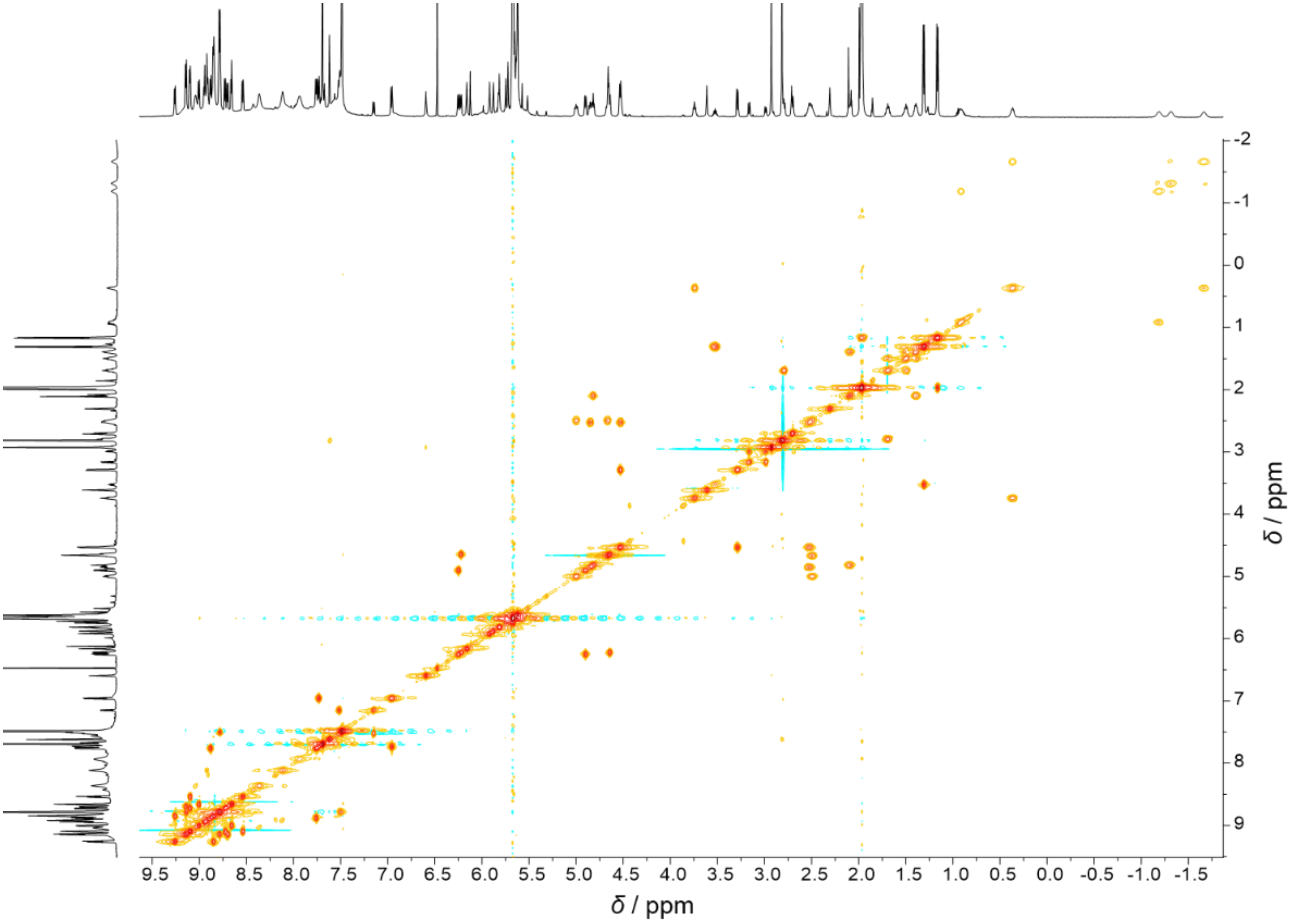

Supplementary Fig. 68 | ^{1}H-^{1}H COSY NMR Spectrum (600 MHz, CD_3CN, 253 K) of **[3]CMM**•13PF_6 measured immediately after reduction (reduced by Cp_2Co) and oxidation (oxidized by $NOPF_6$)

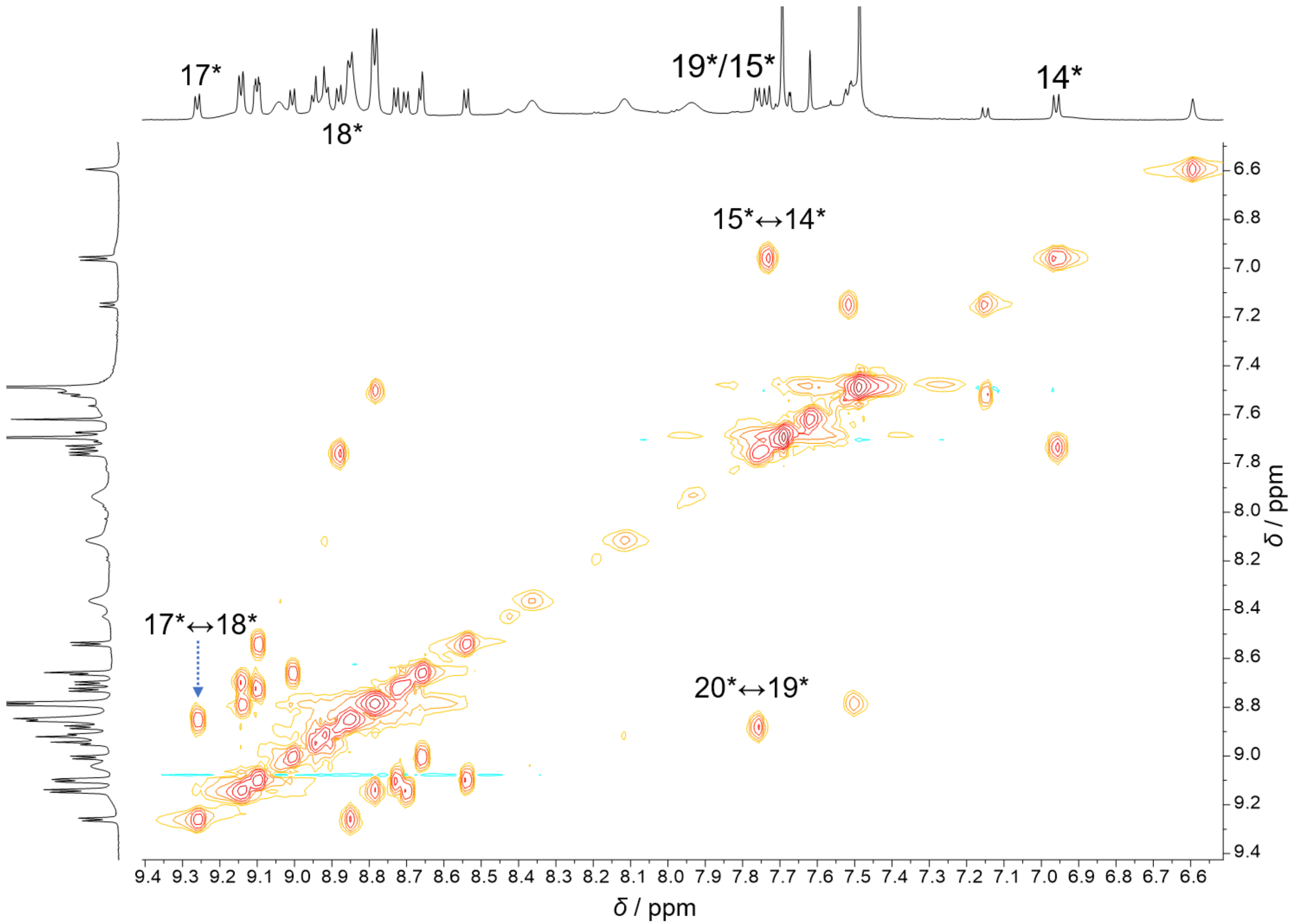

Supplementary Fig. 69 | Partial ^1H-^1H COSY NMR spectrum (600 MHz, CD_3CN, 253 K) of **[3]CMM**•13PF_6 (* indicates the proton signals of metastable species) measured immediately after reduction (reduced by Cp_2Co) and oxidation (oxidized by $NOPF_6$)

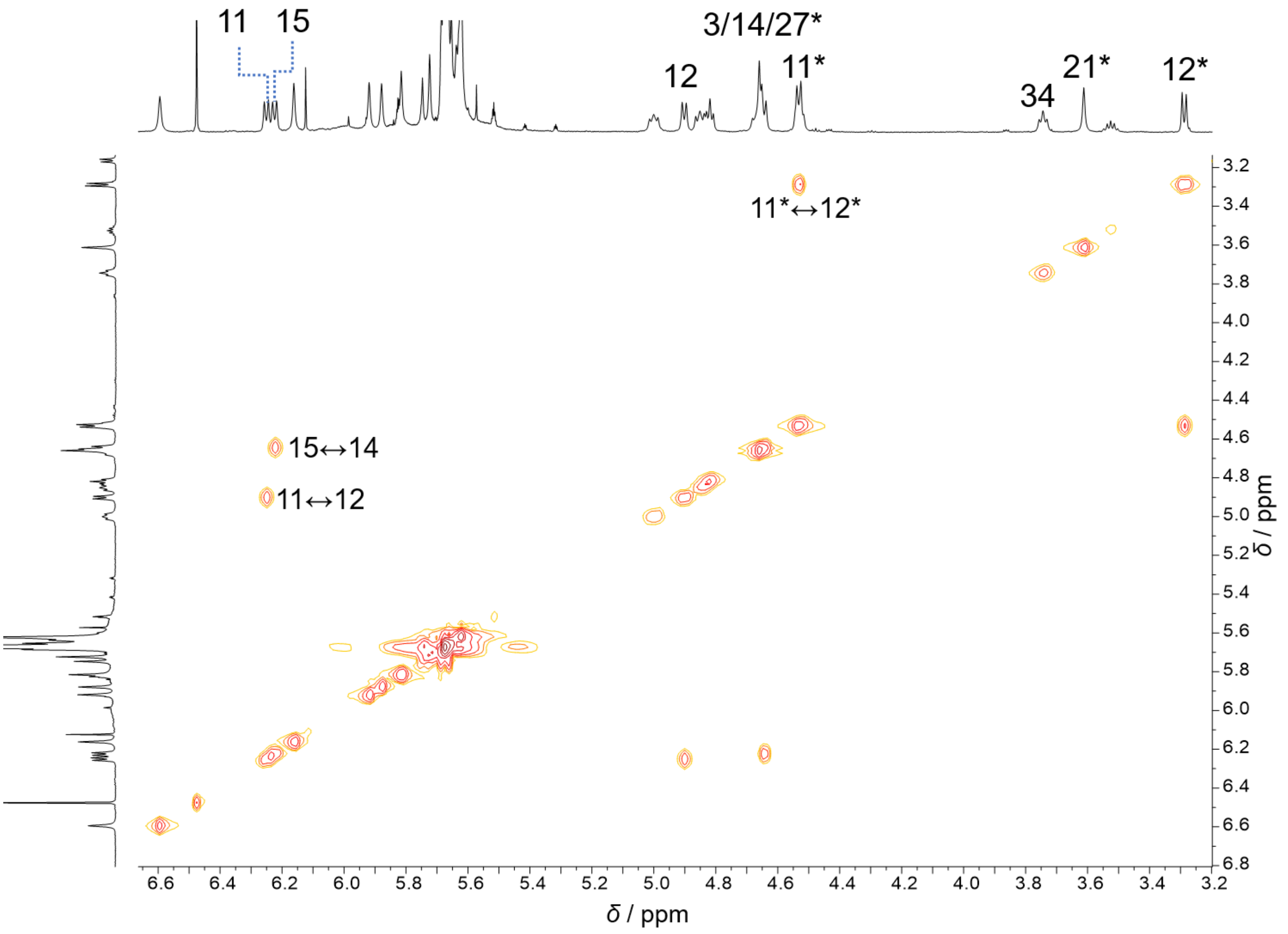

Supplementary Fig. 70 | Partial ^{1}H-^{1}H COSY NMR spectrum (600 MHz, CD_3CN, 253 K) of **[3]CMM**•13PF_6 (* indicates the proton signals of metastable species) measured immediately after reduction (reduced by Cp_2Co) and oxidation (oxidized by $NOPF_6$)

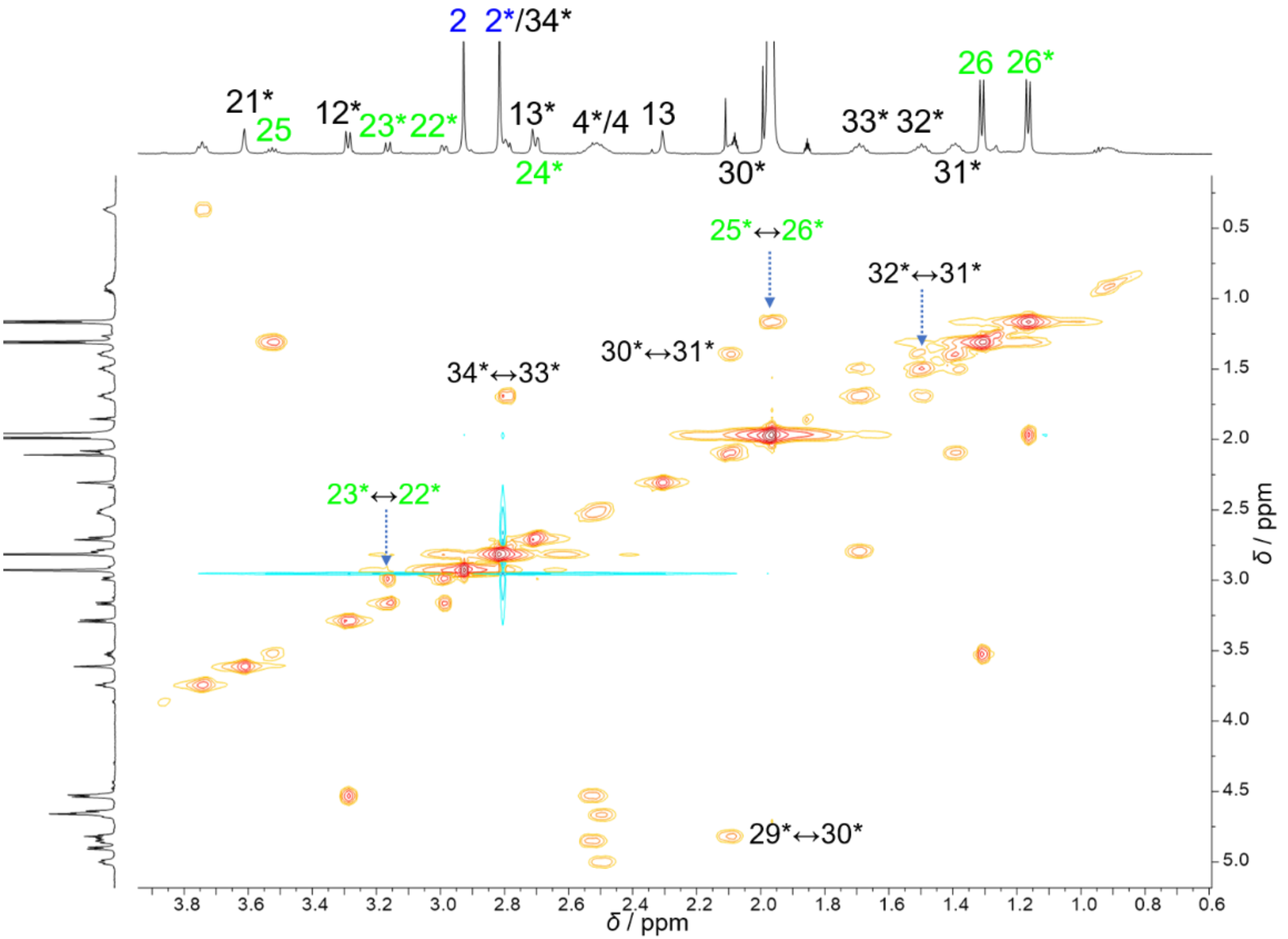

Supplementary Fig. 71 | Partial ^{1}H-^{1}H COSY NMR spectrum (600 MHz, CD_3CN, 253 K) of **[3]CMM**•$13PF_6$ (* indicates the proton signals of metastable species) measured immediately after reduction (reduced by Cp_2Co) and oxidation (oxidized by $NOPF_6$)

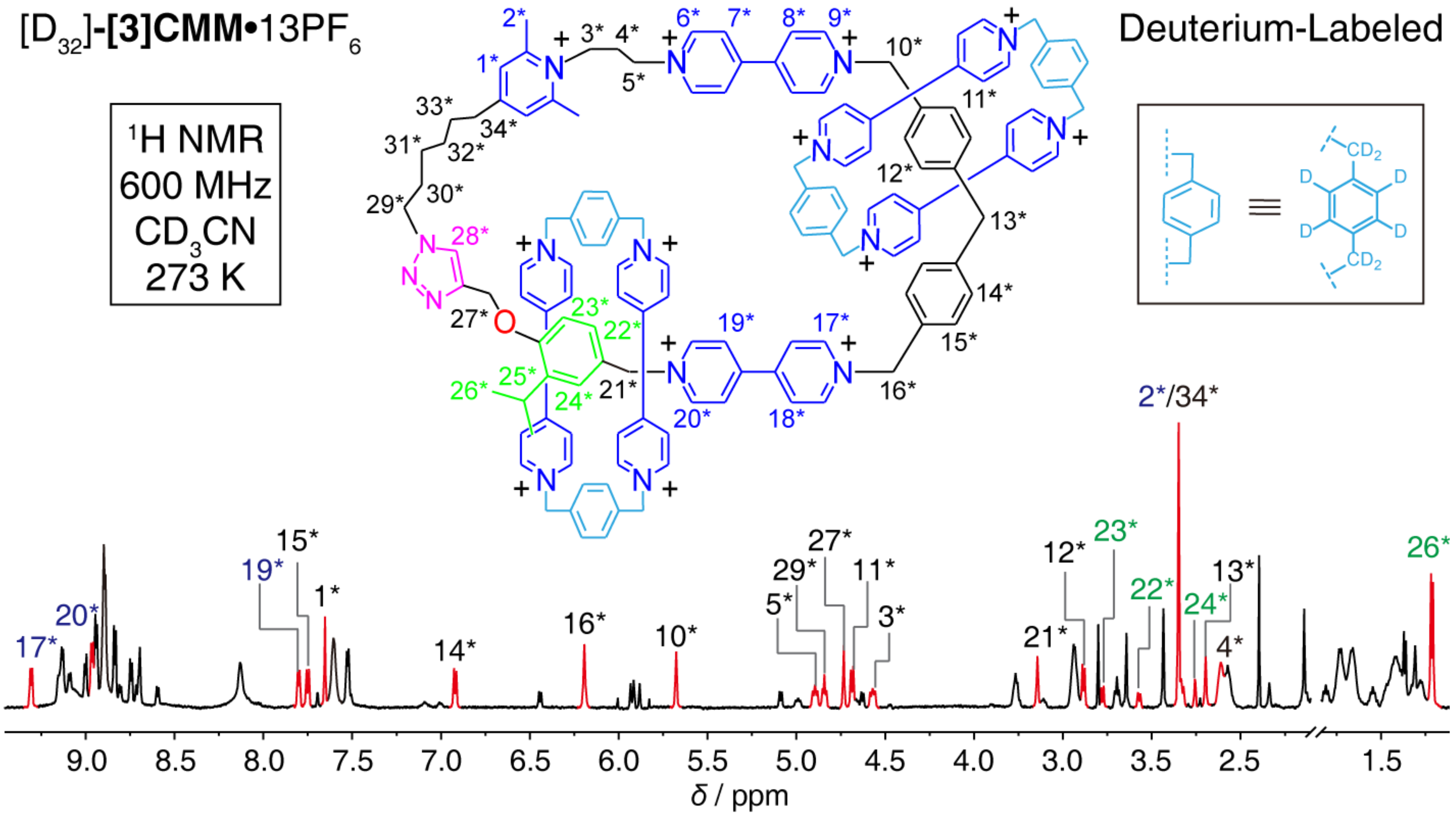

Supplementary Fig. 72 | ^{1}H NMR Spectrum (600 MHz, CD_3CN, 273 K) of $[D_{32}]$-**[3]CMM**•$13PF_6$ measured immediately after reduction (Cu dust) and oxidation ($NOPF_6$). The proton resonances attributable to the metastable state are labeled with an asterisk (*).

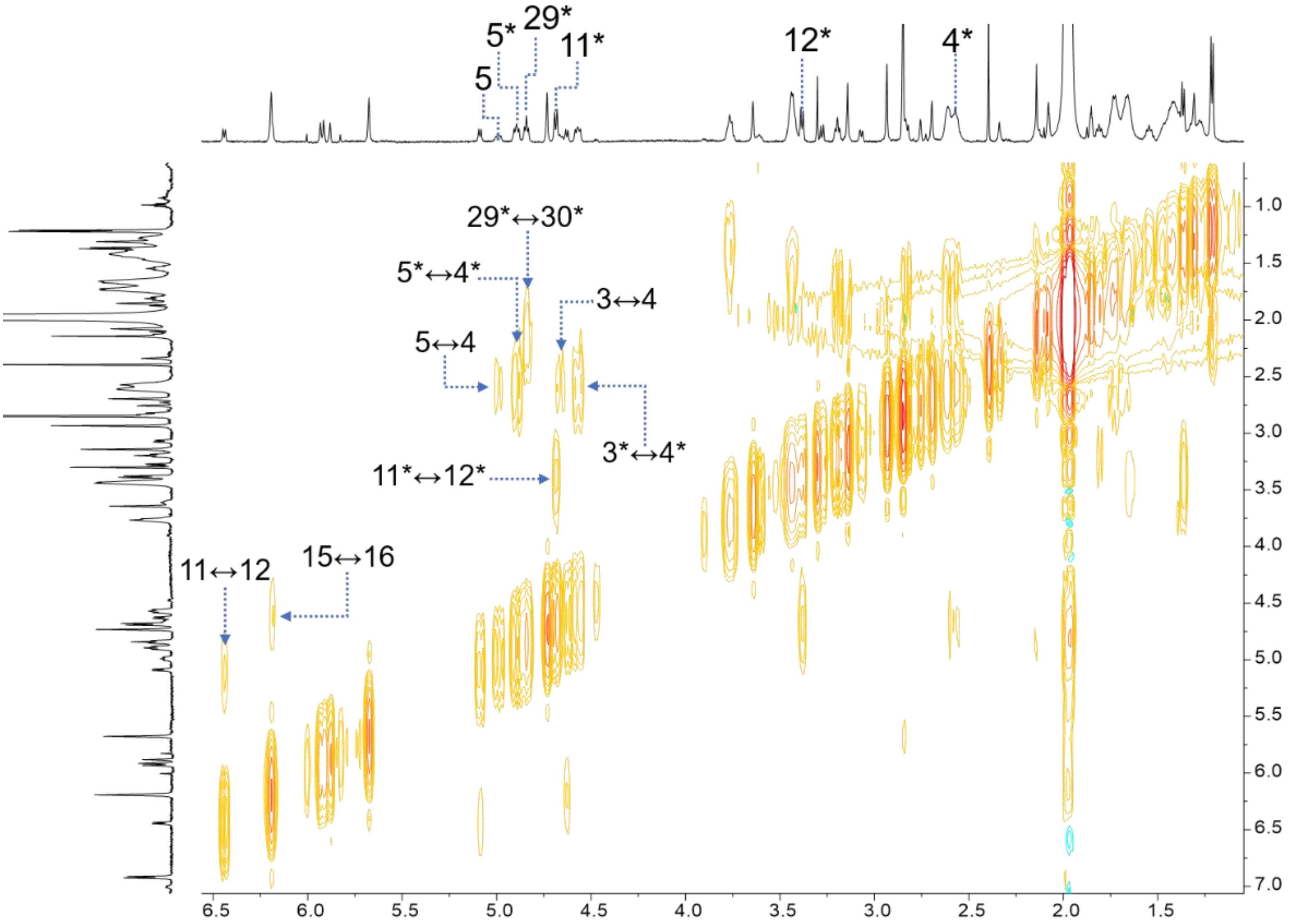

Supplementary Fig. 73 | Partial ^{1}H-^{1}H COSY NMR spectrum (600 MHz, CD_3CN, 273 K) of [D_{32}]-**[3]CMM**•13PF_6 (* indicates the proton signals of metastable species) measured immediately after reduction (reduced by Cu dust) and oxidation (oxidized by $NOPF_6$)

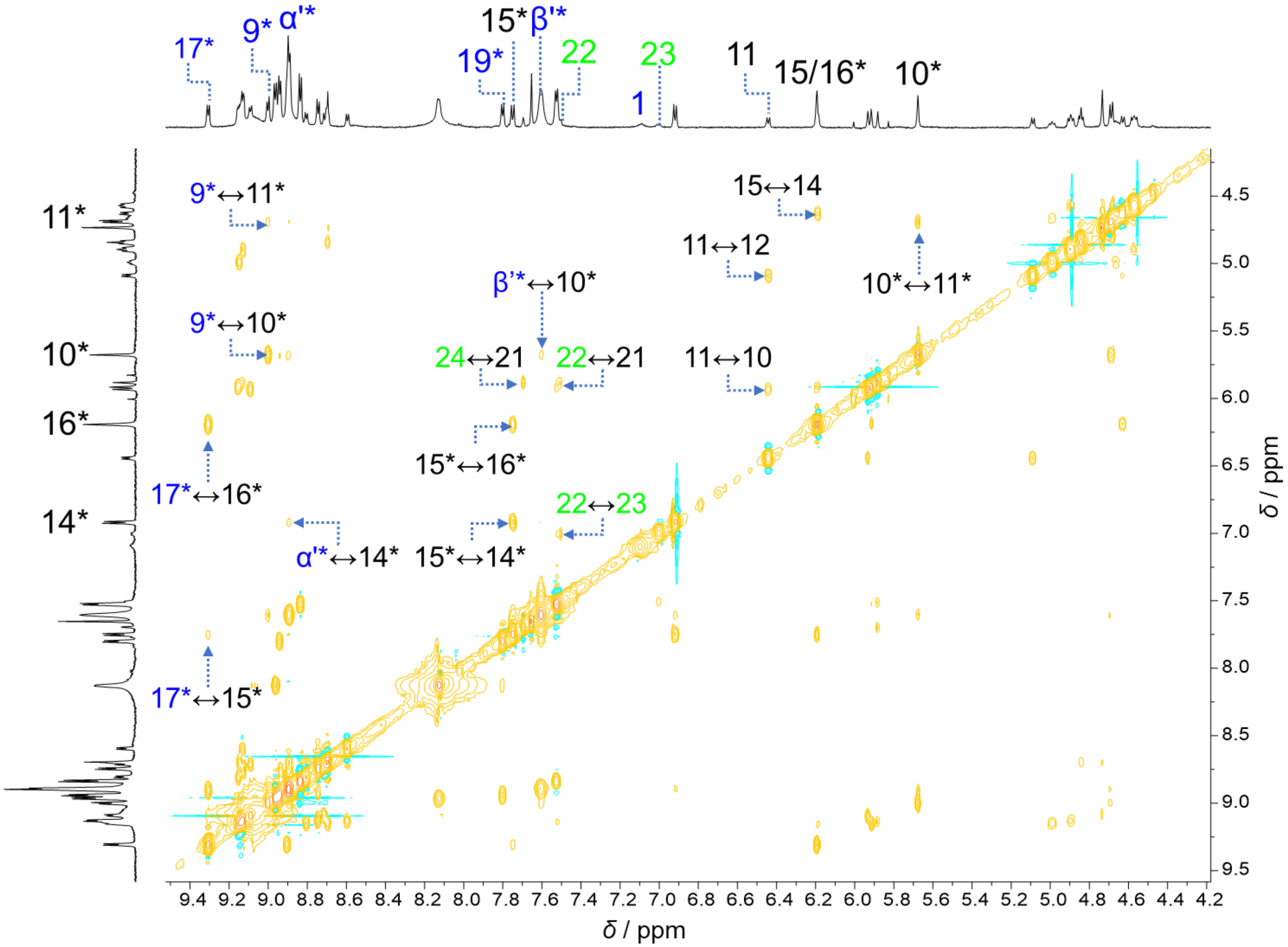

Supplementary Fig. 74 | Partial ^{1}H-^{1}H NOESY NMR spectrum (600 MHz, CD_3CN, 273 K) of [D_{32}]-**[3]CMM**•13PF_6 (* indicates the proton signals of metastable species) measured immediately after reduction (reduced by Cu dust) and oxidation (oxidized by $NOPF_6$)

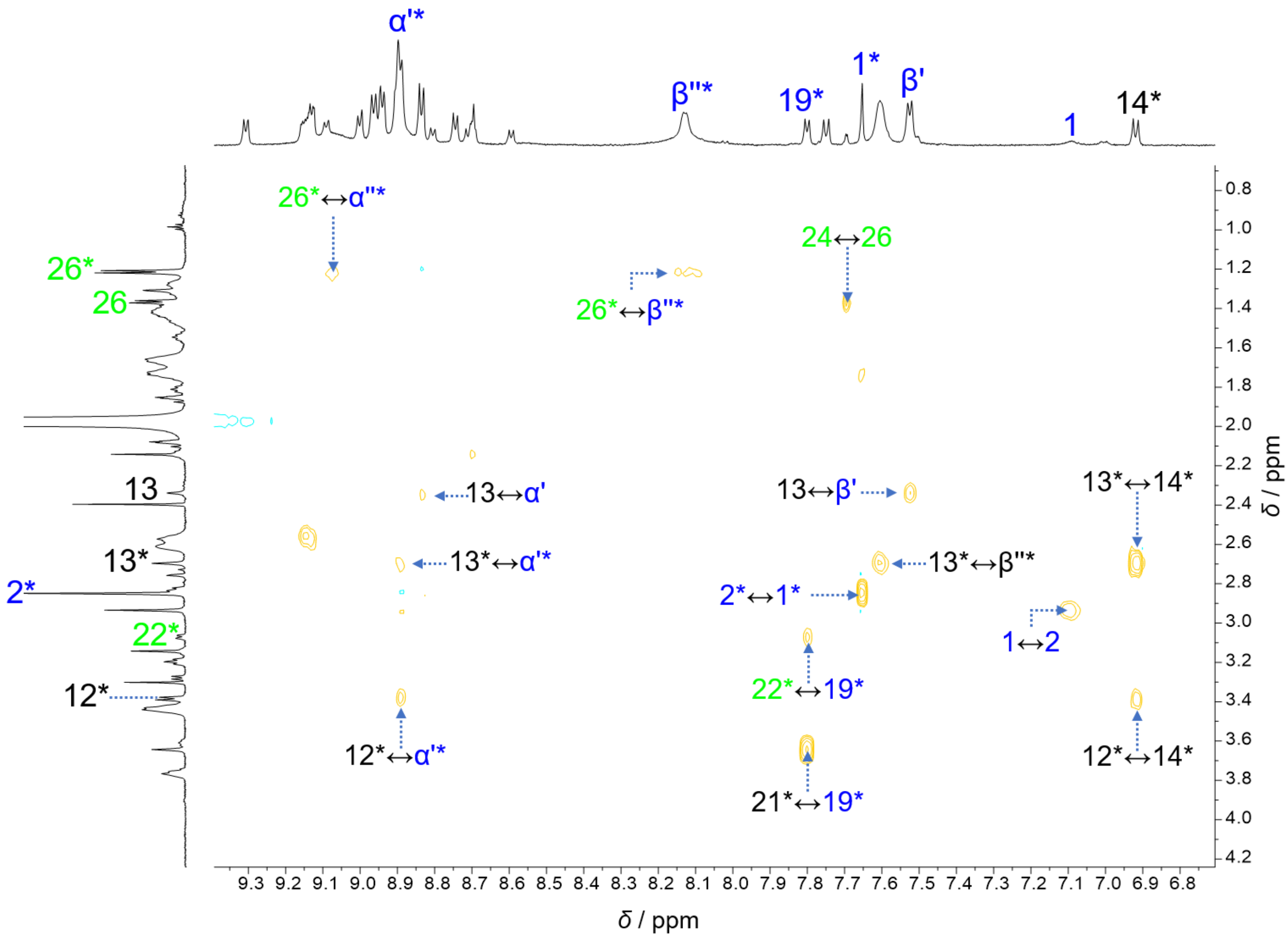

Supplementary Fig. 75 | Partial ^{1}H-^{1}H NOESY NMR spectrum (600 MHz, CD_3CN, 273 K) of [D_{32}]-**[3]CMM**•$13PF_6$ (* indicates the proton signals of metastable species) measured immediately after reduction (reduced by Cu dust) and oxidation (oxidized by $NOPF_6$)

Kinetic Studies

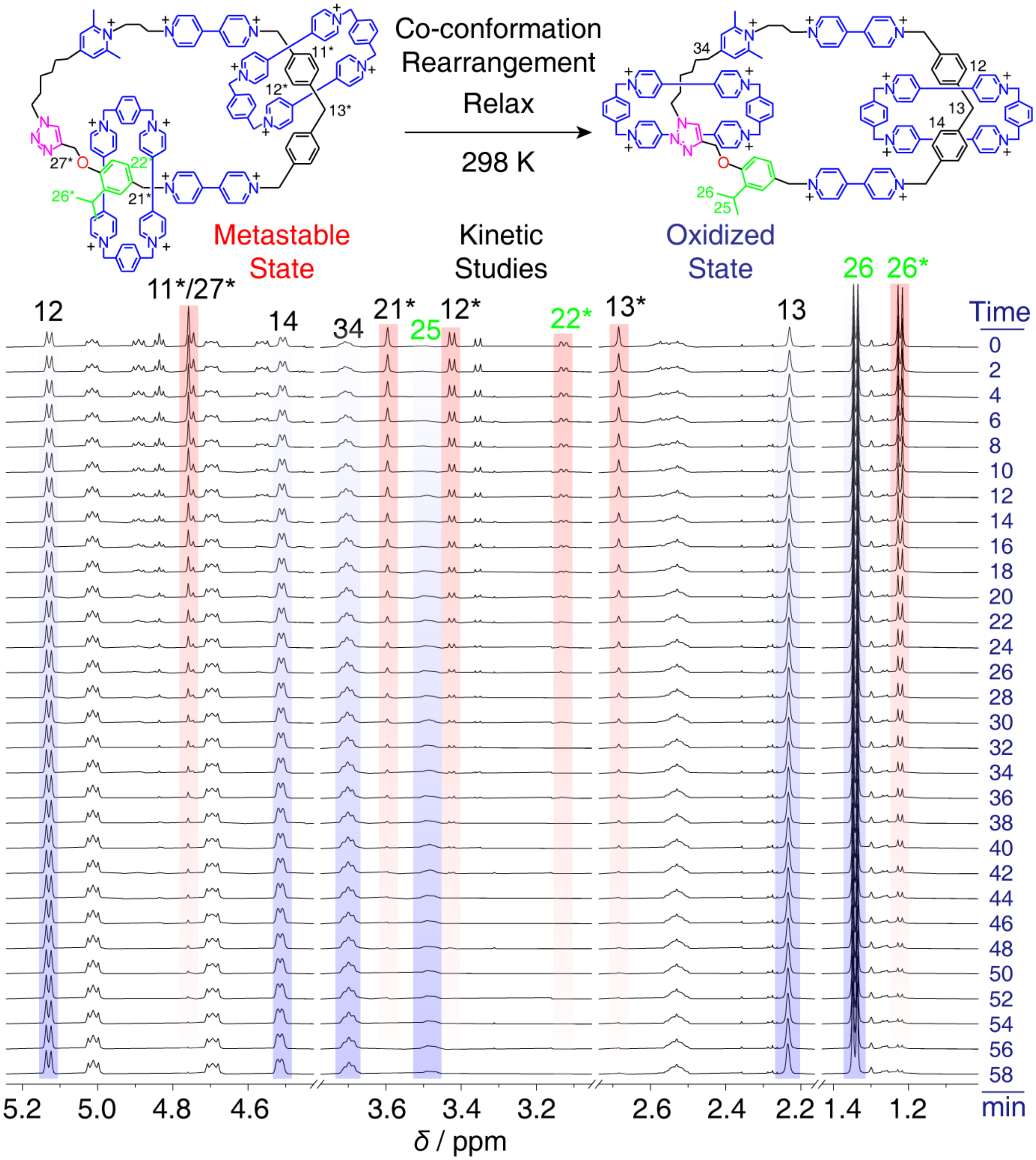

Supplementary Fig. 76 | Kinetics of the co-conformational rearrangment measured by recording multiple ^{1}H NMR spectra (600 MHz, CD_3CN, 298 K) of the [3]catenane **[3]CMM**•13PF_6 over time (0–58 min) immediately after a cycle of reduction (Cp_2Co) and re-oxidation ($NOPF_6$), with proton assignments labeled at the top of the spectral display. The proton resonances attributable to the metastable state are labeled with an asterisk (*).

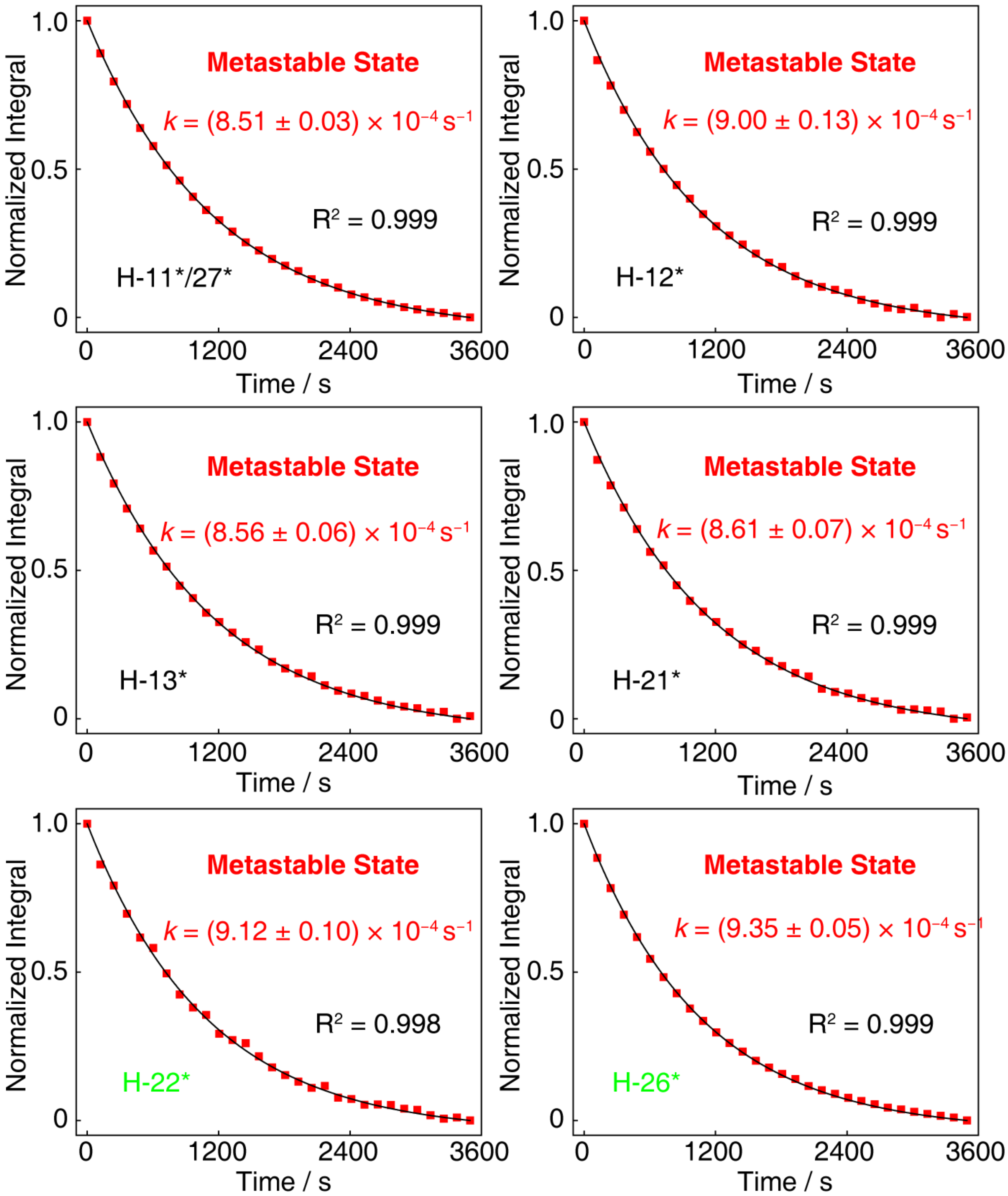

Supplementary Fig. 77 | The kinetic plots for the related metastable species of the [3]catenane **[3]CMM•**$13PF_6$ during the thermal relaxation process. By plotting the change in molar fraction over time at 298K, based on the integration of protons H-11*/27*, H-12*, H13*, H-21*, H-22*, and H-26* in the ^{1}H NMR spectra

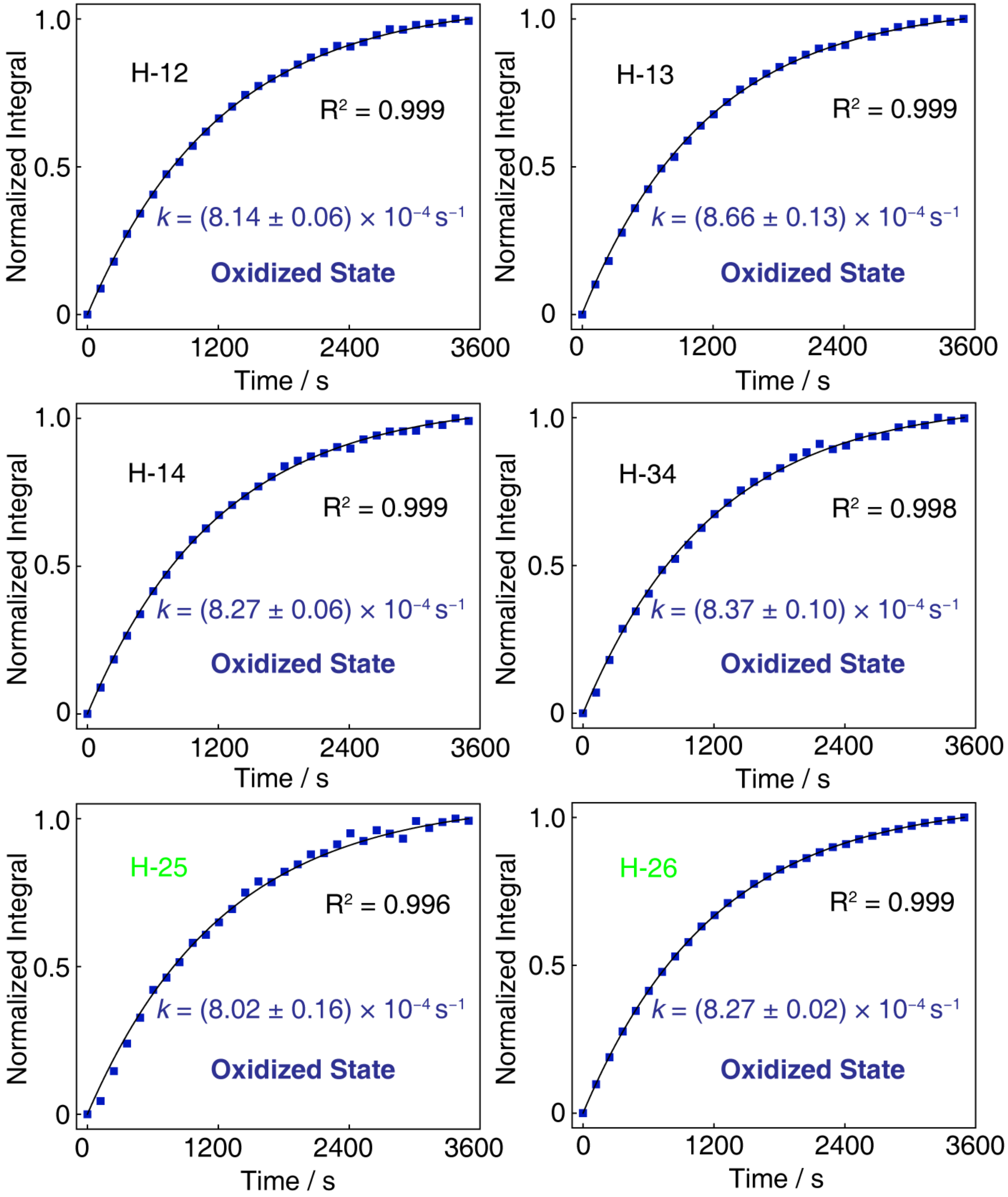

Supplementary Fig. 78 | The kinetic plots for the related re-oxidized species of the [3]catenane **[3]CMM**•$13PF_6$ during the thermal relaxation process. By plotting the change in molar fraction over time at 298 K, based on the integration of protons H-12, H-13, H14, H-25, H-26, and H-34 in the ^{1}H NMR spectra

14. References

1. Odell, B. *et al.* Cyclobis(paraquat-*p*-phenylene). A tetracationic multipurpose receptor. *Angew. Chem. Int. Ed. Engl.* **27**, 1547–1550 (1988).
2. Qiu, Y. *et al.* A molecular dual pump. *J. Am. Chem. Soc.* **141**, 17472–17476 (2019).
3. Pezzato, C. *et al.* An efficient artificial molecular pump. *Tetrahedron* **73**, 4849–4857 (2017).
4. Peng, K.-Y., Chen, S.-A. & Fann, W.-S. Efficient light harvesting by sequential energy transfer across aggregates in polymers of finite conjugational segments with short aliphatic linkages. *J. Am. Chem. Soc.* **123**, 11388–11397 (2001).
5. Nag, O. K. *et al.* Two-photon absorption properties of cationic 1,4-bis(styryl)benzene derivative and its inclusion complexes with cyclodextrins. *J. Phys. Chem. B* **114**, 9684–9690 (2010).
6. Thalassinos, K. *et al.* Characterization of phosphorylated peptides using traveling wave-based and drift cell ion mobility mass spectrometry. *Anal. Chem.* **81**, 248–254 (2009).
7. https://clemlab.sitehost.iu.edu/Research/Cross%20Section%20Database/Proteins/protein_cs.htm
8. Tannor, D. J. *et al.* Accurate first principles calculation of molecular charge distributions and solvation energies from ab initio quantum mechanics and continuum dielectric theory. *J. Am. Chem. Soc.* **116**, 11875–11882 (1994).
9. Zhao, Y. & Truhlar, D. G. The M06 suite of density functionals for main group thermochemistry, thermochemical kinetics, noncovalent interactions, excited states, and transition elements: Two new functionals and systematic testing of four M06-class functionals and 12 other functionals. *Theor. Chem. Acc.* **120**, 215–241 (2007).
10. Jaguar, Version 10.6 (Schrodinger, Inc., New York, 2019).
11. Dolomanov, O. V., Bourhis, L. J., Gildea, R. J., Howard, J. A. K. & Puschmann, H. OLEX2: a complete structure solution, refinement and analysis program. *J. Appl. Cryst.* **42**, 339–341 (2009).
12. Sheldrick, G. M. *SHELXT* – Integrated space-group and crystal-structure determination. *Acta Cryst*. A**71**, 3–8 (2015).
13. Sheldrick, G. M. A short history of *SHELXT*. *Acta Cryst*. A**64**, 112–122 (2008).
14. Thorn, A., Dittrich, B. & Sheldrick, G. M. Enhanced rigid-bond restraints. *Acta Cryst.* A**68**, 448–451 (2012).
15. Li, H. *et al.* Mechanical bond-induced radical stabilization. *J. Am. Chem. Soc.* **135**, 456−467 (2013).
16. Jiao, Y. *et al.* Electron-catalysed molecular recognition. *Nature* **603**, 265–270 (2022).
17. Pezzato, C. *et al.* Controlling dual molecular pumps electrochemically. *Angew. Chem. Int. Ed.* **57**, 9325–9329 (1988).
18. Cai, K. *et al.* Radical cyclic[3]daisy chains. *Chem*, **7**, 174–189 (2021).
